# Separation of Alkyne Enantiomers by Chiral Column HPLC Analysis of Their Cobalt-Complexes

**DOI:** 10.3390/molecules22030466

**Published:** 2017-03-20

**Authors:** Qiaoyun Liu, Jing Wang, Junfei Li, Xiaolei Wang, Shichao Lu, Xuan Li, Yaling Gong, Shu Xu

**Affiliations:** 1 School of Chemistry and Material Science, Shanxi Normal University, 1 Gongyuan Street, Linfen, Shanxi 041004, China; liuqy@sxnu.edu.cn (Q.L.); 214111062@stu.sxnu.edu.cn (J.W.); lijunfei@sxnu.edu.cn (J.L.); 2 State Key Laboratory of Bioactive Substance and Function of Natural Medicines, Beijing Key Laboratory of Active Substances Discovery and Drugability Evaluation, Institute of Materia Medica, Chinese Academy of Medical Sciences and Peking Union Medical College, 2A NanWei Road, Xicheng Distrct, Beijing 100050, China; wangxiaolei@imm.ac.cn (X.W.); lushichao@imm.ac.cn (S.L.); lanthanum1979@gmail.com (X.L.)

**Keywords:** alkyne, cobalt complex, HPLC, chiral column, resolution

## Abstract

Separation of the enantiomers of new chiral alkynes in strategic syntheses and bioorthogonal studies is always problematic. The chiral column high-performance liquid chromatography (HPLC) method in general could not be directly used to resolve such substrates, since the differentiation of the alkyne segment with the other alkane/alkene segment is not significant in the stationary phase, and the alkyne group is not a good UV chromophore. Usually, a pre-column derivatization reaction with a tedious workup procedure is needed. Making use of easily-prepared stable alkyne-cobalt-complexes, we developed a simple and general method by analyzing the in situ generated cobalt-complex of chiral alkynes using chiral column HPLC. This new method is especially suitable for the alkynes without chromophores and other derivable groups.

## 1. Introduction

Alkyne is one of the fundamental groups in organic chemistry, which exists widely in natural products and unnatural functionalized molecules [1]. The development of modern transition-metal-catalyzed reactions, such as cross coupling [2], Pauson-Khand reaction [3], click chemistry [4], etc., has caused alkynes to play a more and more important role in strategic syntheses and bioorthogonal design [1].

**Figure 1:**
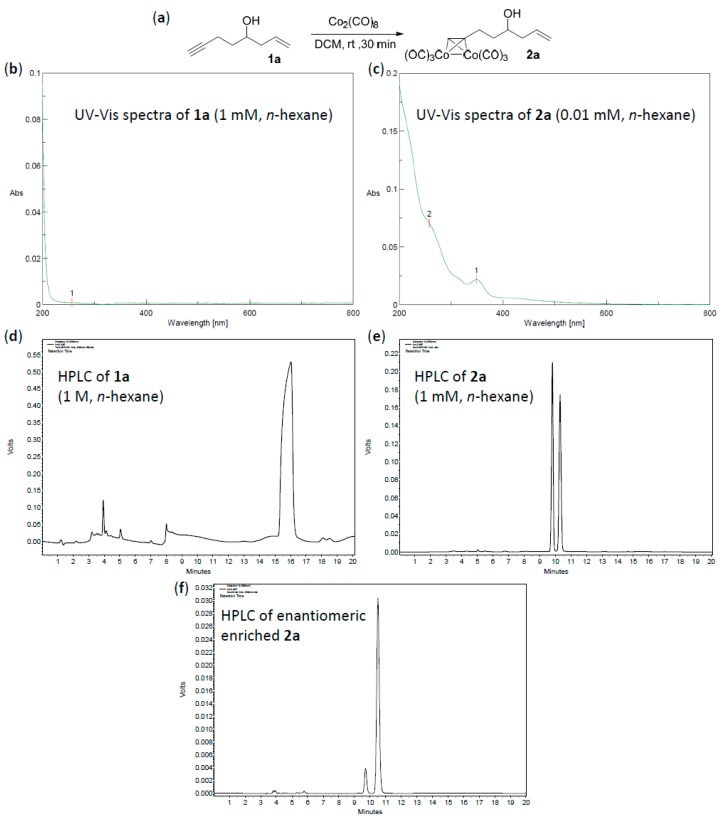
Preparation of **2a** and the comparison of its properties with those of **1a**. (**a**) Transformation from **1a** to **2a**; (**b**) The UV-Vis spectra of 1 mM solution of **1a** in *n*-hexane; (**c**) The UV-Vis spectra of 0.01 mM solution of **2a** in *n*-hexane; (**d**) The high-performance liquid chromatography (HPLC) chromatogram of injected **1a** (20 µL, 1 M in *n*-hexane), with CHIRALPAK-IB column, 2-PrOH/*n*-hexane 0.4:99.6 eluting-solvent system, 1 mL·min^−1^ flow rate, and 200 nm detection wavelength at 25 °C; (**e**) The HPLC chromatogram of racemic **2a** (20 µL, 1 mM in *n*-hexane) with the same conditions as (**d**) except the detection wavelength of 350 nm; (**f**) The HPLC chart of enantioenriched **2a** with the same conditions as (**e**).

Enantiomeric purity is vital for chiral alkynes to function. The enantiomeric excess (ee) of chiral alkynes with unknown optical rotation could be determined generally in two ways: (1) using another chiral reagent to derivate diastereomers and then measure the diasteromeric ratio; (2) using enantioselective chromatography to directly measure the enantiomeric excess. Recently, in the course of our total synthesis of natural products, we prepared the known alkyne **1a** (Figure 1), and found that two methods were used in the literatures to determine its ee [5,6,7,8,9,10,11,12,13]. Most literature used Mosher’s methodology [14] which undertook an esterification with expensive Mosher’s chiral reagent, and determined the enantiomeric purity by NMR analysis of the resultant diasteromeric mixture [5,6,7,8,9,10,11,12]. One study used high-performance liquid chromatography (HPLC) with chiral column to analyze the 3,5-dinitrobenzoate derivative of **1a** [13]. Since both methods need a derivatization reaction with tedious workup procedure, we initially attempted a direct analysis of **1a** with chiral column HPLC method. However, no good result was achieved, which may be due to two causes: (1) the alkyne- and alkene-chains in **1a** are similar in size, and therefore might be difficult to be differentiated by chiral stationary phase. In fact, no enantiomeric separation could be found in all our attempted chiral columns and HPLC conditions; (2) alkyne and other functional groups in **1a** are all poor chromophores. As a result, **1a** showed very low UV-absorption, and it was difficult to characterize under normal HPLC concentration using a UV detector.

Aware of the insufficiency of the present ee determination methodology for chiral alkynes such as **1a**, we decided to develop and report herein a simple and general method by analyzing the in situ generated cobalt-complex of chiral alkynes using chiral column HPLC. This new method is especially suitable for the alkynes without chromophores and other derivable groups.

## 2. Results and Discussion

To overcome the above-mentioned two weak points of alkynes, our idea was to make use of its metal complex. It is well known that Co_2_(CO)_8_ can easily react with alkynes to lose two carbon monoxides and form stable Co_2_(CO)_6_–alkyne complexes. Figure 1a showed this transformation from alkyne **1a** to its complex **2a**. By this simple complexation, (1) the alkyne-chain became significantly bulky, which was very different in size from the other side-chain; (2) the Co-complexation substructure was a good chromophore, making the UV absorption of **2a** more than 100 times stronger than **1a** (Figure 1b,c), and a wide range of detection wavelength (200–400 nm) could be selected in the HPLC experiment. As a result, comparison with the unsuccessful result of **1a** (Figure 1d), **2a** was easily resolved by HPLC using a CHIRALPAK-IB column with 0.4:99.6 2-PrOH/*n*-hexane eluting-solvent system, 1 mL·min^−1^ flow rate, and 350 nm detection wavelength at 25 °C (Figure 1e). Under the same conditions, an enantioenriched **2a** was also tested (Figure 1f), and the detected enantiomeric ratio was consistent with that determined by the modified Mosher’s methodology [11].

After confirmation of our idea on compounds **1a** and **2a**, we next prepared a series of Co_2_(CO)_6_–alkyne complexes **2b**–**k** (Table 1) from none-chromophore alkynes, and checked their resolution by HPLC chiral column. As a result, all the enantiomeric pairs of Co-complexes were successfully baseline-separated. For alcohol substrates, the size of another side-chain (butyl, allyl, ethyl, methyl) or the distance between the C≡C triple bond and the hydroxyl group did not show any notable effect for the enantiomeric separation (**2a**–**e**). Even a tertiary alkynol-complex **2f** could be well resolved, although a CHIRALPAK-IA column was used in this case. Internal alkyne-complexes **2g** and **2h** also gave good separation. Replacing the hydroxyl group with halogen atoms might change the interaction between the substrates and the stationary phase of the column. Fortunately, the chloride **2i** and the fluoride **2j** were both resolved, although lower polar eluting-solvent systems were needed for the low polarity of these substrates. Notably, the tetrahydropyran (THP)-protected alkynol-complex **2k** could also been enantiomerically separated. Actually, compounds such as **1i**–**k**, are not good substrates for classical pre-column derivatization methods.

It was noted that most of the above HPLC experiments were carried out with the same chiral column using the most general 2-PrOH/*n*-hexane eluting-solvent system. Except for changing the 2-PrOH/*n*-hexane ratio to make the retention time between 10–20 min, no more optimization was done for HPLC conditions. The simple setup of the HPLC conditions implied the easiness and generality of our Co-complex analytic method on much wider alkyne substrates.

**Figure 2:**
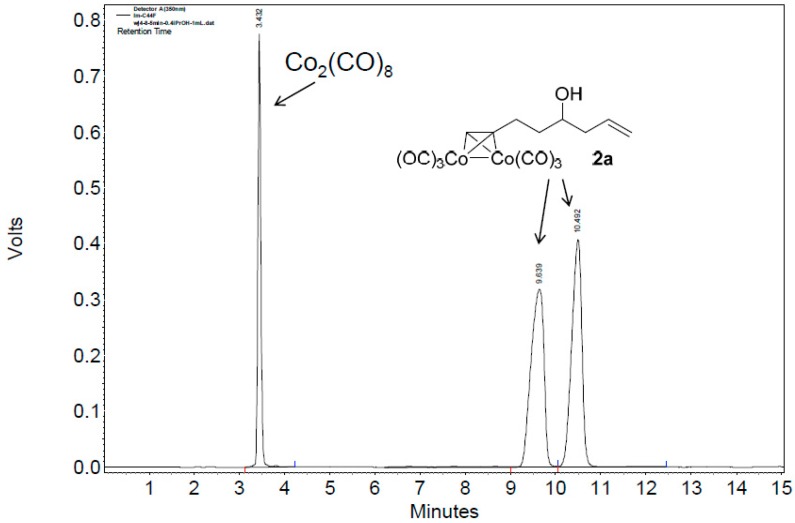
The HPLC chromatogram of the reaction mixture of **1a**, Co_2_(CO)_8_, and CH_2_Cl_2_, with CHIRALPAK-IB column, 2-PrOH/*n*-hexane 0.4:99.6, 1 mL·min^−1^ flow rate, and 350 nm detection wavelength at 25 °C. Sample preparation: 20 µL of the reaction mixture was taken 5 min after the reaction started, diluted with 0.5 mL *n*-hexane, and passed through a disposable syringe filter (Nylon 66, 0.22 µm, 13 mm); 5 µL of the filtration was injected.

Although the above HPLC analysis was all carried out with purified Co-complexes, since the CHIRALPAK immobilized-polysaccharide type columns can tolerate a wide range of solvents, we next attempted to directly measure the reaction system of **1a**, Co_2_(CO)_8_, and CH_2_Cl_2_ (Figure 1a). To our delight, by just diluting a small amount of the reaction mixture with *n*-hexane and then injection into HPLC, the in situ generated **2a** could be monitored without any problems (Figure 2). Since the manipulation was rather simple without the tedious workup procedure like those of Mosher’s or other carbonyl chloride methods [5,6,7,8,9,10,11,12,13], this result implied a promising application of our Co-complexation method in monitoring the asymmetric alkyne-synthesis reactions.

## 3. Materials and Methods 

### 3.1. General Methods

All reactions were carried out under an atmosphere of Ar unless otherwise indicated. ^1^H-, and ^13^C-NMR spectra were acquired on Mercury-300 (Agilent, Santa Clara, CA, USA), AVANCE III-400 (Bruker, Billerica, MA, USA), WNMR-I-500 (Zhongke Niujin Co., Ltd., Wuhan, China), or VNMRS-600 spectrometers (Agilent). Chemical shifts are indicated in parts per million (ppm) downfield from tetramethylsilane (TMS, δ = 0.00) with residual undeuterated solvent peaks as internal reference for ^1^H-NMR and deuterated solvent peaks shifts for ^13^C-NMR. Multiplicities are reported as s (singlet), d (doublet), t (triplet), q (quartet), m (multiplet), br (broad) or combinations of those. For NMR analysis of the Co_2_(CO)_6_-alkyne complexes, the sample solution in CDCl_3_ should pass through a disposable syringe filter (Nylon 66, 0.22 µm, 13 mm) immediately before the NMR experiment, to remove the small amount of paramagnetic material. Mass spectra (MS) are electron ionization (EI) or electrospray ionization (ESI). EI-MS data were measured on GCT mass spectrometer (Micromass, Manchester, UK). ESI-MS data were measured on Thermo-Fisher Accela liquid chromatography system coupled with Exactive Plus Orbitrap mass spectrometer (Thermo-Fisher, Bremen, Germany). Reagents and compounds **1c**–**f**, **1k** were purchased from commercial suppliers and used as received. Compound **1a** was prepared according to the literature procedure [8]. Compounds **1b**, **1g**–**j**, and **2a**–**k** were synthesized as following. See Supplementary Materials for spectra and chromatograms of the prepared products. 

### 3.2. Preparation of Alkynes and Compound Characterization

*Non-1-yn-5-ol* (**1b**). To dry THF (254 mL) was added *n*-BuLi (2.5 M in *n*-hexane, 9.74 mL, 24.4 mmol) at −78 °C. 4-pentyn-1-al [8] (1.00 g, 12.2 mmol) was added slowly at −78 °C. Then the mixture was stirred for 3 h while its temperature reached 23 °C. Methanol (50 mL) was added to quench excess *n*-BuLi. The mixture was concentrated. The residue was dissolved in 10% aqueous HCl and the resulting mixture was extracted with CH_2_Cl_2_. The combined organic layers were dried over Na_2_SO_4_, concentrated, and purified by silica gel column chromatography. Elution with cyclohexane/acetone (50/1) gave a pale yellow oil (0.38 g, 22%); ^1^H-NMR (300 MHz, CDCl_3_) δ 3.75 (br, 1H, C*H*OH), 2.34 (t, *J* = 6.0 Hz, 2H), 1.98 (s, 1H, ≡C*H*), 1.72–1.60 (m, 2H), 1.67 (br, 1H, O*H*), 1.46–1.33 (m, 6H), 0.91 (t, *J* = 6.3 Hz, 3H, C*H_3_*). ^13^C-NMR (75 MHz, CDCl_3_) δ 84.5 (*C*≡CH), 71.0, 68.9, 37.3, 35.9, 28.0, 22.9, 15.2, 14.3. HRMS (ESI) *m*/*z* calcd. for C_9_H_17_O^+^ [M + H]^+^: 141.1274; found: 141.1269.

*Dec-1-en-7-yn-4-ol* (**1g**). To a solution of *tert*-butyldimethyl(oct-1-en-7-yn-4-yloxy)silane [8] (3.00 g, 12.6 mmol) in dry THF (15 mL) cooled at −78 °C was added dropwise lithium diisopropylamide (LDA, 10.064 mL, 2.5 M in THF, 25.16 mmol) via syringe. The resulting mixture was stirred at −78 °C for 1 h followed by addition of iodoethane (5.056 mol, 62.9 mmol). The reaction solution allowed to warm to room temperature. After being stirred for 26 h, the mixture was passed through a silica-gel pad (eluted with CH_2_Cl_2_), and concentrated in vacuum. To the residue was added tetrabutylammonium fluoride (1 M in THF, 39.4 mL, 39.4 mmol) at room temperature. The reaction mixture was stirred for 10 h, and then was quenched with saturated aqueous NH_4_Cl. The aqueous layer was extracted with CH_2_Cl_2_. The organic phase was then dried over Na_2_SO_4_, concentrated, and purified by silica gel column chromatography. Elution with cyclohexane/CH_2_Cl_2_ (30/1) gave a pale yellow oil (0.98 g, 51%); ^1^H-NMR (500 MHz, CDCl_3_) δ 5.82 (m, 1H, C*H*=*C*H_2_), 5.14–5.11 (m, 2H, CH=*CH*_2_), 3.80 (m, 1H, C*H–*OH), 2.32–2.26 (m, 3H), 2.21–2.11 (m, 3H), 2.00 (br, 1H, O*H*), 1.66 (m, 1H), 1.60 (m, 1H), 1.10 (t, *J* = 7.5 Hz, 3H, C*H_3_*). ^13^C-NMR (125 MHz, CDCl_3_) δ 134.6 (*C*H=CH_2_), 118.0 (CH=*C*H_2_), 82.4, 78.8, 70.0 (*C*H–OH), 41.8, 35.6, 15.3, 14.2, 12.3. HRMS (ESI) *m*/*z* calcd. for C_10_H_17_O^+^ [M + H]^+^: 153.1274; found: 153.1269.

*8-(Trimethylsilyl)oct-1-en-7-yn-4-ol* (**1h**). To a solution of 5-(trimethylsilyl)pent-4-ynal [15] (9.17 g, 59.4 mmol) in CH_2_Cl_2_ (400 mL) cooled at −78 °C was added dropwise allyboronic acid pinacol ester (12.26 mL, 65.4 mmol). The resulting mixture was stirred at −78 °C for 1 h, and then at 0 °C for 3 h. The reaction was quenched by addition of water, extracted with CH_2_Cl_2_, and washed with brine. The organic layer was dried with Na_2_SO_4_ and concentrated. To the residue was added CH_2_Cl_2_ (100 mL) and triethanolamine (15 mL, 112 mmol) at room temperature. The mixture was stirred for 3 h, then passed through a silica gel pad (eluted with CH_2_Cl_2_), concentrated, and purified by silica gel column chromatography. Elution with cyclohexane/EtOAc (100/1) gave a yellow oil (11.13 g, 95%); ^1^H-NMR (400 MHz, CDCl_3_) δ 5.82 (m, 1H, C*H*=*C*H_2_), 5.17–5.12 (m, 2H, CH=*CH*_2_), 3.79 (m, 1H, C*H–*OH), 2.38 (t, *J* = 7.2 Hz, 2H), 2.30 (m, 1H), 2.20 (m, 1H), 1.91 (br, 1H, O*H*), 1.74–1.61 (m, 2H), 0.14 (s, 9H, C*H_3_* × 3). ^13^C-NMR (100 MHz, CDCl_3_) δ 134.5 (*C*H=CH_2_), 118.2 (CH=*C*H_2_), 106.9 (≡*C*–Si), 85.3 (*C*≡C–Si), 70.0 (*C*H–OH), 41.8, 35.2, 16.5, 0.08 (Si*C*H*_3_*× 3). HRMS (ESI) *m*/*z* calcd. for C_11_H_21_OSi^+^ [M + H]^+^: 197.1356; found: 197.1357.

*4-Chlorooct-1-en-7-yne* (**1i**). To a solution of oct-1-en-7-yn-4-ol (**1a**, 300 mg, 2.4 mmol) in CH_2_Cl_2_ (18 mL) at 0 °C, pyridine (0.384 mL, 4.8 mmol) was then added, followed by triphosgene (356 mg, 1.2 mmol) in one portion. The solution was stirred for 5 min and then warmed to gentle reflux. After 6 h, the reaction mixture was poured into a separatory funnel containing 1 M aqueous HCl (20 mL), and the biphasic mixture was shaken vigorously. Upon separation of layers, the aqueous layer was re-extracted with CH_2_Cl_2_. The organic phase was dried over Na_2_SO_4_, concentrated, and purified by silica gel column chromatography. Elution with cyclohexane gave a pale yellow oil (213.6 mg, 62%); ^1^H-NMR (300 MHz, CDCl_3_) δ5.85 (m, 1H, C*H*=*C*H_2_), 5.18–5.13 (m, 2H, CH=*CH*_2_), 4.10 (m, 1H, C*H*–Cl), 2.56 (m, 2H), 2.41 (m, 2H), 2.05-1.82 (m, 2H), 1.98 (s, 1H, ≡C*H*). ^13^C-NMR (75 MHz, CDCl_3_) δ 133.7 (*C*H=CH_2_), 118.2 (CH=*C*H_2_), 82.8 (*C*≡CH), 69.1 (C≡*C*H), 60.8 (*C*H–Cl), 42.5, 36.2, 15.8. HRMS (ESI) *m*/*z* calcd. for C_8_H_12_^35^Cl^+^ [M + H]^+^: 143.0622; found: 143.0618.

*4-Fluorodec-1-en-7-yne* (**1j**). Dec-1-en-7-yn-4-ol (1g, 300 mg, 1.96 mmol) was added to a previously cooled (−45 °C) solution of diethylaminosulfurtrifluoride (594 mg, 3.69 mmol) in dry CH_2_Cl_2_ (2.84 mL) with vigorous stirring over a 10-min period. The solution was allowed to come to room temperature overnight after which time it was transferred into a separatory funnel containing water and CH_2_Cl_2_. The organic phase was then dried over Na_2_SO_4_, concentrated, and purified by silica gel chromatography. Elution with cyclohexane/CH_2_Cl_2_ (50/1) gave a yellow oil (15 mg, 5.0%); ^1^H-NMR (300 MHz, CDCl_3_) δ 5.82 (m, 1H, C*H*=*C*H_2_), 5.16–5.10 (m, 2H, CH=*CH*_2_), 4.66 (brd, *J* = 51.3 Hz, 1H, C*H-*F), 2.43–2.29 (m, 4H), 2.15 (q, *J* = 6.9 Hz, 2H, C*H_2_*CH*_3_*), 1.85–1.69 (m, 2H), 1.11 (t, *J* = 7.2 Hz, 3H, C*H_3_*). ^13^C-NMR (75 MHz, CDCl_3_) δ 133.0 (d, *J* = 6.0 Hz, *C*H=CH_2_), 117.9 (CH=*C*H_2_), 92.0 (d, *J* = 168.9 Hz, *C*H–F), 82.3, 78.1, 39.3 (d, *J* = 21.2 Hz), 34.0 (d, *J* = 20.9 Hz), 14.7 (d, *J* = 5.2 Hz), 14.2, 12.4. LRMS (ESI) *m*/*z* calcd. for C_10_H_16_F^+^ [M + H]^+^: 155.1; found: 155.1.

### 3.3. General Precedure for Preparation of Co_2_(CO)_6_–Alkyne Complexes and Compound Characterization

A mixture of alkyne **1** (2 mmol, 1.0 equiv.), Co_2_(CO)_8_ (2.2 mmol, 1.1 equiv.) in CH_2_Cl_2_ (0.5 mL) was stirred at room temperature under air atmosphere (balloon) until thin-layer chromatography (TLC) monitoring showed all alkyne consumed (about 1 h). The solvent was removed under reduced pressure, and the residue was purified by silica gel column chromatography to provide Co_2_(CO)_6_–alkyne complex **2**.

*Hexacarbonyl[µ-[(7,8-η:7,8-η)oct-1-en-7-yn-4-ol]]-dicobalt-(Co–Co)* (**2a**): Dark red oil, yield 57%; ^1^H-NMR (500 MHz, CDCl_3_) δ 6.02 (s, 1H, ≡C*H*), 5.83 (m, 1H, C*H*=*C*H_2_), 5.19–5.15 (m, 2H, CH=*CH*_2_), 3.79 (m, 1H, C*H*-OH), 3.09 (m, 1H), 2.92 (m, 1H), 2.36 (m, 1H), 2.22 (m, 1H), 1.80 (m, 2H), 1.66 (d, *J* = 4.0 Hz, 1H, O*H*). ^13^C-NMR (125 MHz, CDCl_3_) δ 199.9 (br, *C*O × 6), 134.2 (*C*=CH), 118.8 (CH=*C*H_2_), 97.1 (*C*≡CH), 73.1 (C≡*C*H), 69.9 (*C*HOH), 42.1, 38.9, 30.4. HRMS (EI) *m*/*z* calcd. for C_13_H_162_Co_2_O_6_^+^ [M^+^ − CO]: 381.9292; found: 381.9300.

*Hexacarbonyl[µ-[(1,2-η:1,2-η)non-1-yn-5-ol]]-dicobalt-(Co–Co)* (**2b**): Dark red oil, yield 58%; ^1^H-NMR (300 MHz, CDCl_3_) δ 6.01 (s, 1H, ≡C*H*), 3.92 (m, 1H, C*H*OH), 3.07 (ddd, *J* = 15.6, 9.6, 6.3 Hz, 1H), 2.89 (ddd, *J* = 15.6, 9.9, 6.3 Hz, 1H), 1.76 (m, 2H), 1.49–1.43 (m, 4H), 1.38-1.33 (m, 3H), 0.92 (t, *J* = 6.6 Hz, 3H, C*H_3_*). ^13^C-NMR (75 MHz, CDCl_3_) δ 200.0 (br, *C*O × 6), 97.3 (*C*≡CH), 73.0 (C≡*C*H), 71.3 (*C*HOH), 39.5, 37.2, 30.3, 27.7, 22.7, 14.0 (*C*H_3_). HRMS (EI) *m*/*z* calcd. for C_14_H_16_Co_2_O_6_^+^ [M^+^ − CO]: 397.9605; found: 397.9615.

*Hexacarbonyl[µ-[(4,5-η:4,5-η)pent-4-yn-2-ol]]-dicobalt-(Co–Co)* (**2c**): Dark red oil, yield 67%; ^1^H-NMR (300 MHz, CDCl_3_) δ 6.10 (s, 1H, ≡C*H*), 3.95 (m, 1H, C*H*OH), 3.01 (d, *J* = 4.8 Hz, 2H, C*H*_2_), 1.57 (d, *J* = 6.3 Hz, 1H, O*H*), 1.35 (d, *J* = 5.4 Hz, 3H, C*H_3_*). ^13^C-NMR (75 MHz, CDCl_3_) δ 199.8 (br, *C*O × 6), 92.0 (*C*≡CH), 73.9 (C≡*C*H), 68.7 (*C*HOH), 43.7 (*C*H_2_), 23.7 (*C*H_3_). HRMS (EI) *m*/*z* calcd. for C_10_H_8_Co_2_O_6_^+^ [M^+^ − CO]: 341.8979; found: 341.8987. 

*Hexacarbonyl[µ-[(5,6-η:5,6-η)hex-5-yn-3-ol]]-dicobalt-(Co–Co)* (**2d**): Dark red oil, yield 78%; ^1^H-NMR (300 MHz, CDCl_3_) δ 6.09 (s, 1H, ≡C*H*), 3.66 (m, 1H, C*H*OH), 3.00 (m, 2H, ≡CC*H*_2_), 1.76 (d, *J* = 3.3 Hz, 1H, O*H*), 1.63 (m, 2H, C*H_2_*CH*_3_*), 1.01 (t, *J* = 7.2 Hz, 3H, C*H_3_*). ^13^C-NMR (75 MHz, CDCl_3_) δ 199.8 (br, *C*O × 6), 92.5 (*C*≡CH), 74.1 (C≡*C*H), 73.9 (*C*HOH), 41.5 (≡C*C*H_2_), 30.2 (*C*H_2_CH_3_), 9.7 (*C*H_3_). HRMS (EI) *m*/*z* calcd. for C_11_H_10_Co_2_O_6_^+^ [M^+^ − CO]: 355.9136; found: 355.9142.

*Hexacarbonyl[µ-[(1,2-η:1,2-η)pent-1-yn-3-ol]]-dicobalt-(Co–Co)* (**2e**): Dark red oil, yield 84%; ^1^H-NMR (600 MHz, CDCl_3_) δ 6.06 (d, *J* = 0.6 Hz, 1H, ≡C*H*), 4.63 (ddd, *J* = 7.8, 5.4, 5.4 Hz, 1H, C*H*OH), 1.84 (d, *J* = 5.4 Hz, 1H, O*H*), 1.76 (m, 1H of C*H_2_*), 1.70 (m, 1H of C*H_2_*), 1.09 (t, *J* = 7.2 Hz, 3H, C*H_3_*). ^13^C-NMR (150 MHz, CDCl_3_) δ 199.6 (br, *C*O × 6), 99.8 (*C*≡CH), 73.9, 71.6, 32.9 (*C*H_2_), 10.6 (*C*H_3_). HRMS (EI) *m*/*z* calcd. for C_10_H_8_Co_2_O_6_^+^ [M^+^]: 369.8929; found: 369.8933.

*Hexacarbonyl[µ-[(1,2-η:1,2-η)-3-methylpent-1-yn-3-ol]]-dicobalt-(Co–Co)* (**2f**): Dark red oil, yield 78%; ^1^H-NMR (300 MHz, CDCl_3_) δ 6.06 (s, 1H, ≡C*H*), 1.78 (m, 2H, C*H*_2_), 1.73 (s, 1H, O*H*), 1.49 (s, 3H, C*H*_3_), 1.03 (t, *J* = 7.2 Hz, 3H, CH_2_C*H*_3_). ^13^C-NMR (75 MHz, CDCl_3_) δ 199.6 (br, *C*O × 6), 105.2 (*C*≡CH), 74.9, 72.1, 37.8 (*C*H_2_), 29.8 (*C*H_3_), 8.7 (CH_2_*C*H_3_). HRMS (EI) *m*/*z* calcd. for C_12_H_10_Co_2_O_7_^+^ [M^+^]: 383.9085; found: 383.9090.

*Hexacarbonyl[µ-[(7,8-η:7,8-η)dec-1-en-7-yn-4-ol]]-dicobalt-(Co–Co)* (**2g**): Dark red oil, yield 69%; ^1^H-NMR (300 MHz, CDCl_3_) δ 5.82 (m, 1H, C*H*=*C*H_2_), 5.20-5.15 (m, 2H, CH=*CH*_2_), 3.79 (brd, *J* = 3.9 Hz, 1H, C*H-*OH), 3.08 (m, 1H), 2.91 (m, 1H), 2.86 (q, *J* = 7.2 Hz, 2H, C*H*_2_CH*_3_*), 2.36 (m, 1H), 2.22 (m, 1H), 1.82 (m, 2H), 1.69 (d, *J* = 3.9 Hz, 1H, O*H*), 1.29 (t, *J* = 7.2 Hz, 3H, C*H_3_*). ^13^C-NMR (75 MHz, CDCl_3_) δ 200.3 (br, *C*O × 6), 134.2 (*C*H=CH_2_), 118.8 (CH=*C*H_2_), 101.8, 99.0, 70.0 (*C*H-OH), 42.0, 38.3, 30.1, 27.0, 15.6 (*C*H*_3_*). HRMS (EI) *m*/*z* calcd. for C_15_H_16_Co_2_O_6_^+^ [M^+^ − CO]: 409.9605; found: 409.9607.

*Hexacarbonyl[µ-[(7,8-η:7,8-η)-8-(trimethylsilyl)oct-1-en-7-yn-4-ol]]-dicobalt-(Co–Co)* (**2h**): Dark red oil, yield 49%; ^1^H-NMR (300 MHz, CDCl_3_) δ 5.82 (m, 1H, C*H*=*C*H_2_), 5.21-5.16 (m, 2H, CH=*CH*_2_), 3.80 (m, 1H, C*H-*OH), 3.20 (m, 1H), 2.96 (m, 1H), 2.40 (m, 1H), 2.23 (m, 1H), 1.82 (m, 2H), 1.68 (s, 1H, O*H*), 0.30 (s, 9H, C*H_3_* × 3). ^13^C-NMR (75 MHz, CDCl_3_) δ 200.6 (br, *C*O × 6), 134.1 (*C*H=CH_2_), 118.8 (CH=*C*H_2_), 112.3 (≡*C*-Si), 79.1 (*C*≡C-Si), 70.0 (*C*H-OH), 42.0, 39.2, 31.4, 0.65 (Si*C*H*_3_*× 3). HRMS (EI) *m*/*z* calcd. for C_16_H_20_Co_2_O_6_Si^+^ [M^+^ − CO]: 453.9688; found: 453.9693.

*Hexacarbonyl[µ-[(7,8-η:7,8-η)-4-chlorooct-1-en-7-yne]]-dicobalt-(Co–Co)* (**2i**): Dark red oil, yield 74%; ^1^H-NMR (300 MHz, CDCl_3_) δ 6.03 (s, 1H, ≡C*H*), 5.85 (m, 1H, C*H*=*C*H_2_), 5.18–5.13 (m, 2H, CH=*CH*_2_), 4.05 (m, 1H, C*H-*Cl), 3.11 (m, 1H), 2.97 (m, 1H), 2.57 (m, 2H), 2.05 (m, 2H). ^13^C-NMR (75 MHz, CDCl_3_) δ 199.9 (br, *C*O × 6), 133.6 (*C*H=CH_2_), 118.4 (CH=*C*H_2_), 95.7 (*C*≡CH), 73.1 (C≡*C*H), 61.2 (*C*H-Cl), 42.7, 39.5, 30.8. HRMS (EI) *m*/*z* calcd. for C_13_H_11_Co_2_^35^ClO_5_^+^ [M^+^ − CO]: 399.8954; found: 399.8960.

*Hexacarbonyl[µ-[(7,8-η:7,8-η)-4-fluorodec-1-en-7-yne]]-dicobalt-(Co–Co)* (**2j**): Dark red oil, yield 90%; ^1^H-NMR (600 MHz, CDCl_3_) δ 5.84 (ddt, *J* = 24.6, 17.4, 7.2 Hz, 1H, C*H*=*C*H_2_), 5.17–5.13 (m, 2H, CH=*CH*_2_), 4.66 (brd, *J* = 48.6 Hz, 1H, C*H-*F), 3.06 (m, 1H), 2.91 (m, 1H), 2.86 (q, *J* = 7.2 Hz, 2H, C*H_2_*CH*_3_*), 2.46 (m, 2H), 1.93 (m, 2H), 1.29 (t, *J* = 7.2 Hz, 3H, C*H_3_*). ^13^C-NMR (150 MHz, CDCl_3_) δ 200.1 (br, *C*O × 6), 132.6 (d, *J* = 6.3 Hz, *C*H=CH_2_), 118.3 (CH=*C*H_2_), 101.8 (*C*≡CEt), 98.2 (C≡*C*Et), 92.5 (d, *J* = 170.1 Hz, *C*H-F), 39.4 (d, *J* = 21.3 Hz, *C*H_2_CH=CH_2_), 36.2 (d, *J* = 20.7 Hz, *C*H_2_CH_2_C≡C), 29.3 (d, *J* = 4.1 Hz, C≡C*C*H_2_CH_2_), 27.0 (*C*H_2_CH_3_), 15.6 (*C*H_3_). HRMS (EI) *m*/*z* calcd. for C_15_H_15_Co_2_FO_5_^+^ [M^+^ − CO]: 411.9562; found: 411.9570.

*Hexacarbonyl[µ*-[2-((3,4-η:3,4-η)but-3-yn-1-yloxy)tetrahydro-2H-pyran]*]-dicobalt-(Co–Co)* (**2k**): Dark red oil, yield 65%; ^1^H-NMR (500 MHz, CDCl_3_) δ 6.03 (s, 1H, ≡C*H*), 4.63 (t, *J* = 4.0 Hz, 1H, O–C*H*–O), 3.99 (m, 1H), 3.88 (m, 1H), 3.60 (m, 1H), 3.53 (m, 1H), 3.15 (m, 2H), 1.84 (m, 1H), 1.72 (m, 1H), 1.62–1.51 (m, 4H). ^13^C-NMR (125 MHz, CDCl_3_) δ 199.9 (br, *C*O × 6), 98.9 (O–*C*H–O), 92.9 (*C*≡CH), 73.7 (C≡*C*H), 67.3, 62.4, 34.1, 30.6, 25.4, 19.5. HRMS (EI) *m*/*z* calcd. for C_14_H_14_Co_2_O_7_^+^ [M^+^ − CO]: 411.9398; found: 411.9400.

## 4. Conclusions

We have developed a simple and general method to separate the enantiomers of chiral alkynes using their in situ generated cobalt-complex by chiral column HPLC. HPLC analysis of enantiomeric purity of metal complexes with carbon-metal bonds is not usual [16]. Our method is particularly useful for the alkynes without chromophores and other derivable groups. Since the decomplexation of Co-alkyne complexes is well known [17], our method is also promisingly suitable for the preparative resolution of racemic alkynes.

## Figures and Tables

**Table 1:** Scope of alkynes for HPLC resolution of their Co-complexes.

Alkyne (1)	Co-Complex (2)	HPLC Chart	HPLC Conditions (CHIRALPAK–IB Column 350 nm, 25 °C)
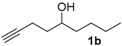	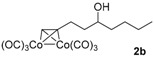	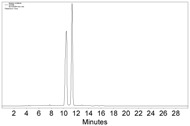	2-PrOH/*n*-hexane = 0.4:99.6 1 mL·min^−1^
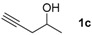	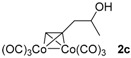	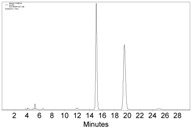	2-PrOH/*n*-hexane = 0.4:99.6 1 mL·min^−1^
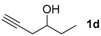	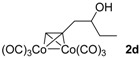	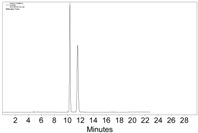	2-PrOH/*n*-hexane = 0.4:99.6 1 mL·min^−1^
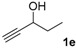	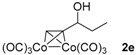	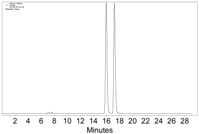	2-PrOH/*n*-hexane = 0.4:99.6 1 mL·min^−1^
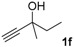	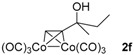	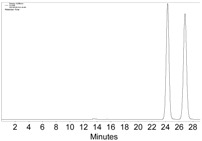	2-PrOH/*n*-hexane = 0.4:99.6 1 mL·min^−1^ ^a^
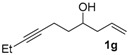	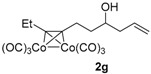	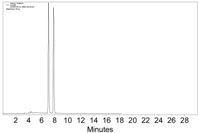	2-PrOH/*n*-hexane = 0.5:99.5 1 mL·min^−1^
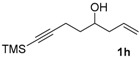	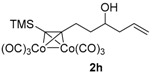	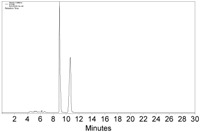	2-PrOH/*n*-hexane = 0.3:99.7 1 mL·min^−1^
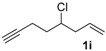	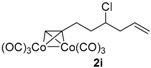	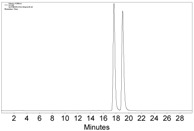	EtOAc/*n*-hexane = 0.05:99.95 0.3 mL·min^−1^ ^b^
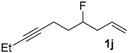	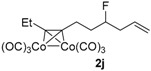	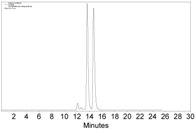	CH_2_Cl_2_/*n*-hexane = 0.05:99.95 0.4 mL·min^−1 c^
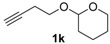	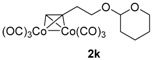	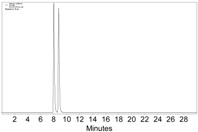	2-PrOH/*n*-hexane = 0.1:99.9 1 mL·min^−1^

**^a^** CHIRALPAK-IA column was used; ^b^ The HPLC was measured at 0 °C; ^c^ The HPLC was measured at 10 °C.

## Supplementary Materials

Supplementary materials are available online. (1) General Information; (2) UV-Vis spectra of **1a** and **2a**; (3) ^1^H- and ^13^C-NMR spectra of **1b**, and **1g**–**j**; (4) HPLC chart of **1a**; (5) ^1^H-, ^13^C-NMR spectra and HPLC charts of **2a**–**k**; (6) HPLC monitor of the reaction of **1a** and Co_2_(CO)_8_.
