# Supplementary material for: Separation of Alkyne Enantiomers by Chiral Column HPLC Analysis of Their Cobalt-Complexes
Source: Molecules. 2017 Mar 20;22(3):466. doi: 10.3390/molecules22030466 (PMC6155393; doi:10.3390/molecules22030466)
Supplement: Supplementary file 1 [file molecules-22-00466-s001.pdf]

Separation of Alkyne Enantiomers by Chiral Column HPLC Analysis of Their Cobalt-Complexes

Qiaoyun Liu [1], Jing Wang [1,2], Junfei Li [1], Xiaolei Wang [2], Shichao Lu [2], Xuan Li [2], Yaling Gong [2,*] and Shu Xu [2,*]

[1] School of Chemistry and Material Science, Shanxi Normal University, 1 Gongyuan Street, Linfen, Shanxi Province 041004, P.R. China
[2] State Key Laboratory of Bioactive Substance and Function of Natural Medicines, Beijing Key Laboratory of Active Substances Discovery and Drugability Evaluation, Institute of Materia Medica, Chinese Academy of Medical Sciences and Peking Union Medical College, 2A NanWei Road, Xicheng Distrct, Beijing 100050, P.R. China

Table of Contents

1.	General Information	S2
2.	UV-Vis spectra of **1a** and **2a**	S3-S4
3.	^{1}H- and ^{13}C-NMR spectra of **1b**, and **1g-j**	S5-S14
4.	HPLC chart of **1a**	S15
5.	^{1}H-, ^{13}C-NMR spectra, and HPLC charts of **2a-k**	S16-S50
6.	HPLC monitor of the reaction of **1a** and $Co_2(CO)_8$	S51-S54
7.		
8.		
9.		

1. General Information

Reagents were used as received from commercial suppliers unless otherwise indicated. All reactions were carried out under an atmosphere of Ar unless otherwise indicated. Column chromatography was performed with silica gel 60 (160-200 mesh, Qingdao Haiyang Chemical Factory). Analytical thin-layer chromatography (TLC) was performed with glass TLC plates (Qingdao silica gel GF-F_{254} plates). Visualization was accomplished with UV light, or potassium permanganate staining, or phosphomolybdic acid staining and subsequent heating.

UV-Vis spectra were acquired on JASCO-V750 Spectrophotometer. HPLC analysis were acquired on SHIMAZU SCL-10Avp. The CHIRALPAK-IB column is analytical type with the packing composition of cellulose tris(3,5-dimethylphenylcarbamate), internal diameter of 4.6 mm, column length of 250 mm, and particle size of 5μm. The CHIRALPAK-IA column is analytical type with the packing composition of amylose tris(3,5-dimethylphenylcarbamate), internal diameter of 4.6 mm, column length of 250 mm, and particle size of 5μm. ^{1}H-, and ^{13}C-NMR spectra were acquired on Mercury-300, or Bruker AVANCE III-400, or WNMR-I-500, or VNMRS-600 spectrometers. Chemical shifts are indicated in parts per million (ppm) downfield from tetramethylsilane (TMS, $\delta = 0.00$) with residual undeuterated solvent peaks as internal reference for ^{1}H-NMR and deuterated solvent peaks shifts for ^{13}C-NMR. Multiplicities are reported as s (singlet), d (doublet), t (triplet), q (quartet), m (multiplet), br (broad) or combinations of those. For NMR analysis of the $Co_2(CO)_6$–alkyne complexes, the sample solution in $CDCl_3$ should pass through a disposable syringe filter (Nylon 66, 0.22 μm, 13 mm) immediately before the NMR experiment, to remove the tiny amount of paramagnetic material. Mass spectra are electron ionization (EI) or electrospray ionization (ESI). EI-MS data were measured on Micromass GCT mass spectrometer. ESI-MS data were measured on Thermo Exactive Orbitrap plus spectrometer.

2. UV-Vis spectra of **1a** and **2a**

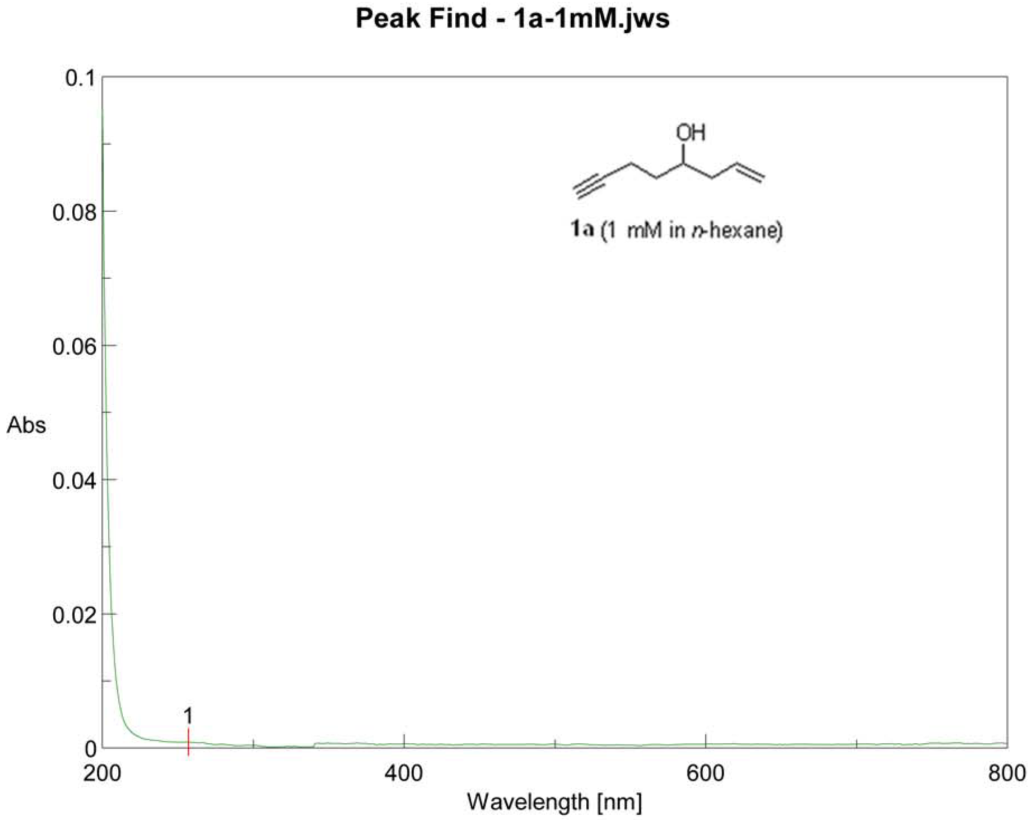

[Detailed Information]

Creation date	2017/1/17 9:25
Data array type	Linear data array
Horizontal axis	Wavelength [nm]
Vertical axis	Abs
Start	800 nm
End	200 nm
Data interval	1 nm
Data points	601

[Measurement Information]

Instrument name	V-750
Model name	V-750
Serial No.	A027961799
Accessory	USE-753
Accessory S/N	A027961799
Cell	
Ref. beam	
Measurement date	2017/1/17 9:23
Photometric mode	Abs
Measurement range	800 - 200 nm
Data interval	1 nm
Bandwidth	2.0 nm
Response	0.24 sec
Scan speed	2000 nm/min
Change source at	340 nm
Light source	D2/WI
Filter exchange	Step
Correction	Baseline

[Result of Peak Picking]

No.	Position	Intensity	No.	Position	Intensity
1	257	0.000860114			

Peak Find - 2a.jws

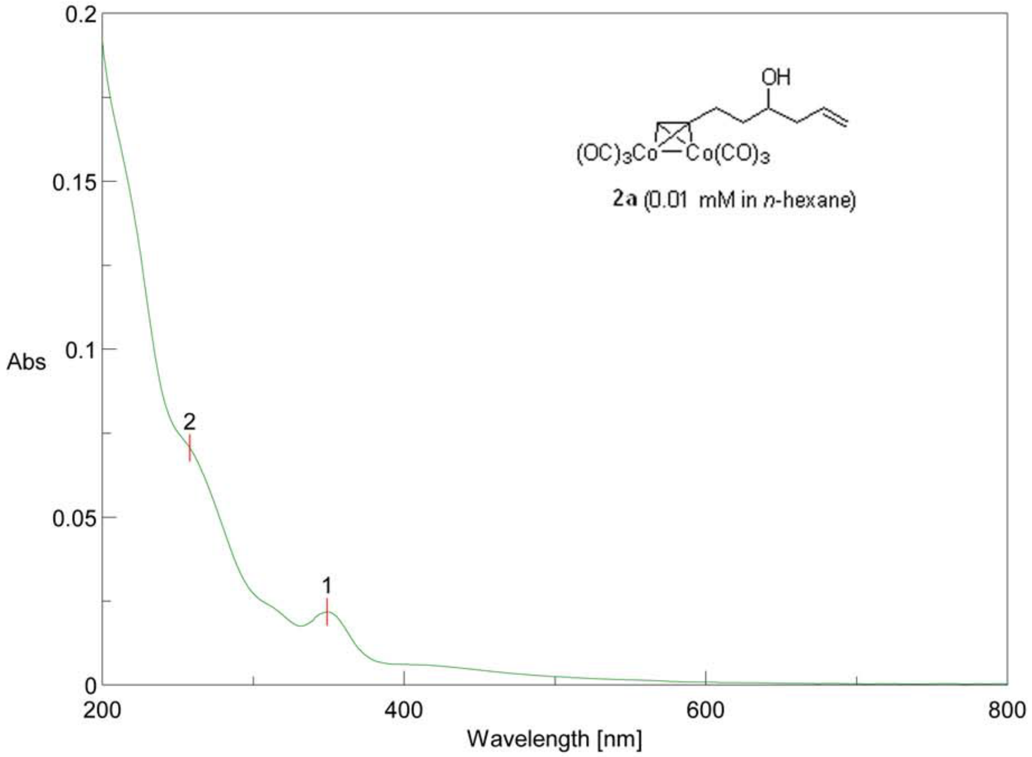

[Detailed Information]

Creation date	2017/1/13 16:50
Data array type	Linear data array
Horizontal axis	Wavelength [nm]
Vertical axis	Abs
Start	800 nm
End	200 nm
Data interval	1 nm
Data points	601

[Measurement Information]

Instrument name	V-750
Model name	V-750
Serial No.	A027961799
Accessory	USE-753
Accessory S/N	A027961799
Cell	
Ref. beam	
Measurement date	2017/1/13 16:49
Photometric mode	Abs
Measurement range	800 - 200 nm
Data interval	1 nm
Bandwidth	2.0 nm
Response	0.24 sec
Scan speed	2000 nm/min
Change source at	340 nm
Light source	D2/WI
Filter exchange	Step
Correction	Baseline

[Result of Peak Picking]

No.	Position	Intensity	No.	Position	Intensity
1	349	0.0216823	2	258	0.0705405

3. ^{1}H- and ^{13}C-NMR spectra of **1b**, and **1g-j**

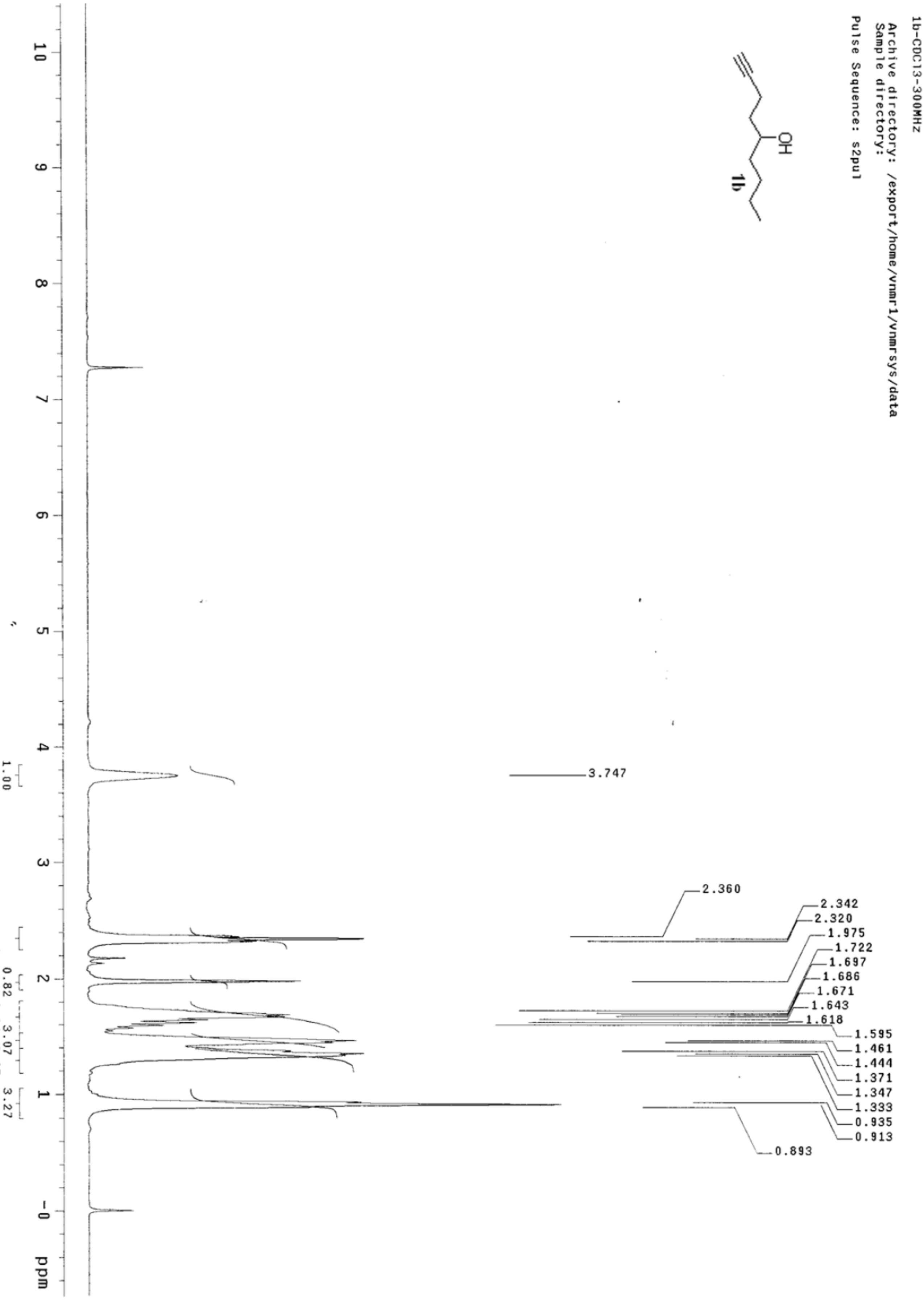

1b-CDCl3-300MHz

Archive directory: /export/home/vnmr1/vnmrsys/data
Sample directory:

Pulse Sequence: s2pul

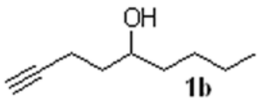

1b

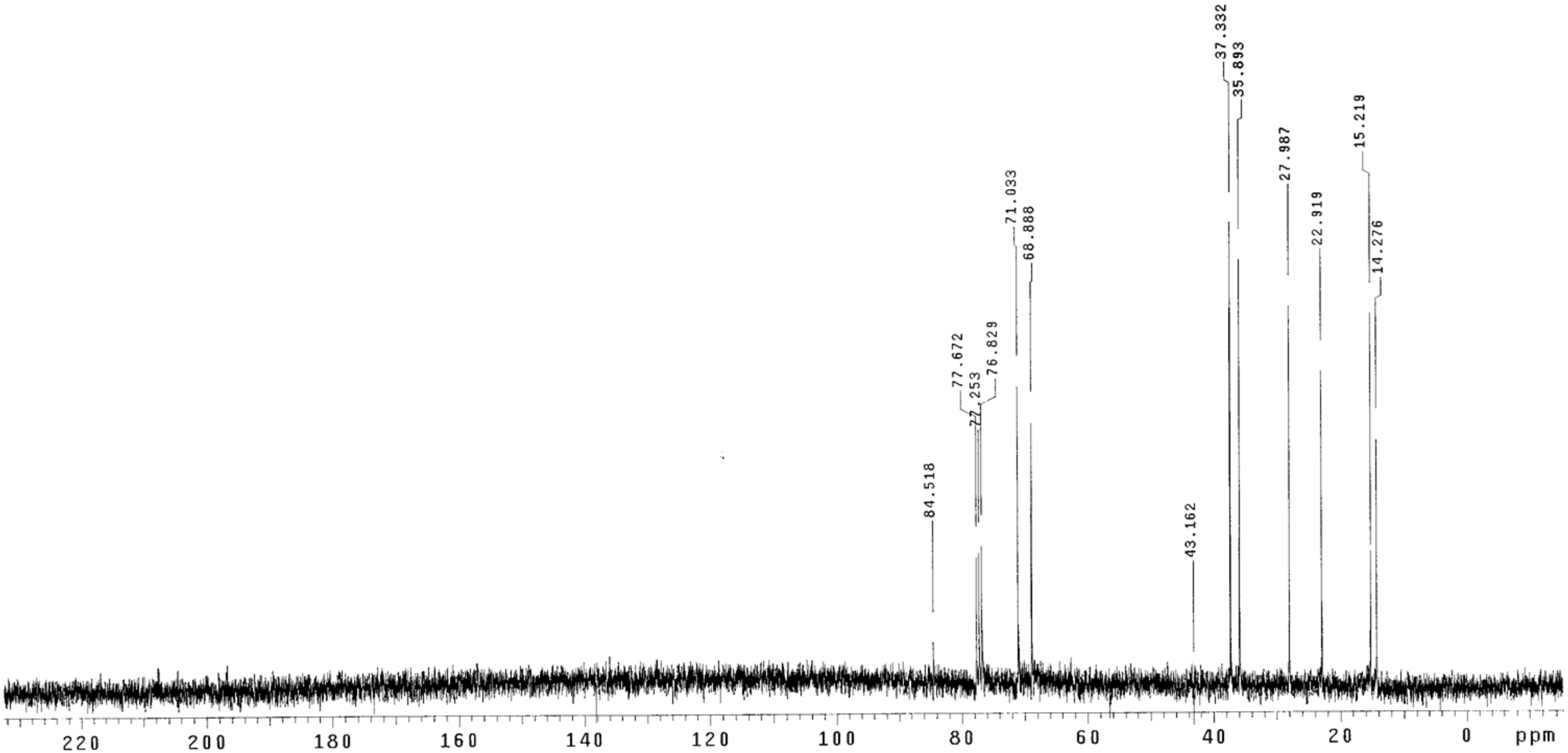

1g-CDCl3-500MHz

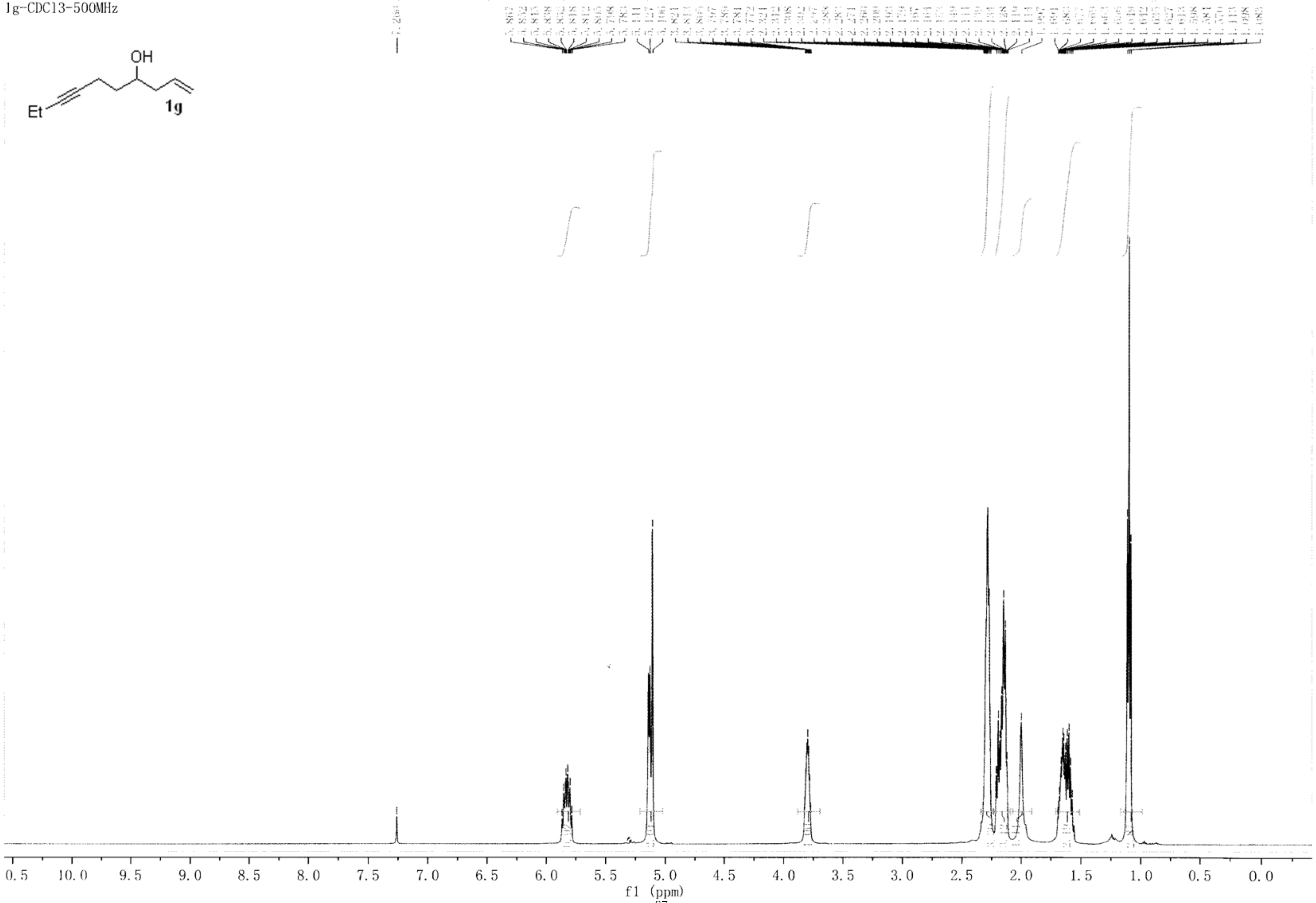

1g-CDCl3-500MHz

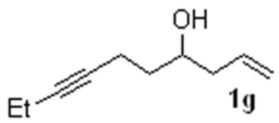

1g

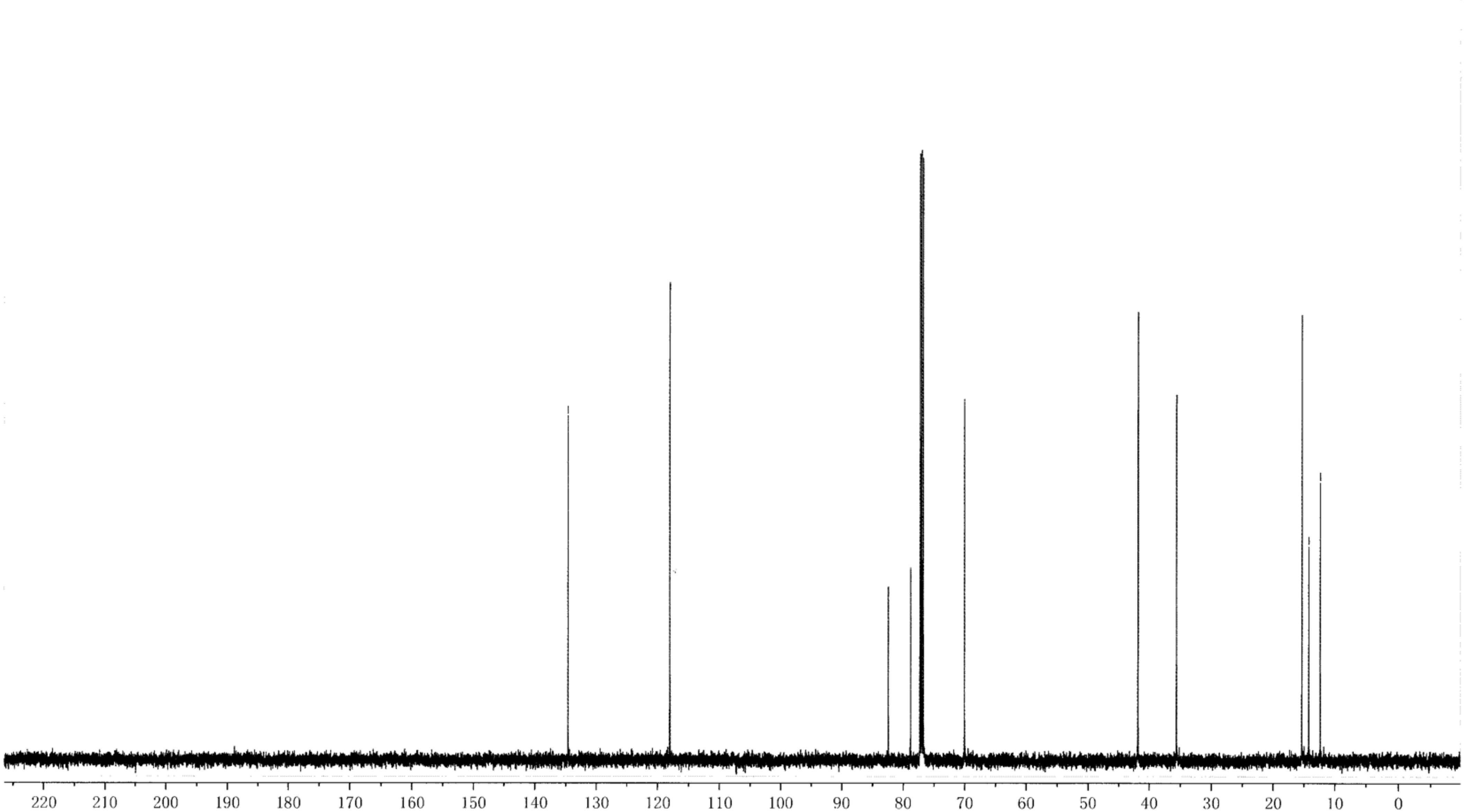

1h-CDCl3-400MHz

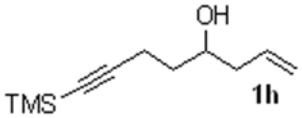

1h

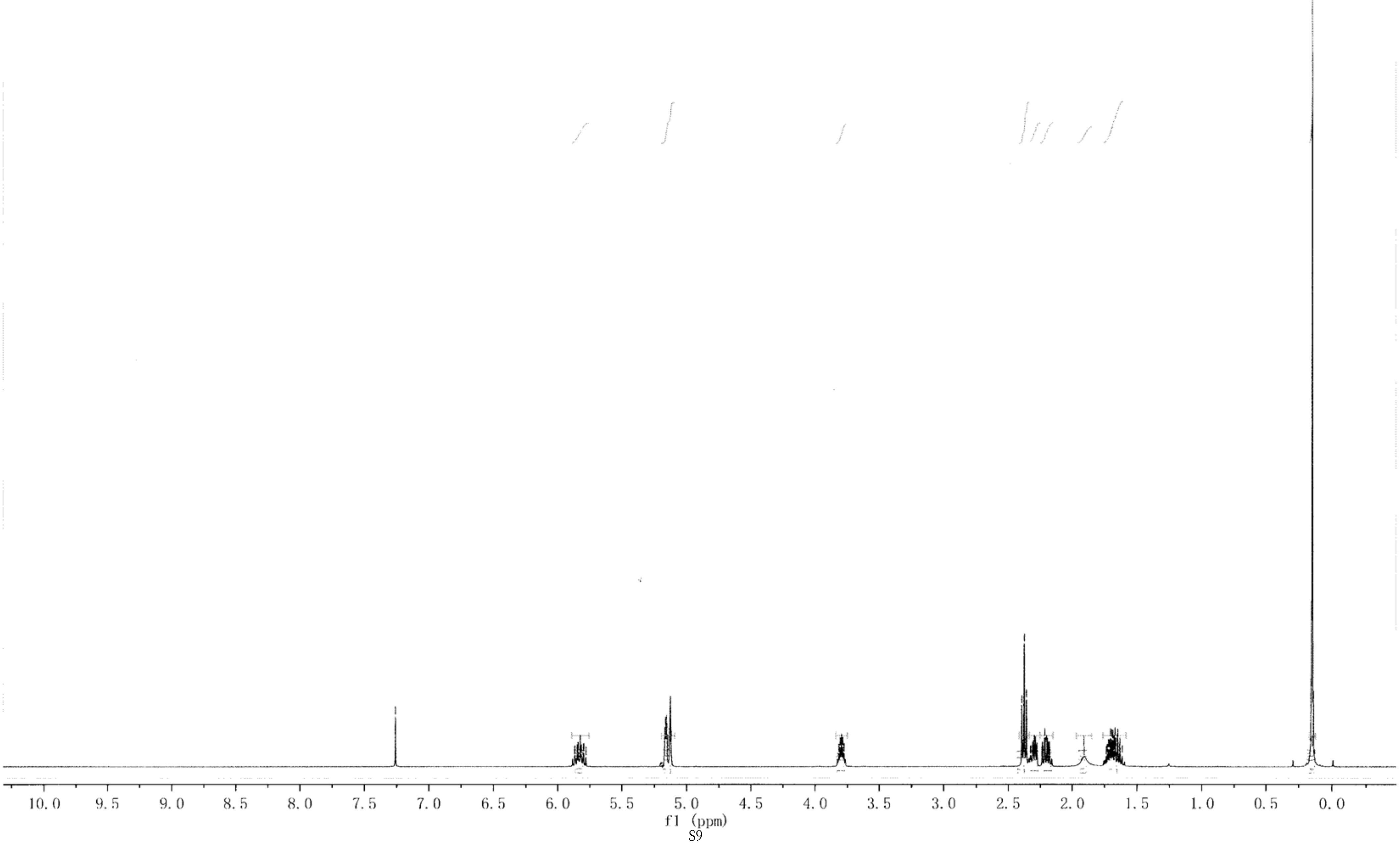

1h-CDCl3-400MHz

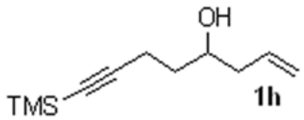

1h

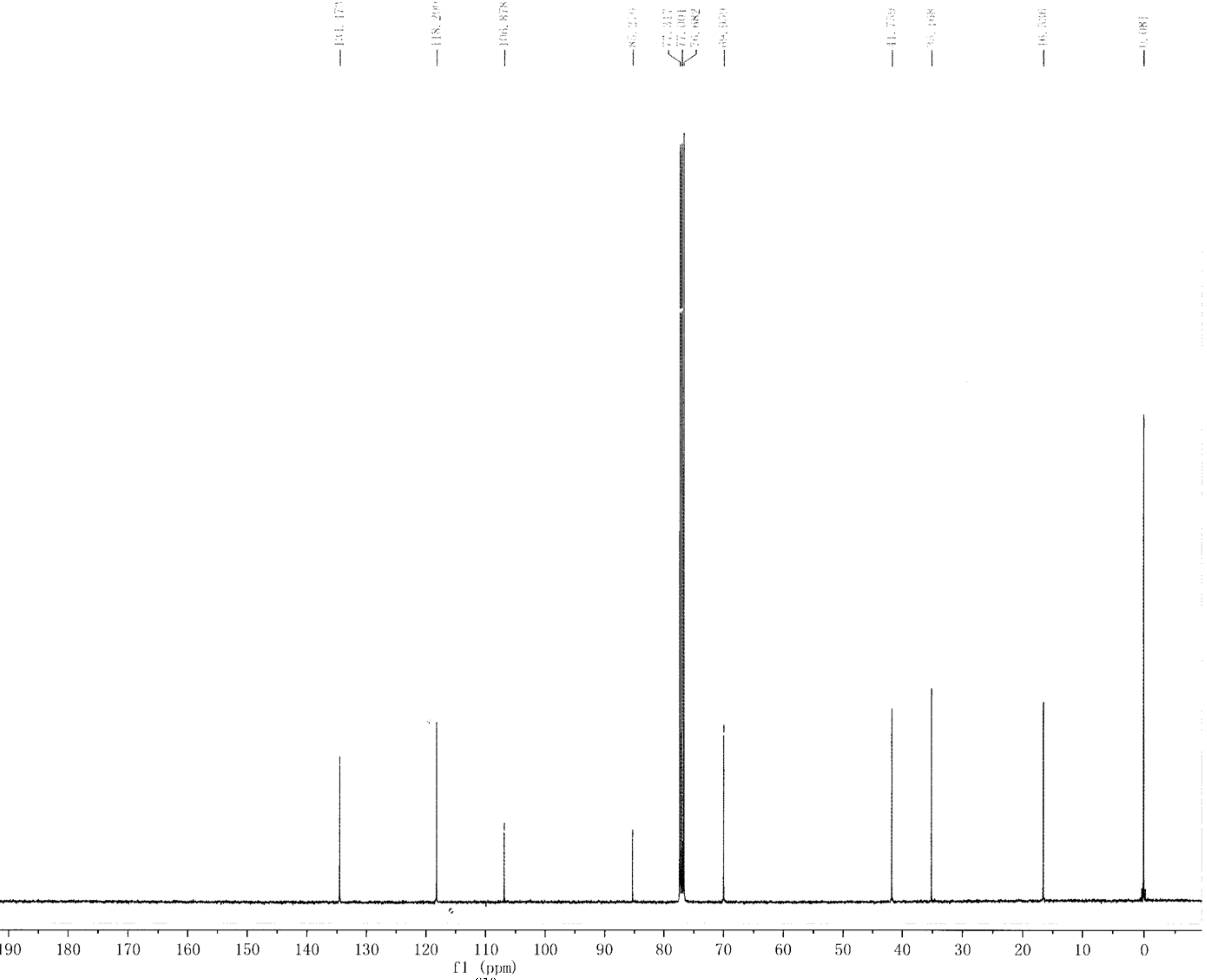

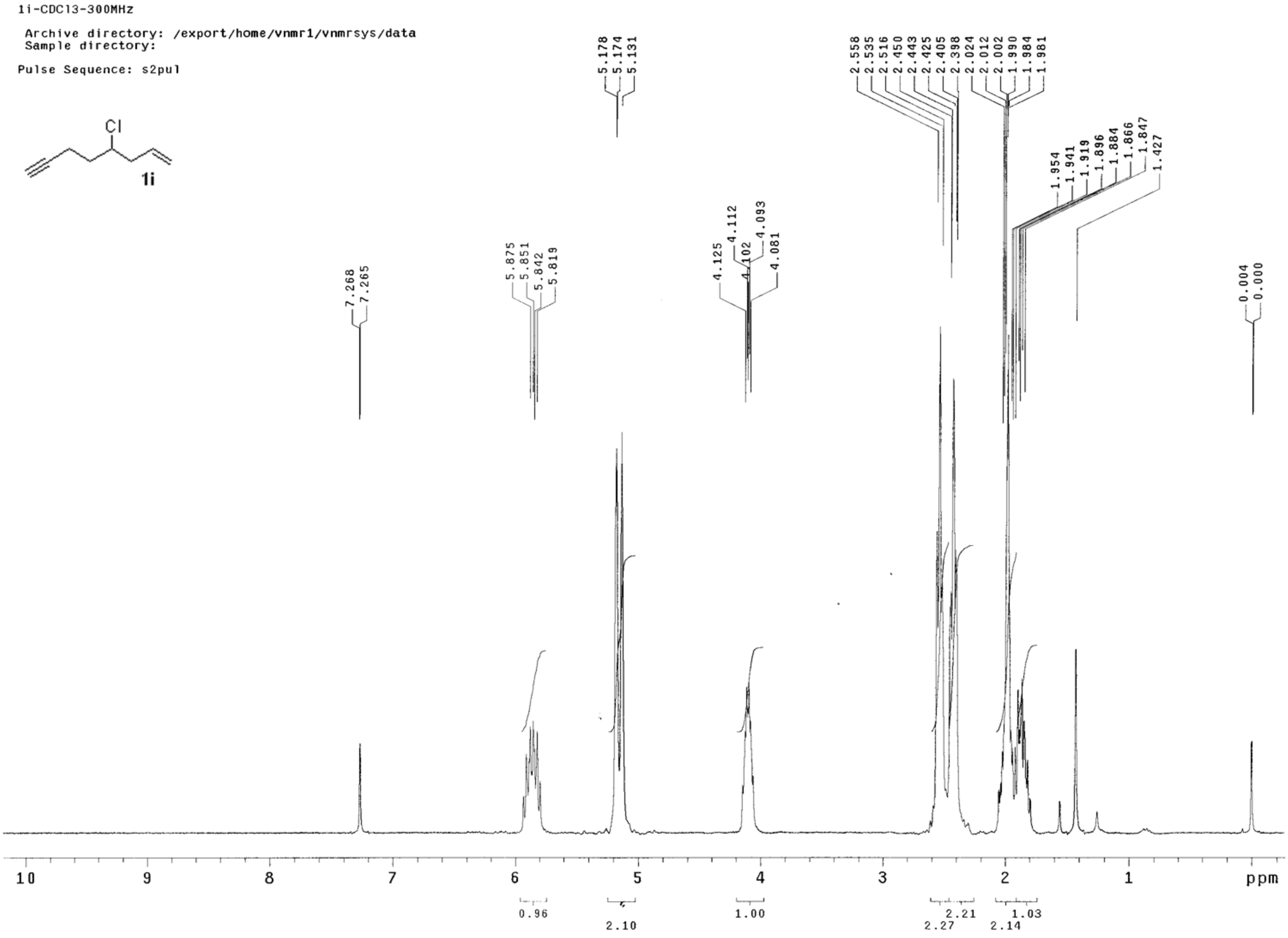
1i-CDCl3-300MHz
Archive directory: /export/home/vnmr1/vnmrsys/data
Sample directory:
Pulse Sequence: s2pul
Cl
1i
7.268
7.265
5.875
5.851
5.842
5.819
5.178
5.174
5.131
4.125
4.112
4.102
4.093
4.081
2.558
2.535
2.516
2.450
2.443
2.425
2.405
2.398
2.024
2.012
2.002
1.990
1.984
1.981
1.954
1.941
1.919
1.896
1.884
1.866
1.847
1.427
0.004
0.000
10
9
8
7
6
5
4
3
2
1
ppm
0.96
2.10
1.00
2.21
2.27
1.03
2.14

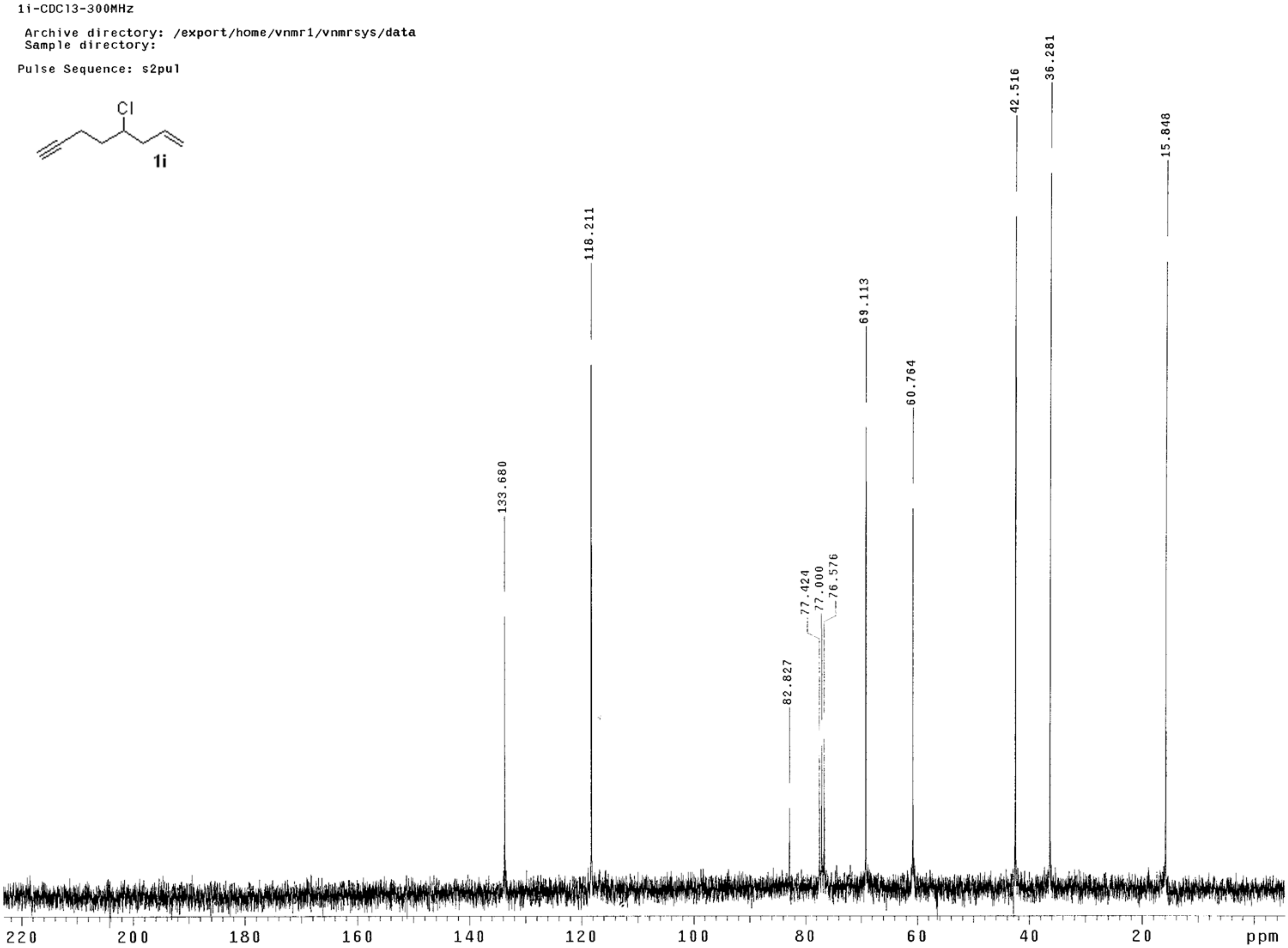

1i-CDCl3-300MHz
Archive directory: /export/home/vnmr1/vnmrsys/data
Sample directory:
Pulse Sequence: s2pul
Cl
1i
133.680
118.211
82.827
77.424
77.000
76.576
69.113
60.764
42.516
36.281
15.848
220
200
180
160
140
120
100
80
60
40
20
ppm

1j-CDCl3-300MHz

Archive directory: /export/home/vnmr1/vnmrsys/data
Sample directory:

Pulse Sequence: s2pul

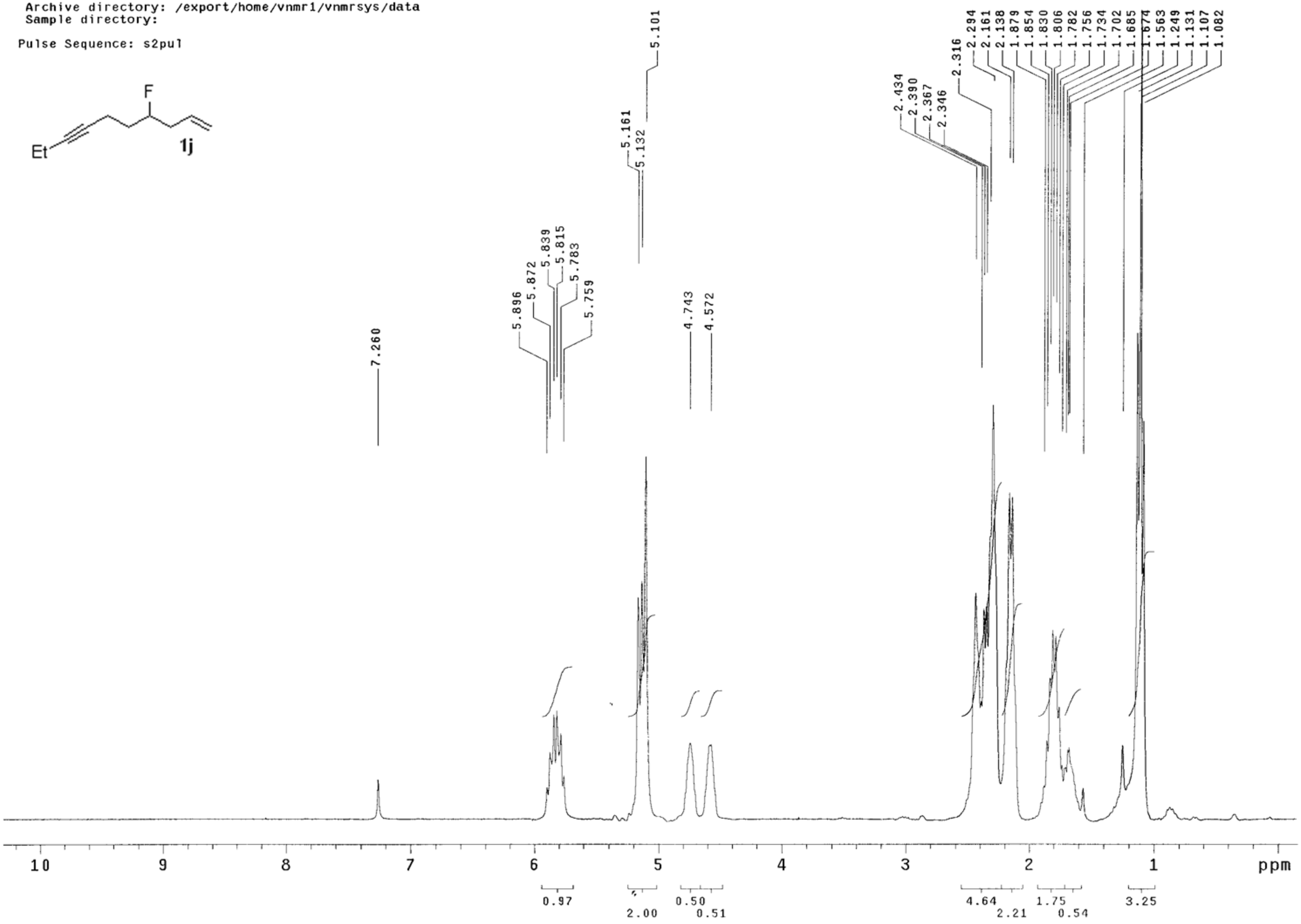

1j-CDCl3-300MHz

Archive directory: /export/home/vnmr1/vnmrsys/data
Sample directory:

Pulse Sequence: s2pul

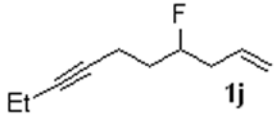

1j

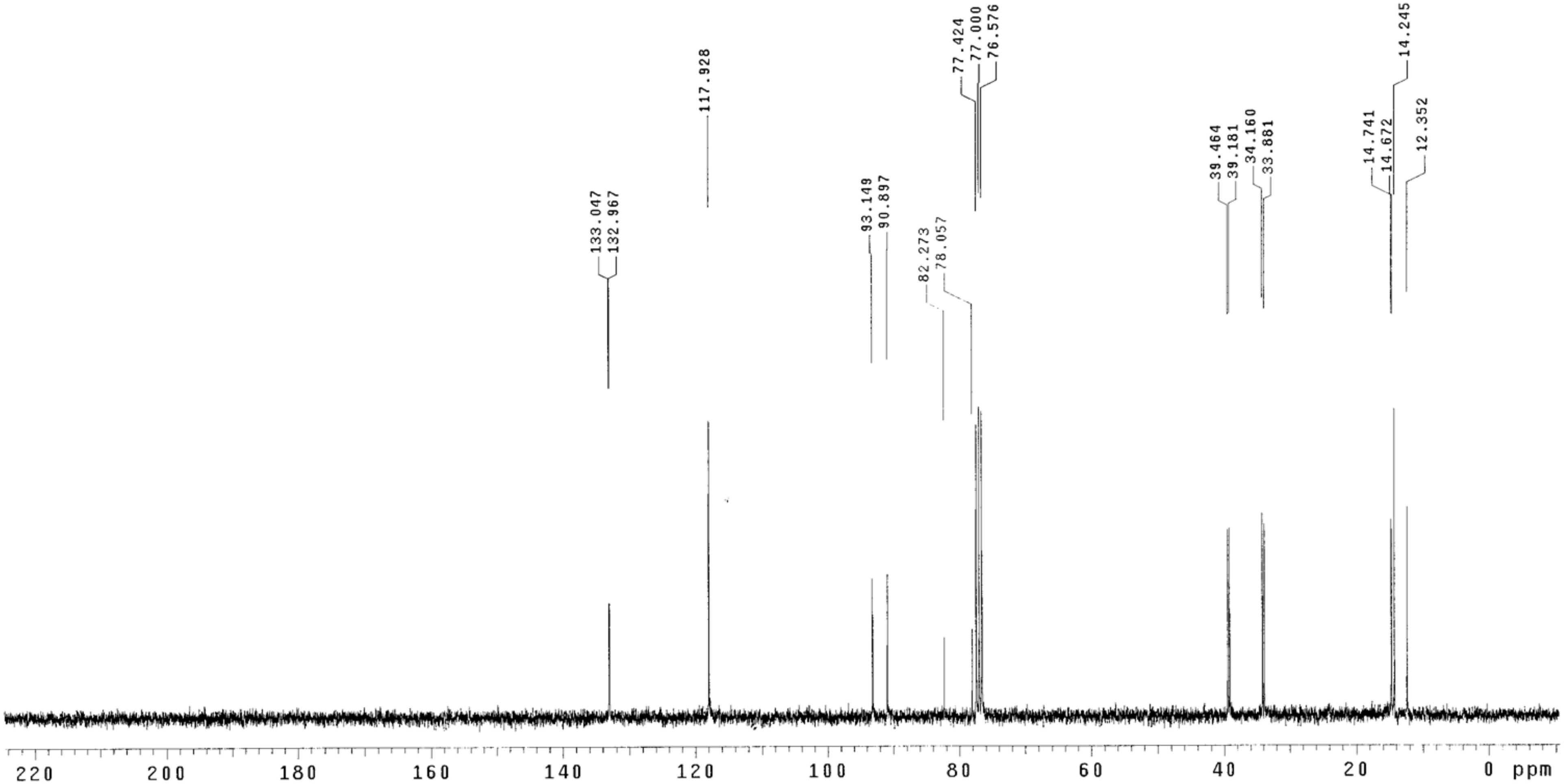

4. HPLC chart of **1a**

Shimadzu CLASS-VP V6.14 SP1 ***Area % Report***

Page 1 of 1

Method Name: C:\CLASS-VP\untitled.met
Data Name: D:\CLASS-VP6.14\Data\Xu\1a-0.4iPrOH-1mL-200nm-1M.dat
User: System
Acquired: 2017-1-16 20:57:59
Printed: 2017-1-16 21:24:38

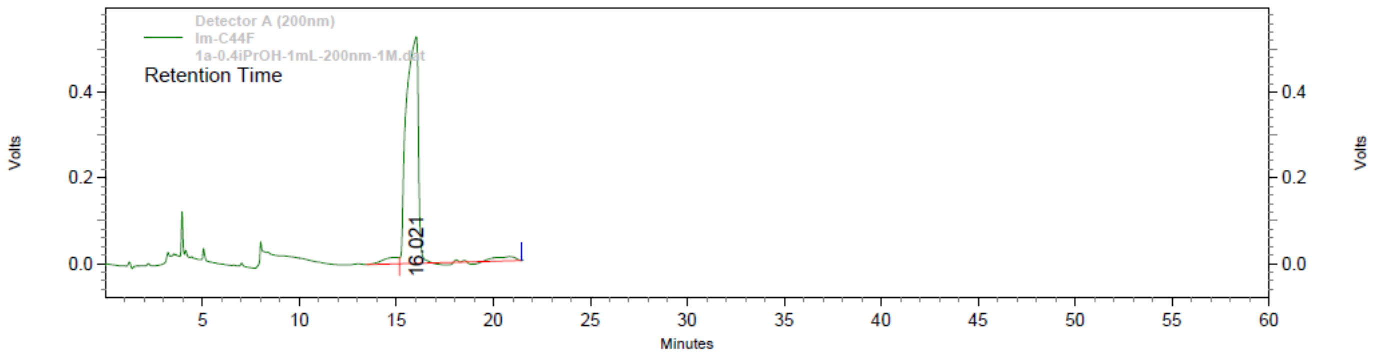

Detector A (200nm)

Pk #	Retention Time	Area	Area %	Height	Height %
1	16.021	21456284	100.000	527922	100.000
Totals		21456284	100.000	527922	100.000

5. ^{1}H-, ^{13}C-NMR spectra, and HPLC charts of **2a-k**

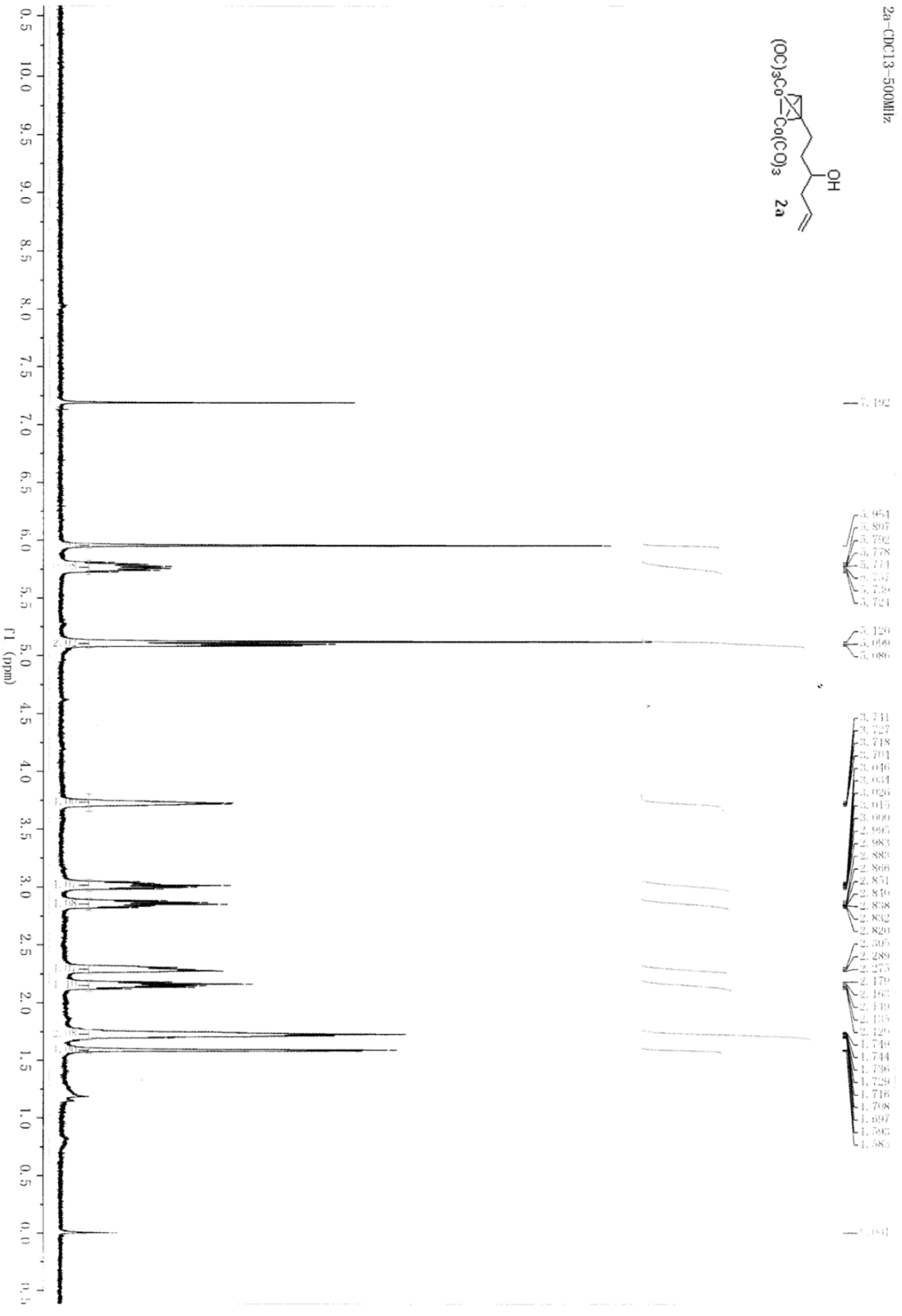

2a-CDCl3-500MHz

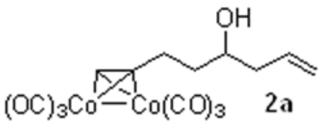

2a

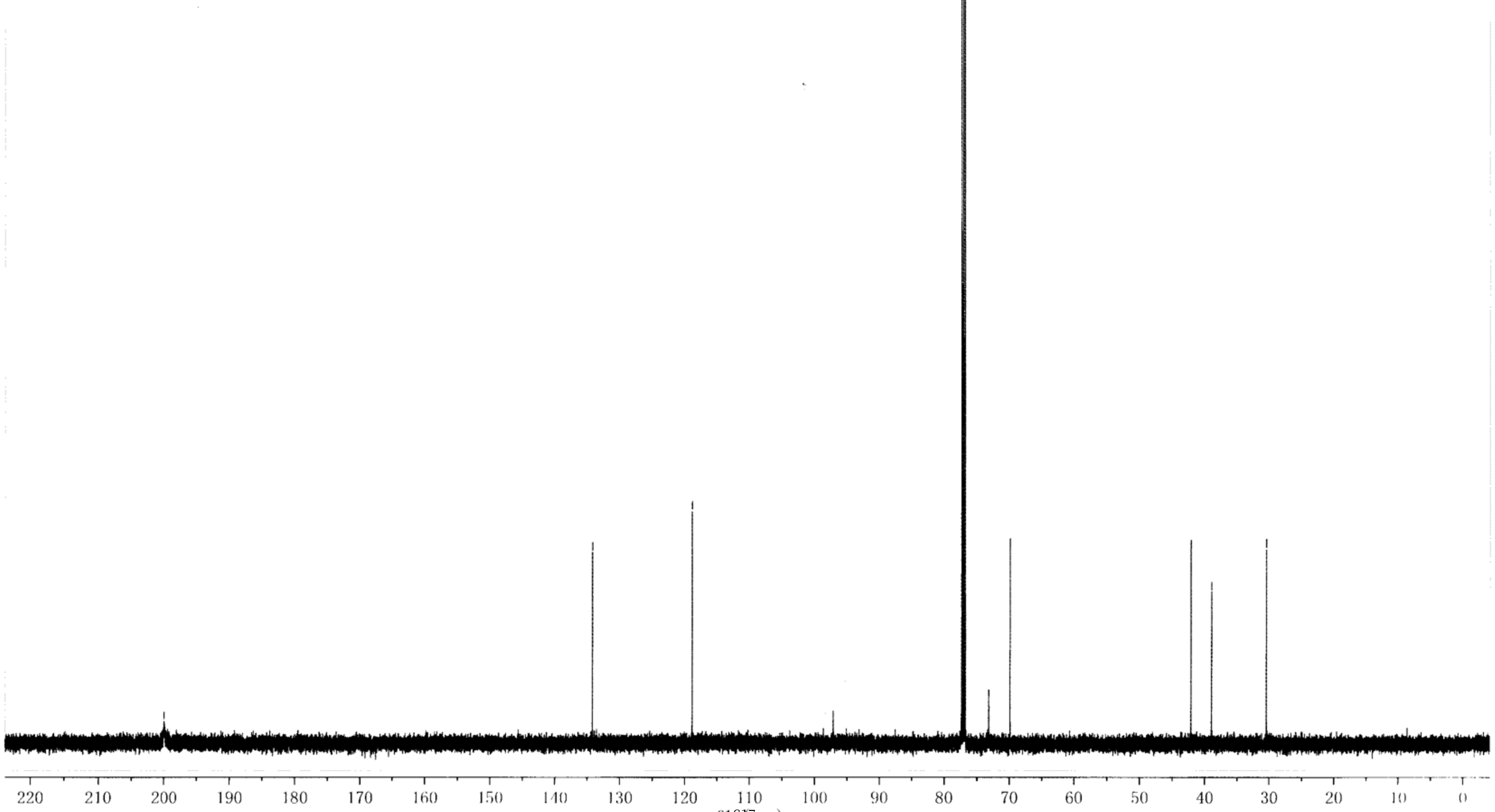

Method Name: C:\CLASS-VP\untitled.met
Data Name: D:\CLASS-VP6.14\Data\Xu\2a-0.4iPrOH-1mL.dat
User: System
Acquired: 2017-1-13 17:26:37
Printed: 2017-1-13 18:19:03

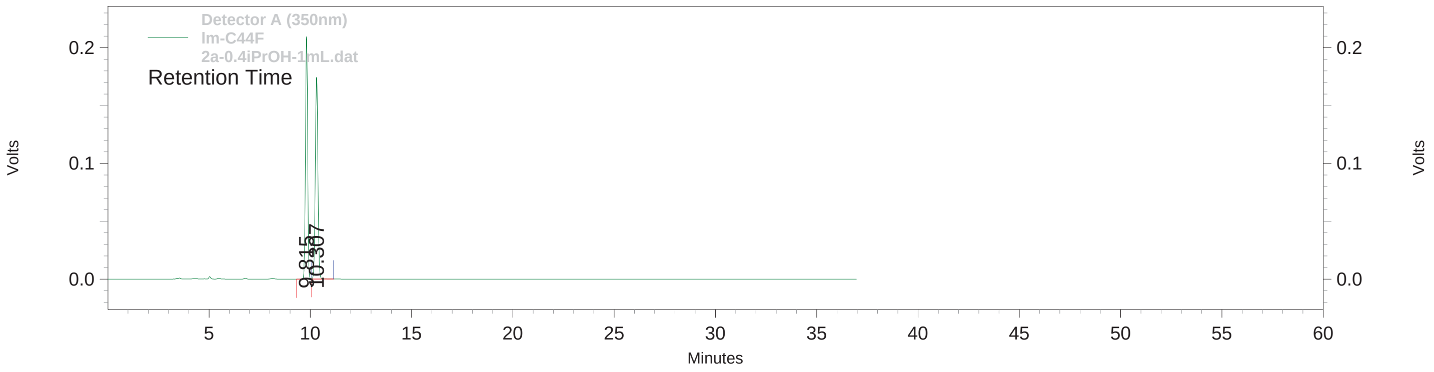

Detector A (350nm)

Pk #	Retention Time	Area	Area %	Height	Height %
1	9.815	1552716	49.874	209589	54.604
2	10.307	1560583	50.126	174244	45.396
Totals		3113299	100.000	383833	100.000

HPLC analysis of racemic 2a
.
Chiralpak-IB column
eluting solvent: 2-PrOH/n-hexane = 0.4:99.6
flow rate: 1 mL/min
column temperature: 25°C
detection wavelength: 350 nm
.
retention factors: 2.067, 2.221
selective factor: 1.075

Method Name: **{Method Name}**
Data Name: **D:\CLASS-VP6.14\Data\Xu\2a-chiral-0.4iPrOH-1mL.dat**
User: **System**
Acquired: **2017-3-3 14:40:07**
Printed: **2017-3-6 10:35:09**

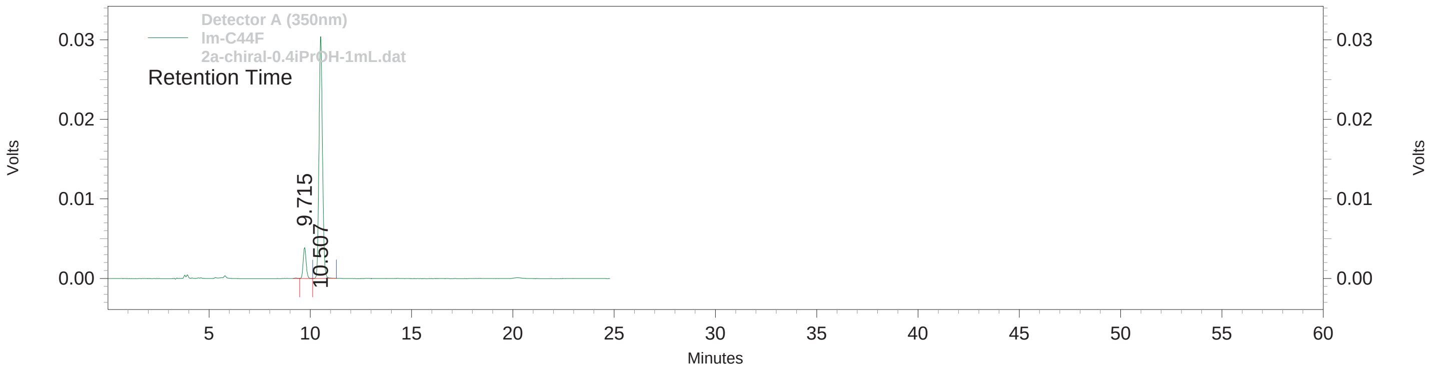

Detector A (350nm)

Pk #	Retention Time	Area	Area %	Height	Height %
1	9.715	39426	9.797	3882	11.318
2	10.507	363007	90.203	30420	88.682

Totals					
		402433	100.000	34303	100.000

HPLC analysis of enantioenriched 2a
.
Chiralpak-IB column
eluting solvent: 2-PrOH/n-hexane = 0.4:99.6
flow rate: 1 mL/min
column temperature: 25°C
detection wavelength: 350 nm
.
retention factors: 2.036, 2.283
selective factor: 1.121

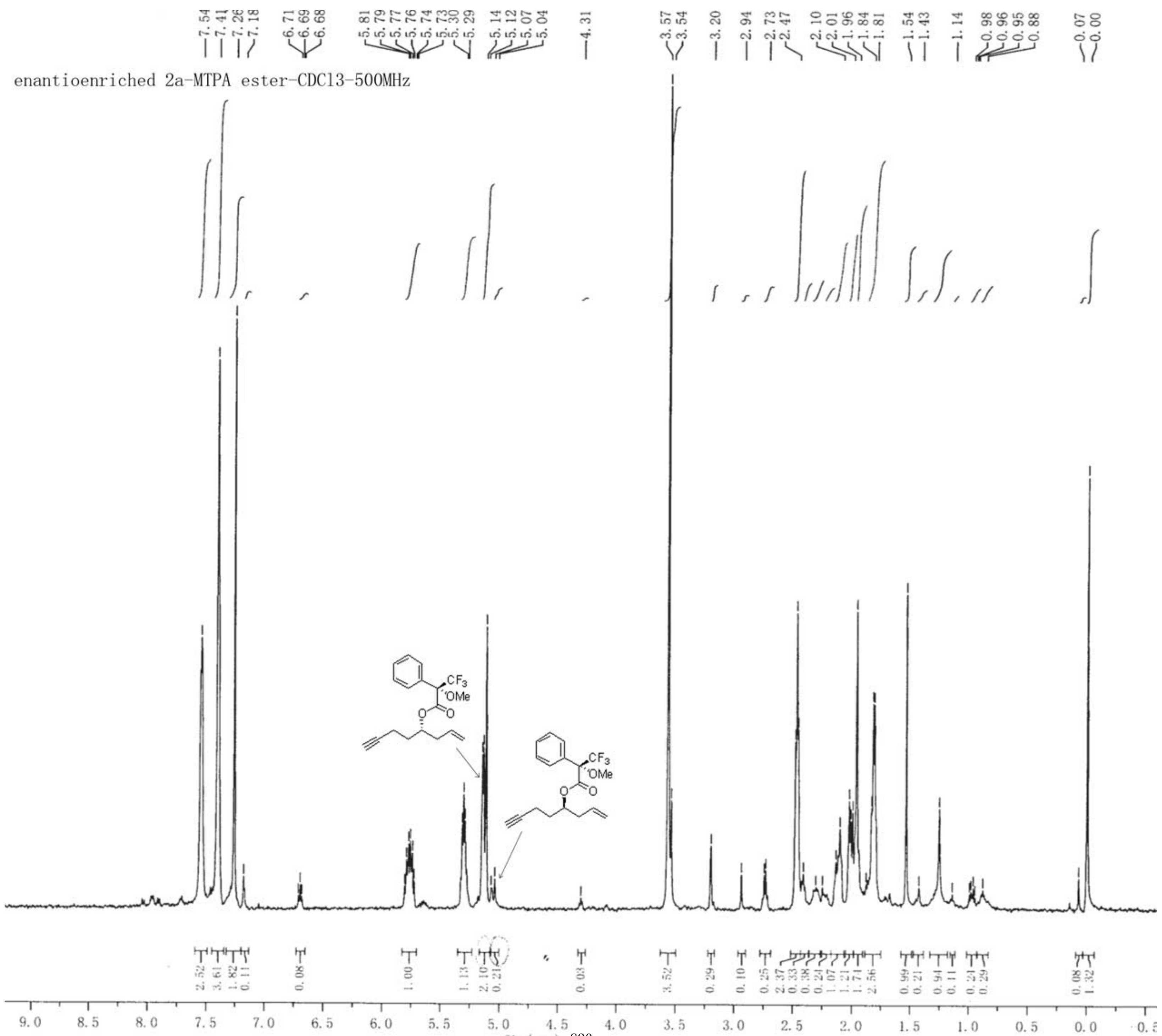

enantioenriched 2a-MTPA ester-CDCl3-500MHz
CF3
OMe
O
f1 (ppm)

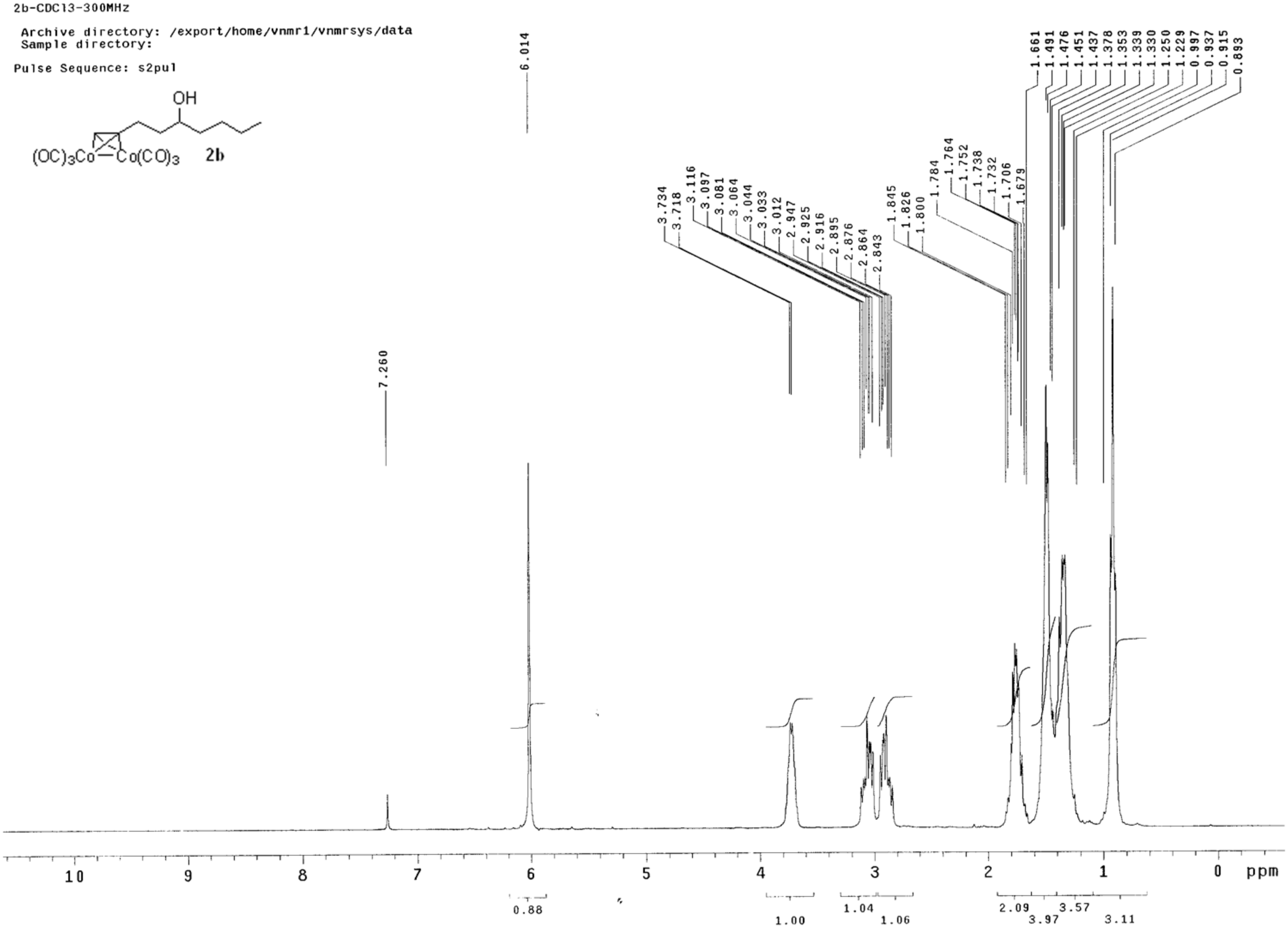
2b-CDCl3-300MHz
Archive directory: /export/home/vnmr1/vnmrsys/data
Sample directory:
Pulse Sequence: s2pul
OH
(OC)3Co Co(CO)3 2b
7.260
6.014
3.734 3.718 3.116 3.097 3.081 3.064 3.044 3.033 3.012 2.947 2.925 2.916 2.895 2.876 2.864 2.843
1.845 1.826 1.800 1.784 1.764 1.752 1.738 1.732 1.706 1.679
1.661 1.491 1.476 1.451 1.437 1.378 1.353 1.339 1.330 1.250 1.229 0.997 0.937 0.915 0.893
10 9 8 7 6 5 4 3 2 1 0 ppm
0.88 1.00 1.04 1.06 2.09 3.97 3.57 3.11

2b-CDCl3-300MHz

Archive directory: /export/home/vnmr1/vnmrsys/data
Sample directory:

Pulse Sequence: s2pul

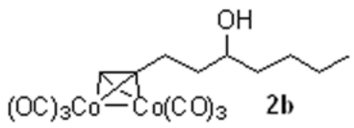

2b

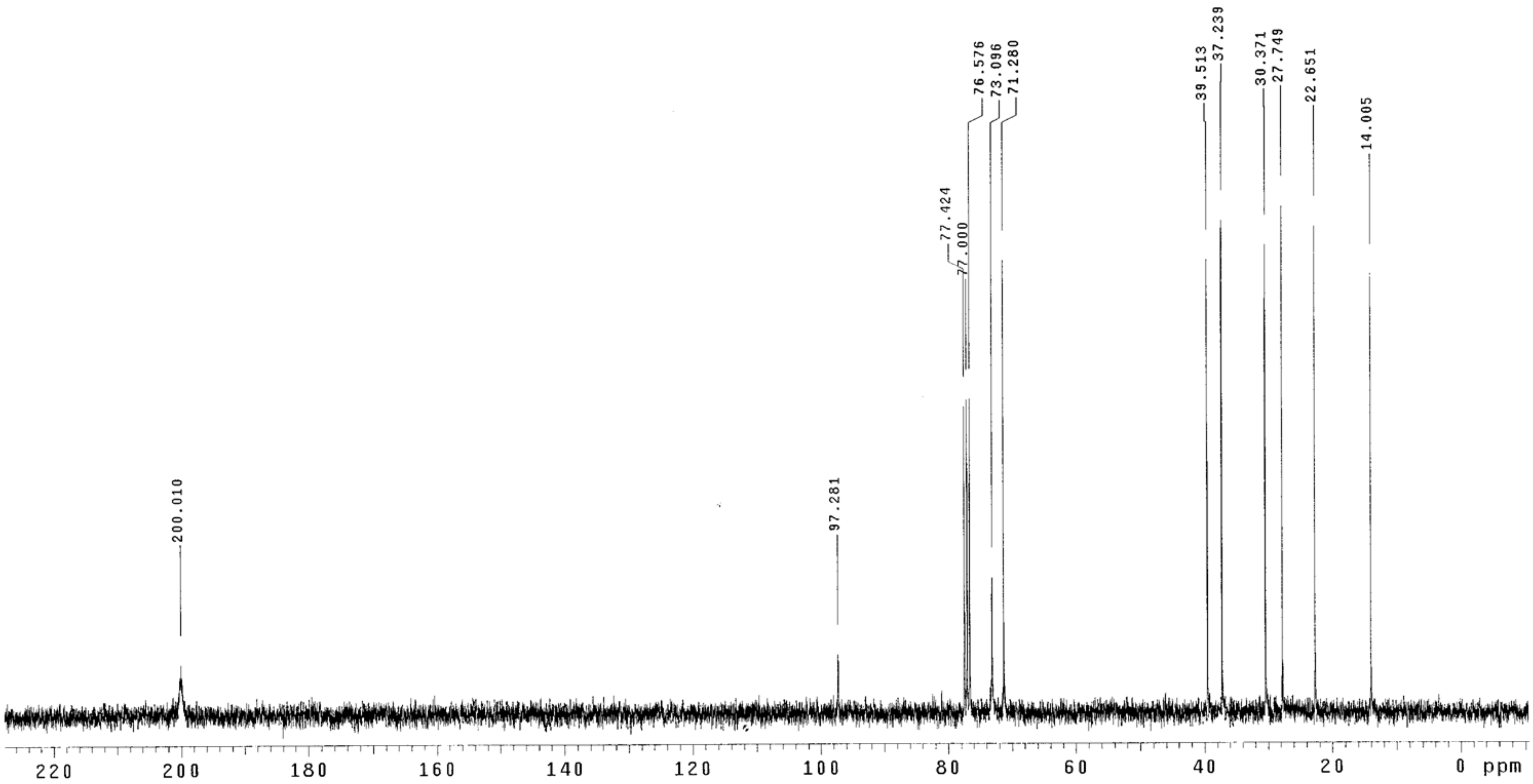

Method Name: **C:\CLASS-VP\untitled.met**
Data Name: **D:\CLASS-VP6.14\Data\Xu\2b-0.4iPrOH-1mL-1.dat**
User: **System**
Acquired: **2016-12-25 10:50:33**
Printed: **2017-1-6 19:54:53**

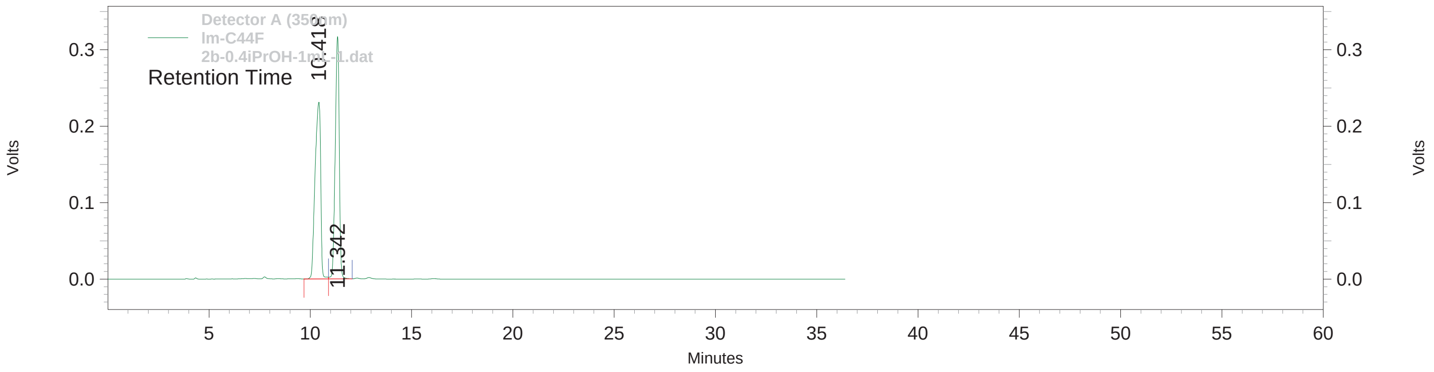

Detector A (350nm)

Pk #	Retention Time	Area	Area %	Height	Height %
1	10.418	4434589	50.300	231032	42.211
2	11.342	4381753	49.700	316294	57.789
Totals		8816341	100.000	547326	100.000

HPLC analysis of 2b
.
Chiralpak-IB column
eluting solvent: 2-PrOH/n-hexane = 0.4:99.6
flow rate: 1 mL/min
column temperature: 25°C
detection wavelength: 350 nm
.
retention factors: 2.256, 2.544
selective factor: 1.128

2c-CDCl3-300MHz

Archive directory: /export/home/vnmr1/vnmrsys/data
Sample directory:

Pulse Sequence: s2pul

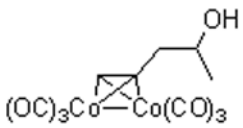

2c

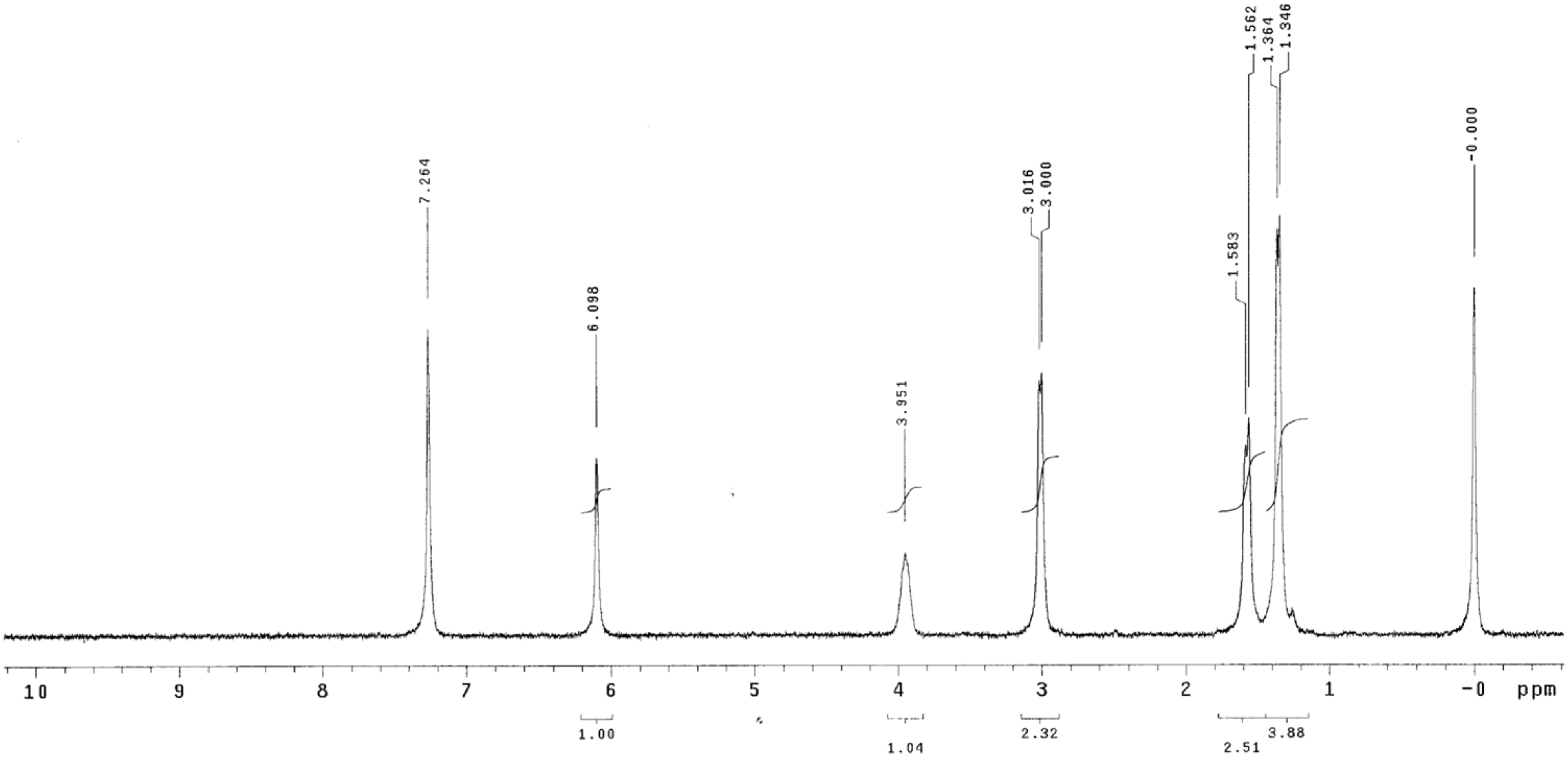

2c-CDCl3-300MHz

Archive directory: /export/home/vnmr1/vnmrsys/data
Sample directory:

Pulse Sequence: s2pul

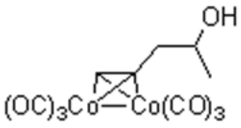

2c

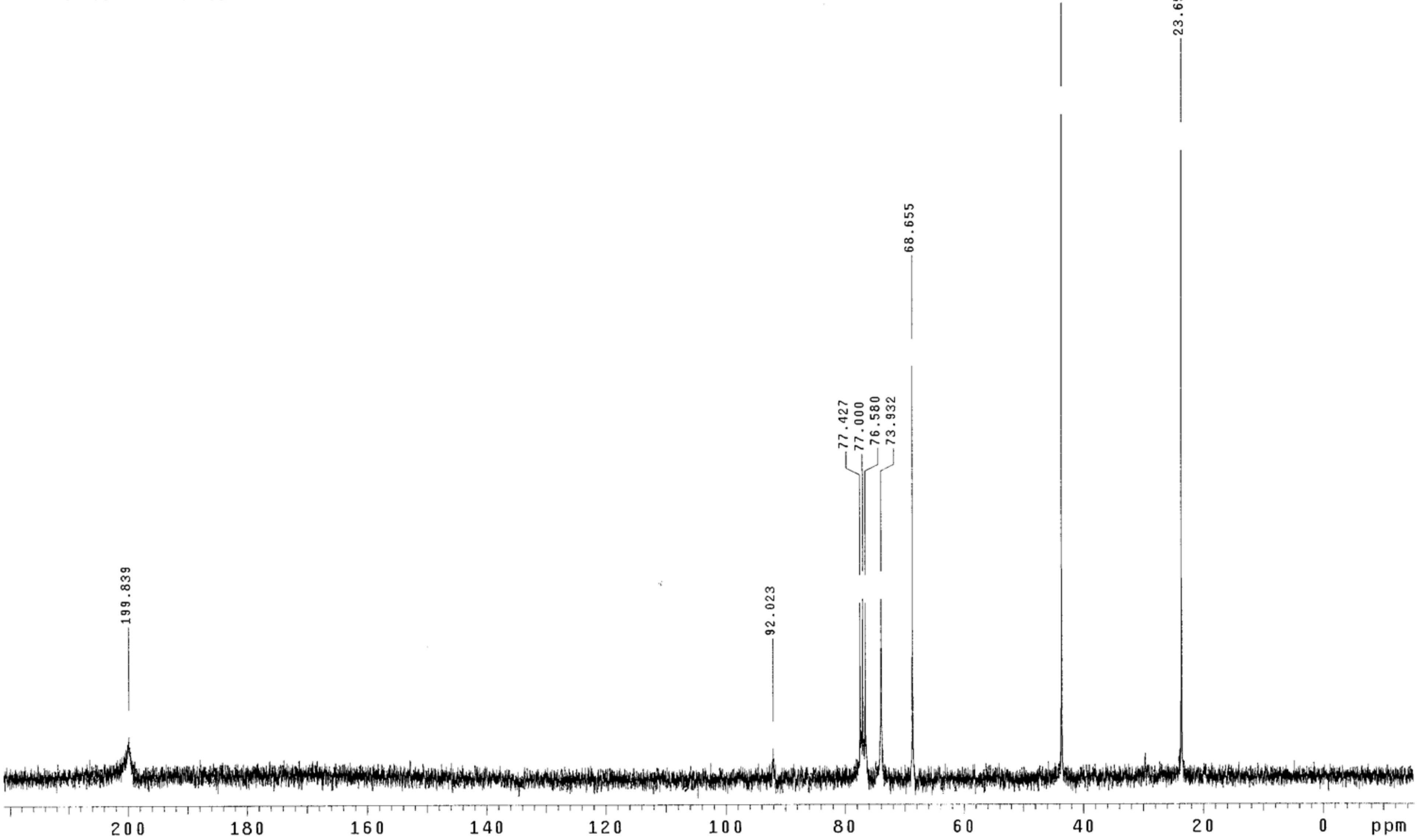

Shimadzu CLASS-VP V6.14 SP1 ***Area % Report***

Method Name: **C:\CLASS-VP\untitled.met**
Data Name: **D:\CLASS-VP6.14\Data\Xu\2c-0.4iPrOH-1mL-1.dat**
User: **System**
Acquired: **2016-11-25 8:36:03**
Printed: **2017-1-6 14:48:08**

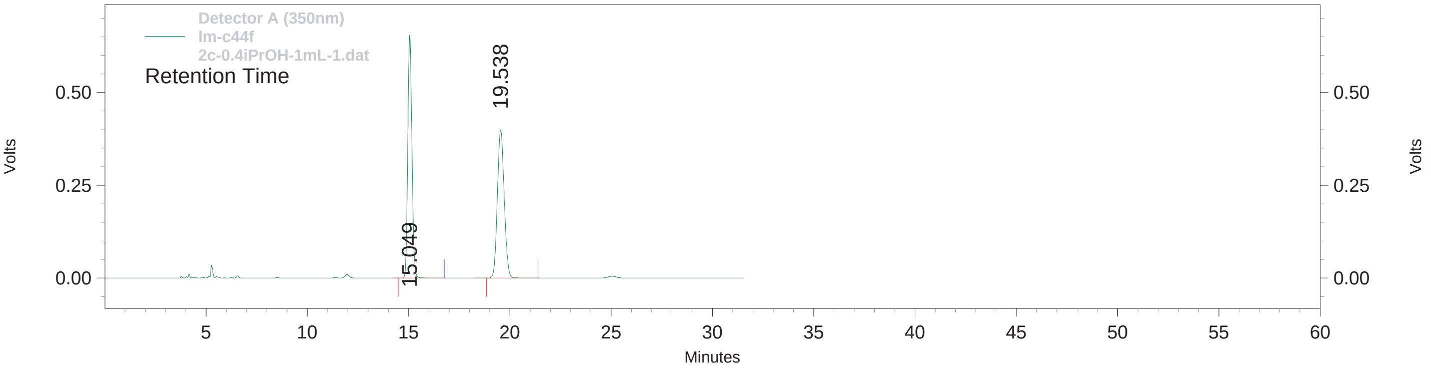

Detector A (350nm)

Pk #	Retention Time	Area	Area %	Height	Height %
1	15.049	9328973	49.952	654684	62.179
2	19.538	9346801	50.048	398213	37.821
Totals		18675773	100.000	1052897	100.000

HPLC analysis of 2c
.
Chiralpak-IB column
eluting solvent: 2-PrOH/n-hexane = 0.4:99.6
flow rate: 1 mL/min
column temperature: 25°C
detection wavelength: 350 nm
.
retention factors: 3.703, 5.106
selective factor: 1.379

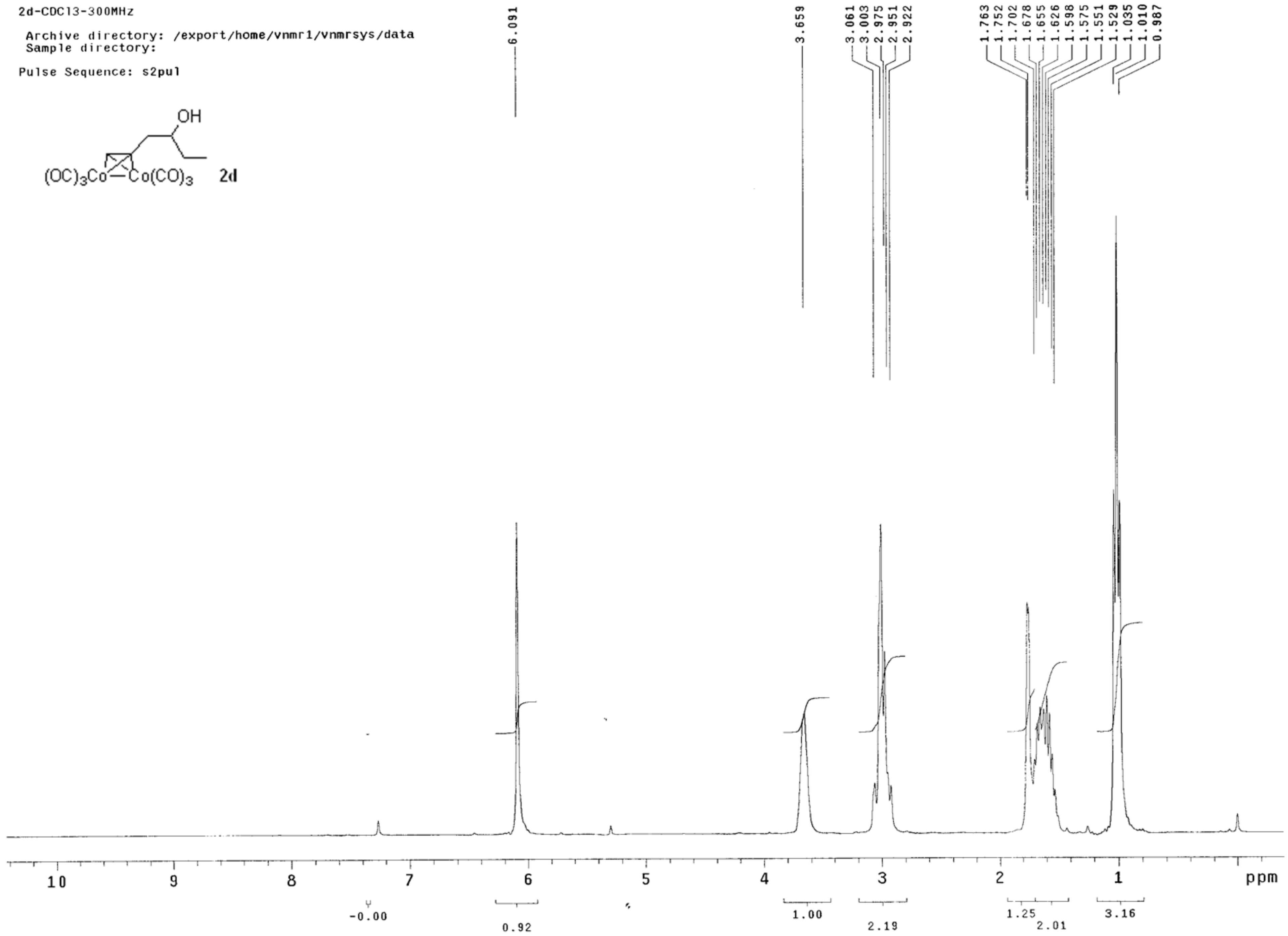
2d-CDCl3-300MHz
Archive directory: /export/home/vnmr1/vnmrsys/data
Sample directory:
Pulse Sequence: s2pul
OH
(OC)3Co—Co(CO)3
2d
6.091
3.659
3.061
3.003
2.975
2.951
2.922
1.763
1.752
1.702
1.678
1.655
1.626
1.598
1.575
1.551
1.529
1.035
1.010
0.987
10
9
8
7
6
5
4
3
2
1
ppm
-0.00
0.92
1.00
2.19
1.25
2.01
3.16

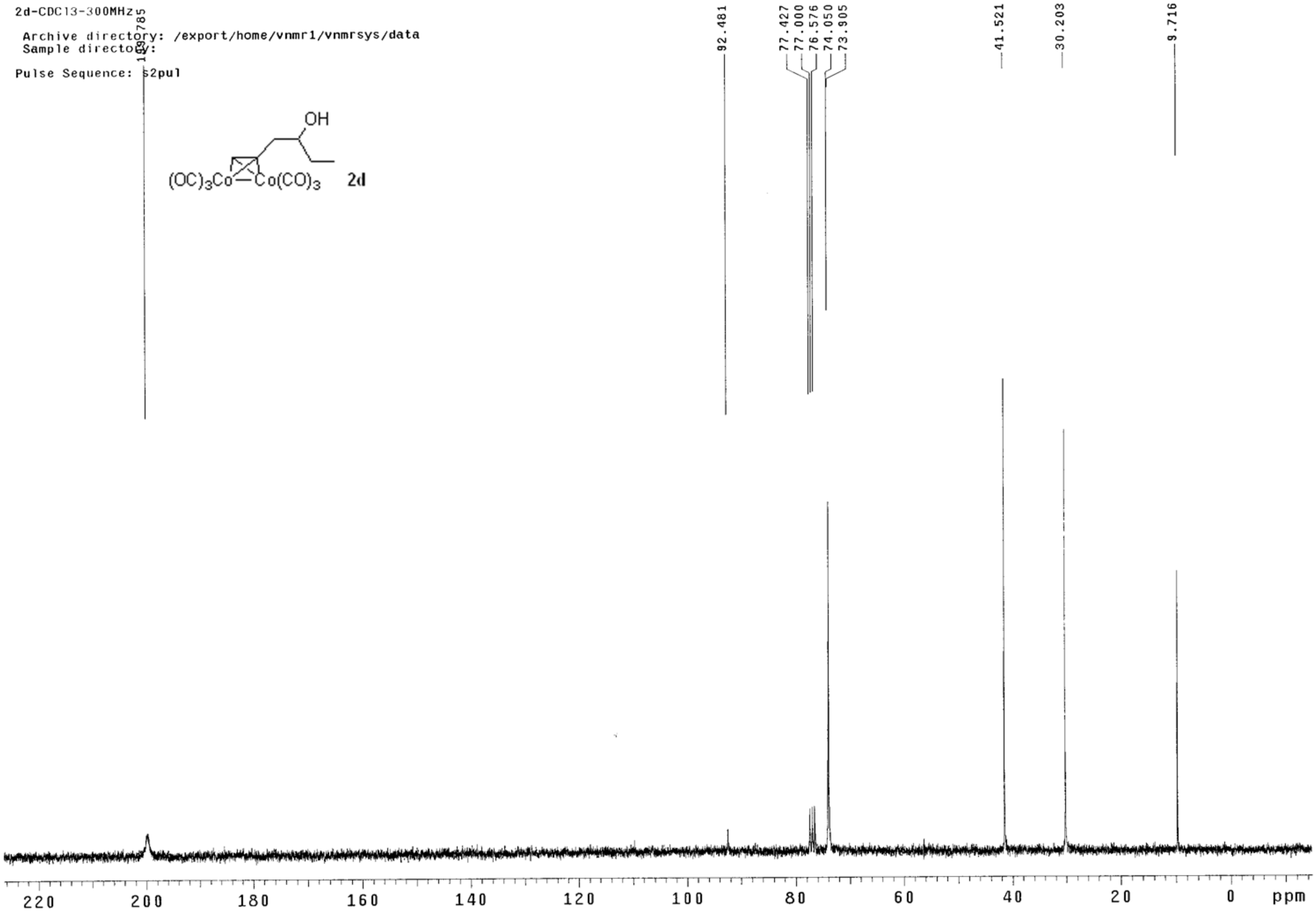
2d-CDCl3-300MHz
Archive directory: /export/home/vnmr1/vnmrsys/data
Sample directory:
Pulse Sequence: s2pul
199.785
92.481
77.427
77.000
76.576
74.050
73.905
41.521
30.203
9.716
OH
(OC)3Co Co(CO)3
2d
220 200 180 160 140 120 100 80 60 40 20 0 ppm

Method Name: **C:\CLASS-VP\untitled.met**
Data Name: **D:\CLASS-VP6.14\Data\Xu\2d-0.4iPrOH-1mL.dat**
User: **System**
Acquired: **2016-12-2 18:37:20**
Printed: **2017-1-6 20:35:40**

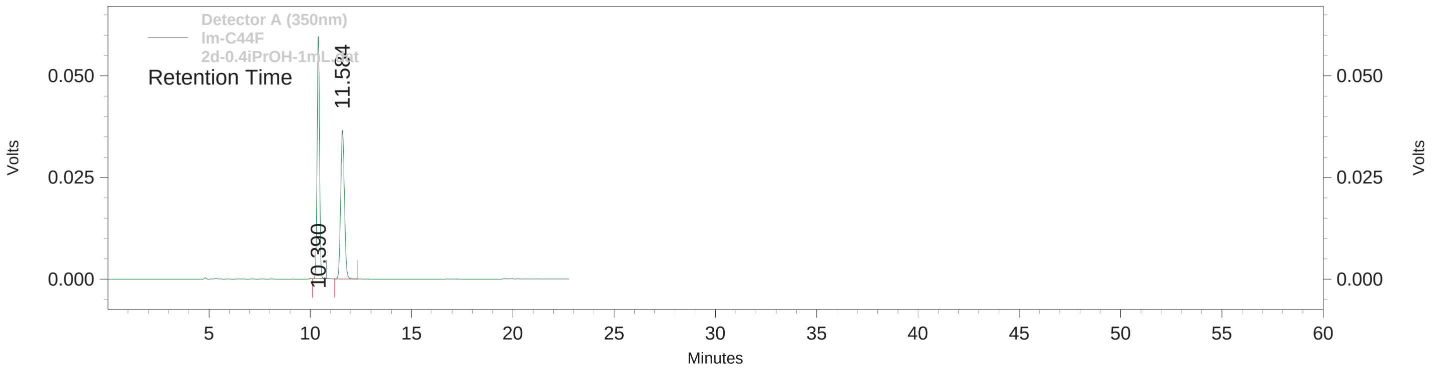

Detector A (350nm)

Pk #	Retention Time	Area	Area %	Height	Height %
1	10.390	499308	51.300	59062	61.800
2	11.584	474009	48.700	36507	38.200
Totals		973316	100.000	95569	100.000

HPLC analysis of 2d

.

Chiralpak-IB column

eluting solvent: 2-PrOH/n-hexane = 0.4:99.6

flow rate: 1 mL/min

column temperature: 25°C

detection wavelength: 350 nm

.

retention factors: 2.247, 2.620

selective factor: 1.166

2e-CDCl3-600MHz

Archive directory: /home/vjwalk/vnmrsys/data/2017Jan/vjwalk
Sample directory: gyl3-37-A

Pulse Sequence: s2pul

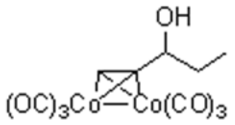

2e

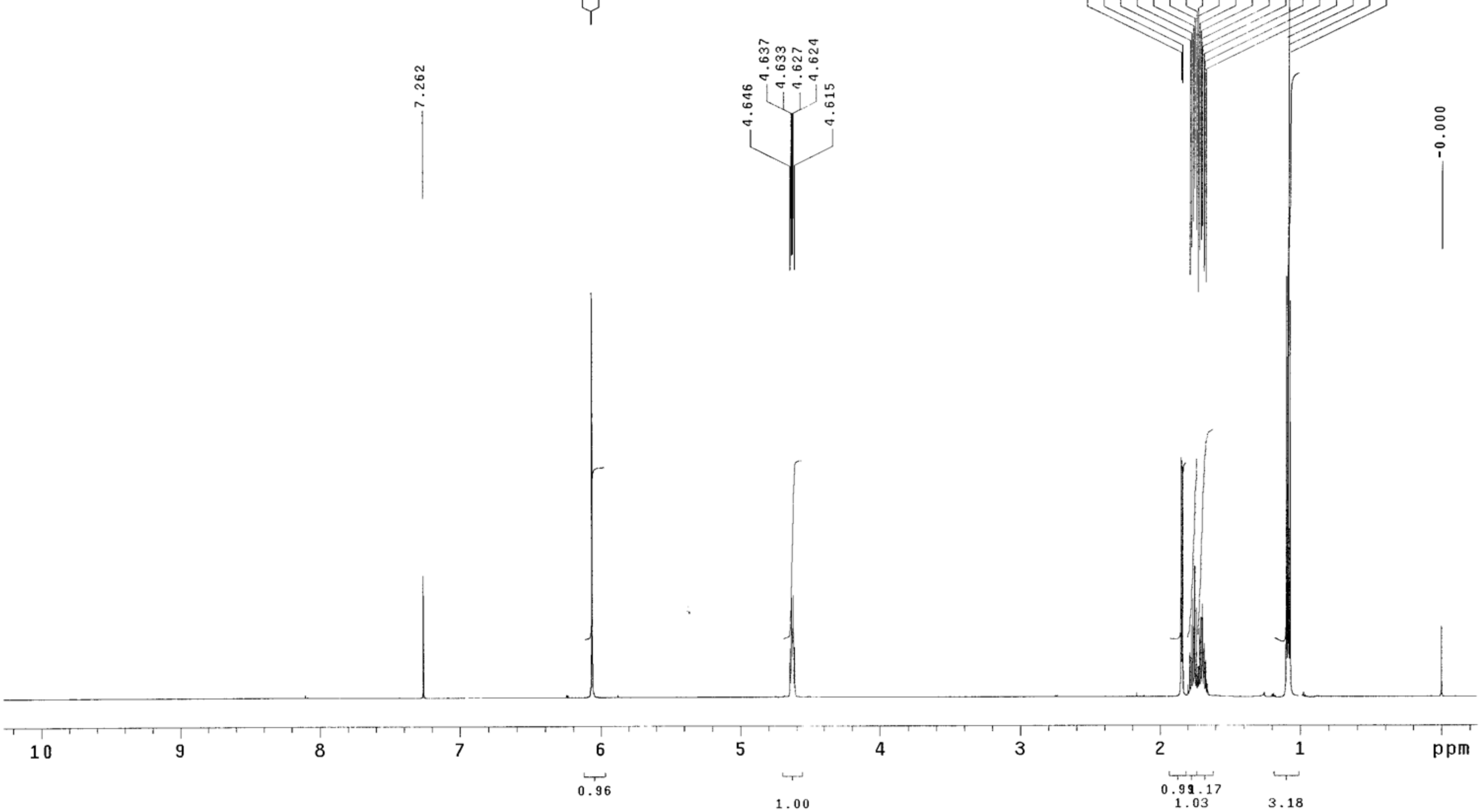

2e-CDCl3-600MHz

Archive directory: /home/vjwalk/vnmrsys/data/2017Jan/vjwalk
Sample directory: gyl3-37-A

Pulse Sequence: s2pul

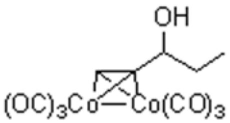

2e

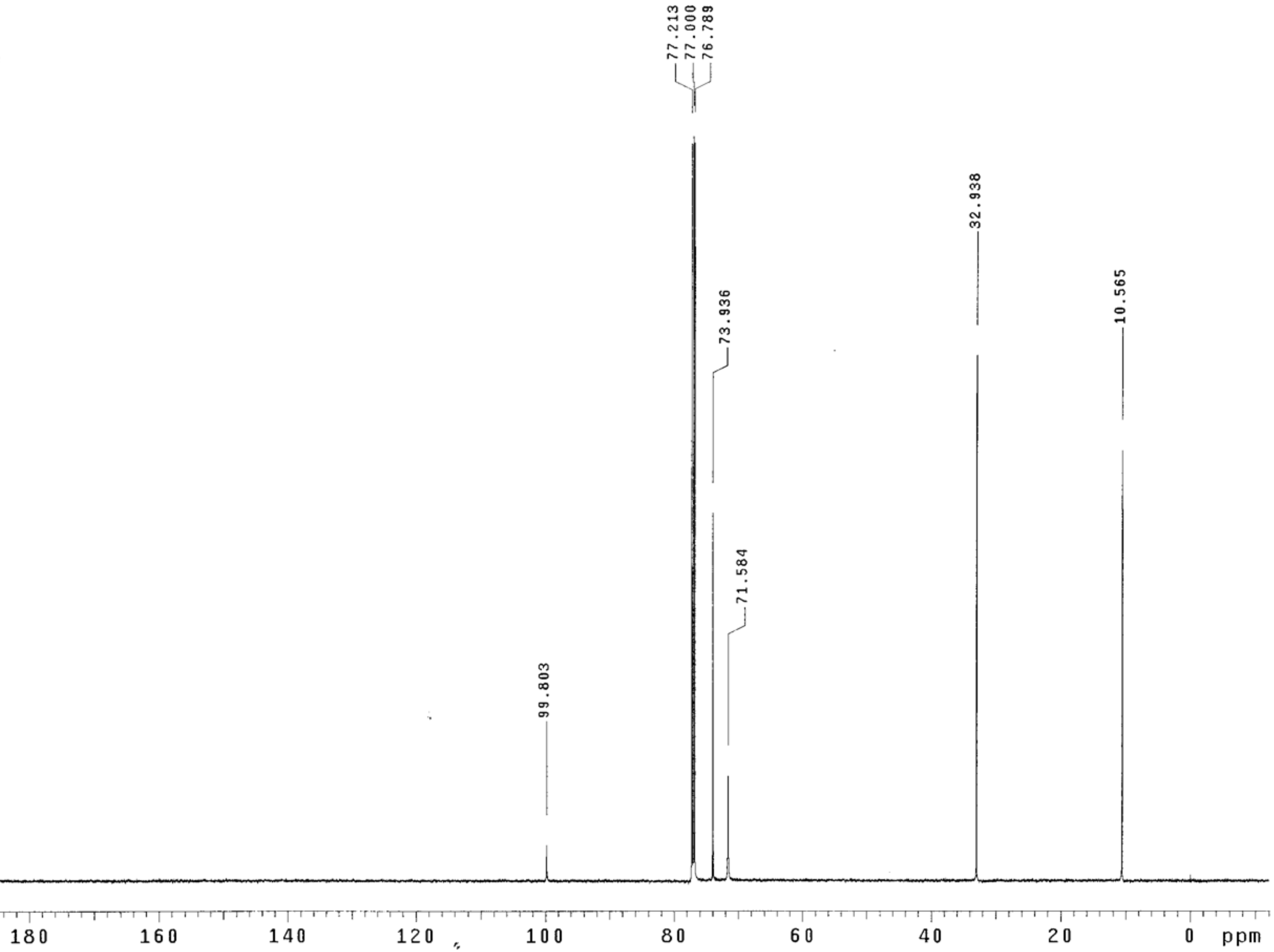

Shimadzu CLASS-VP V6.14 SP1 ***Area % Report***

Method Name: C:\CLASS-VP\untitled.met
Data Name: D:\CLASS-VP6.14\Data\Xu\2e-0.4iPrOH-1mL.dat
User: System
Acquired: 2016-12-5 15:15:43
Printed: 2017-1-6 20:01:55

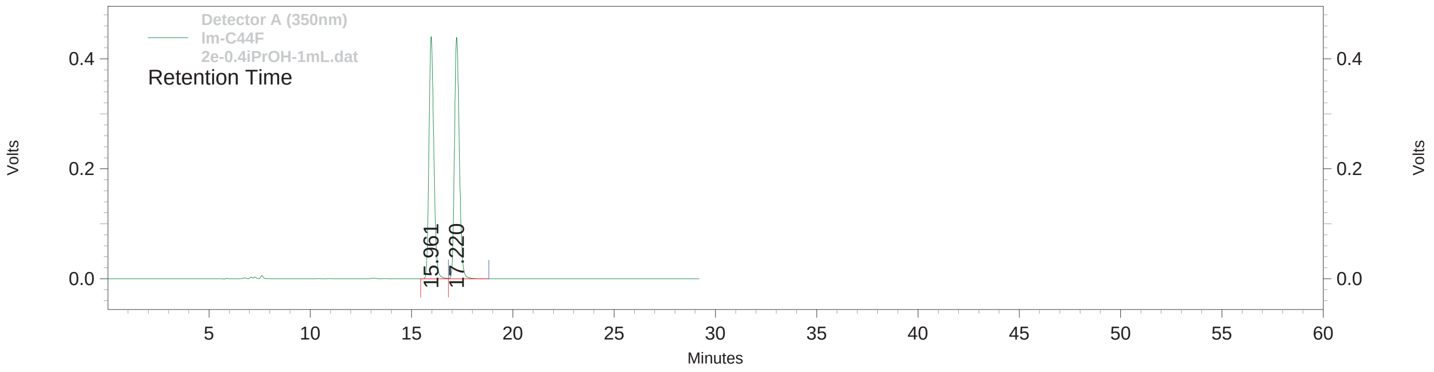

Detector A (350nm)

Pk #	Retention Time	Area	Area %	Height	Height %
1	15.961	7354877	50.005	440714	50.091
2	17.220	7353289	49.995	439117	49.909
Totals		14708166	100.000	879831	100.000

HPLC analysis of 2e
.
Chiralpak-IB column
eluting solvent: 2-PrOH/n-hexane = 0.4:99.6
flow rate: 1 mL/min
column temperature: 25°C
detection wavelength: 350 nm
.
retention factors: 3.988, 4.381
selective factor: 1.099

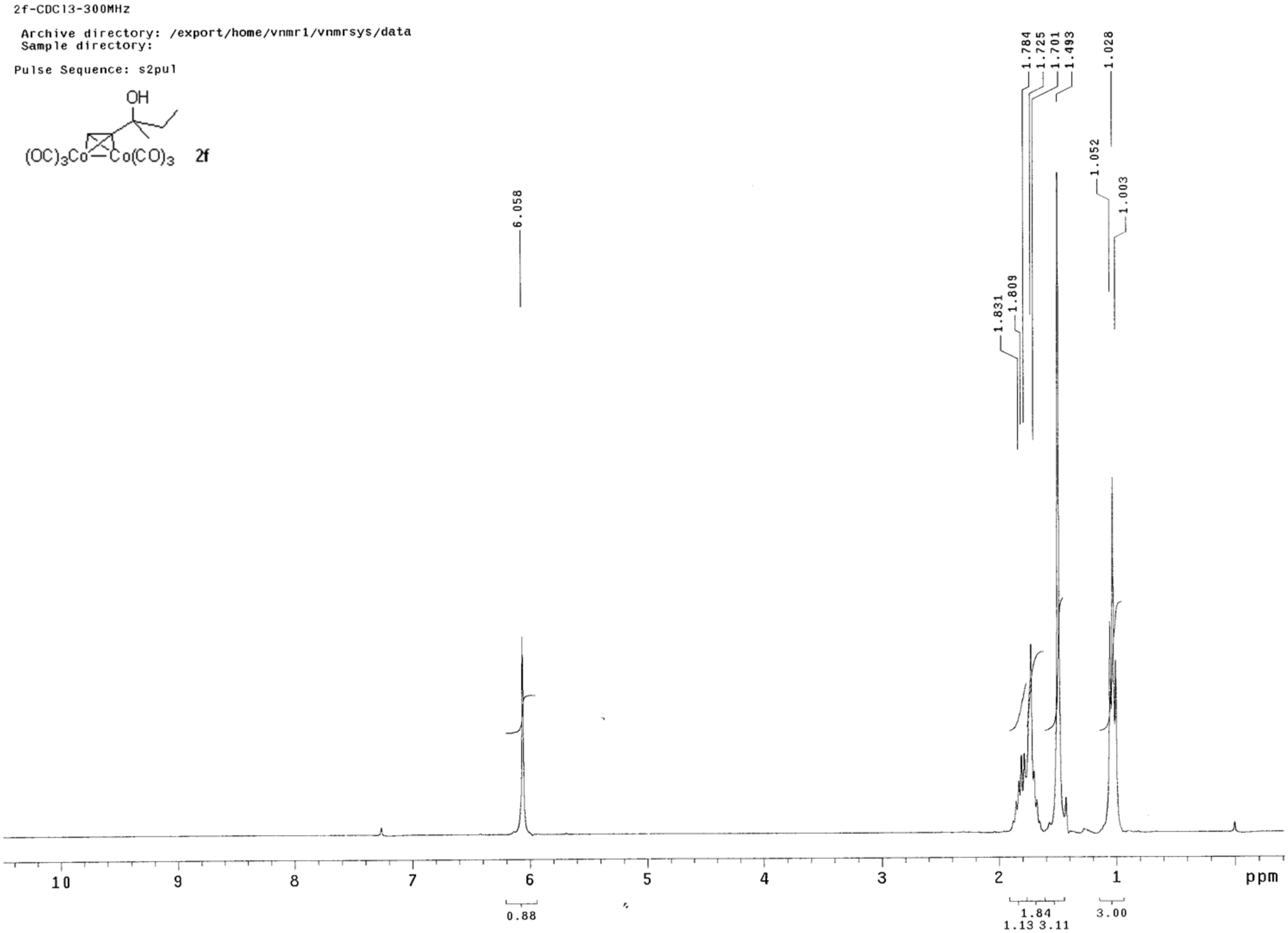
2f-CDCl3-300MHz
Archive directory: /export/home/vnmr1/vnmrsys/data
Sample directory:
Pulse Sequence: s2pul
OH
(OC)3Co—Co(CO)3
2f
6.058
1.831
1.809
1.784
1.725
1.701
1.493
1.052
1.028
1.003
10
9
8
7
6
5
4
3
2
1
ppm
0.88
1.84
1.13 3.11
3.00

2f-CDCl3-300MHz

Archive directory: /export/home/vnmr1/vnmrsys/data
Sample directory:

Pulse Sequence: s2pul

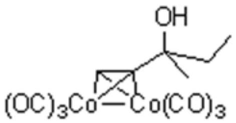

2f

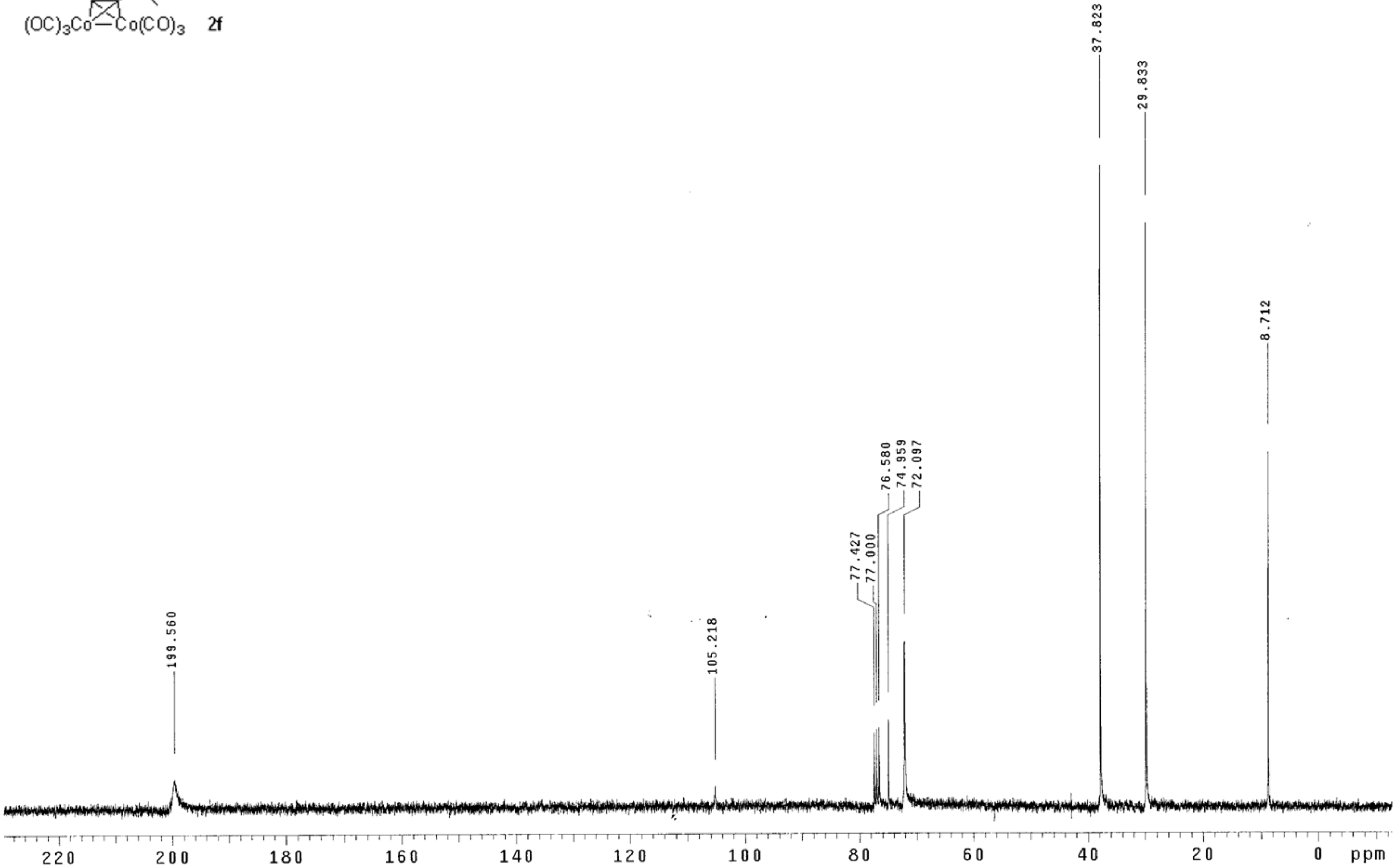

Method Name: **C:\CLASS-VP\untitled.met**
Data Name: **D:\CLASS-VP6.14\Data\Xu\2f-0.4iPrOH-1mL-IA.dat**
User: **System**
Acquired: **2016-12-9 10:28:18**
Printed: **2017-1-6 20:07:03**

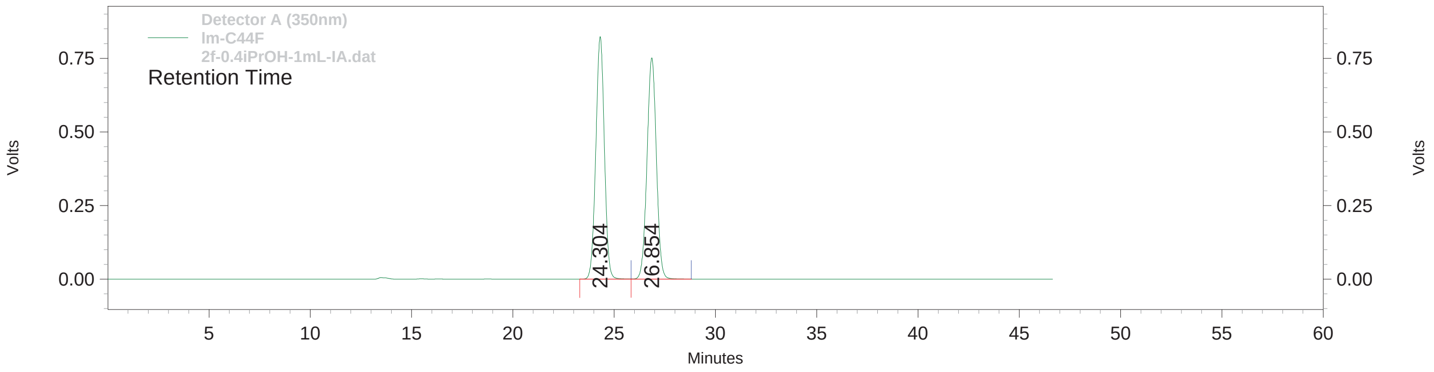

Detector A (350nm)

Pk #	Retention Time	Area	Area %	Height	Height %
1	24.304	24231407	49.944	823853	52.303
2	26.854	24285445	50.056	751314	47.697
Totals		48516852	100.000	1575168	100.000

Chiralpak-IA column
eluting solvent: 2-PrOH/n-hexane = 0.4:99.6
flow rate: 1 mL/min
column temperature: 25°C
detection wavelength: 350 nm
.
retention factors: 6.815, 7.635
selective factor: 1.120

2g-CDCl3-300MHz

Archive directory: /export/home/vnmr1/vnmrsys/data
Sample directory:

Pulse Sequence: s2pul

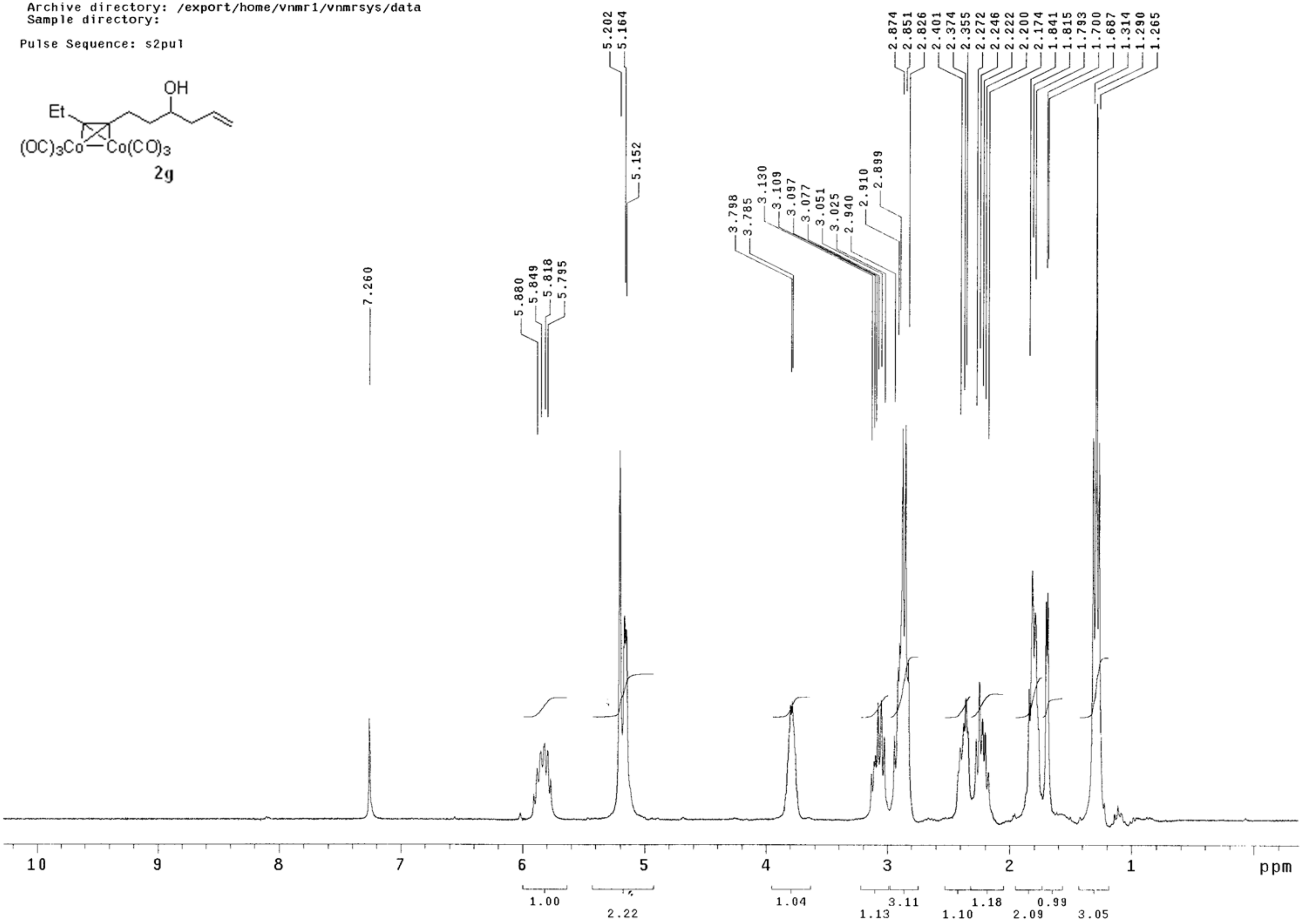

2g-CDCl3-300MHz

Archive directory: /export/home/vnmr1/vnmrsys/data
Sample directory:

Pulse Sequence: s2pul

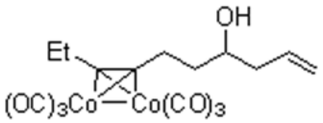

2g

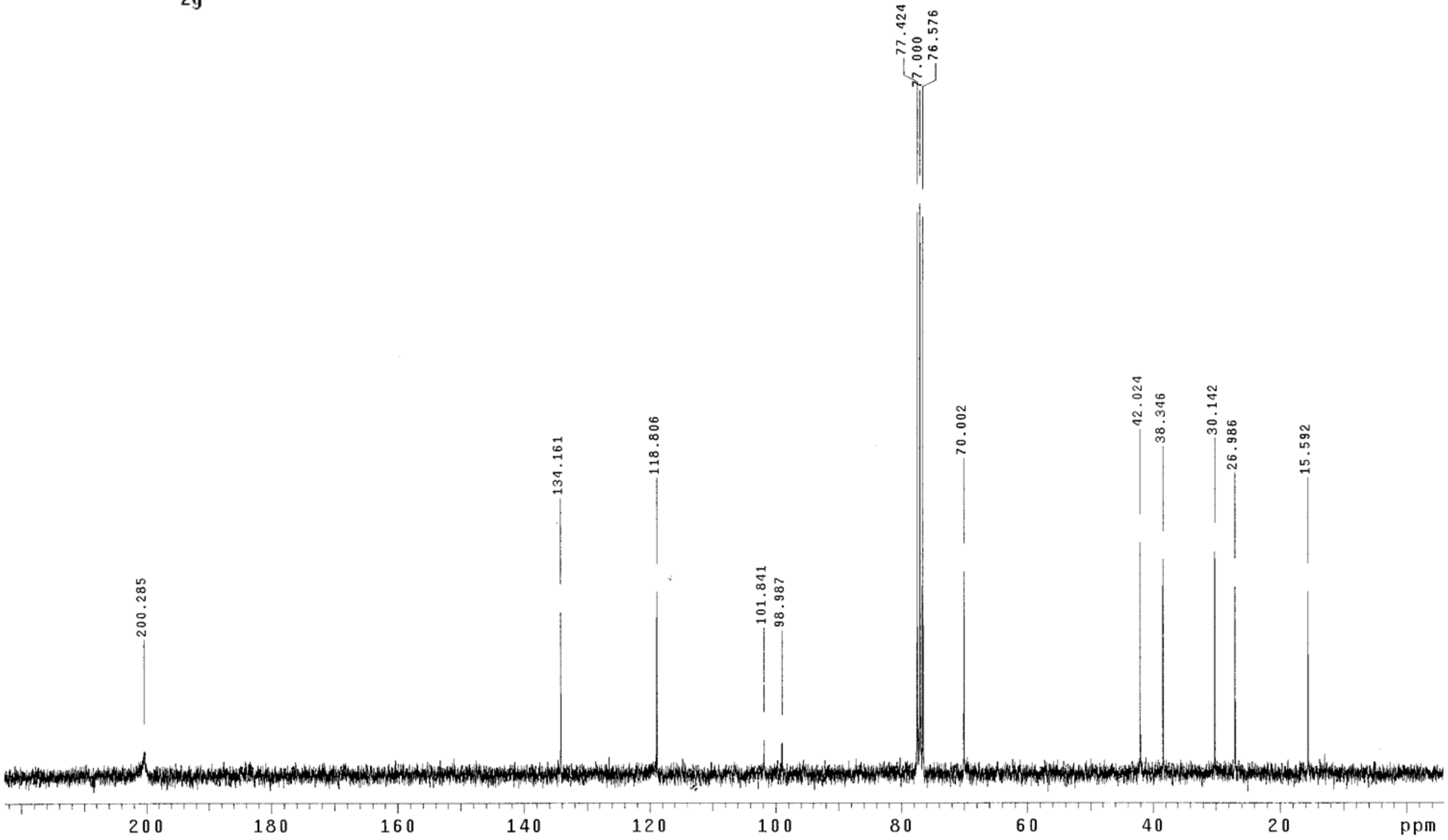

Method Name: **C:\CLASS-VP\untitled.met**
Data Name: **D:\CLASS-VP6.14\Data\Xu\2g-0.5iPrOH-1mL-350nm-IB.dat.dat**
User: **System**
Acquired: **2017-1-10 9:58:57**
Printed: **2017-1-18 15:31:30**

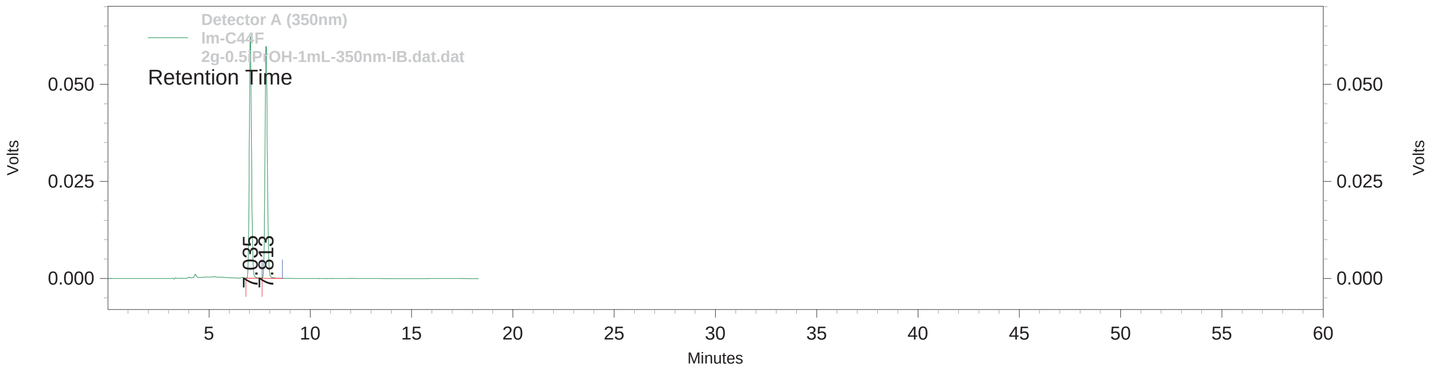

Detector A (350nm)

Pk #	Retention Time	Area	Area %	Height	Height %
1	7.035	479562	50.013	62149	50.992
2	7.813	479316	49.987	59729	49.008
Totals		958878	100.000	121878	100.000

HPLC analysis of 2g
.
Chiralpak-IB column
eluting solvent: 2-PrOH/n-hexane = 0.5:99.5
flow rate: 1 mL/min
column temperature: 25°C
detection wavelength: 350 nm
.
retention factors: 1.198, 1.441
selective factor: 1.203

2h-CDCl3-300MHz

Archive directory: /export/home/vnmr1/vnmrsys/data
Sample directory:

Pulse Sequence: s2pul

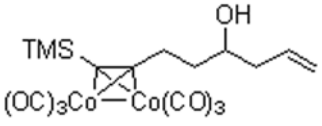

2h

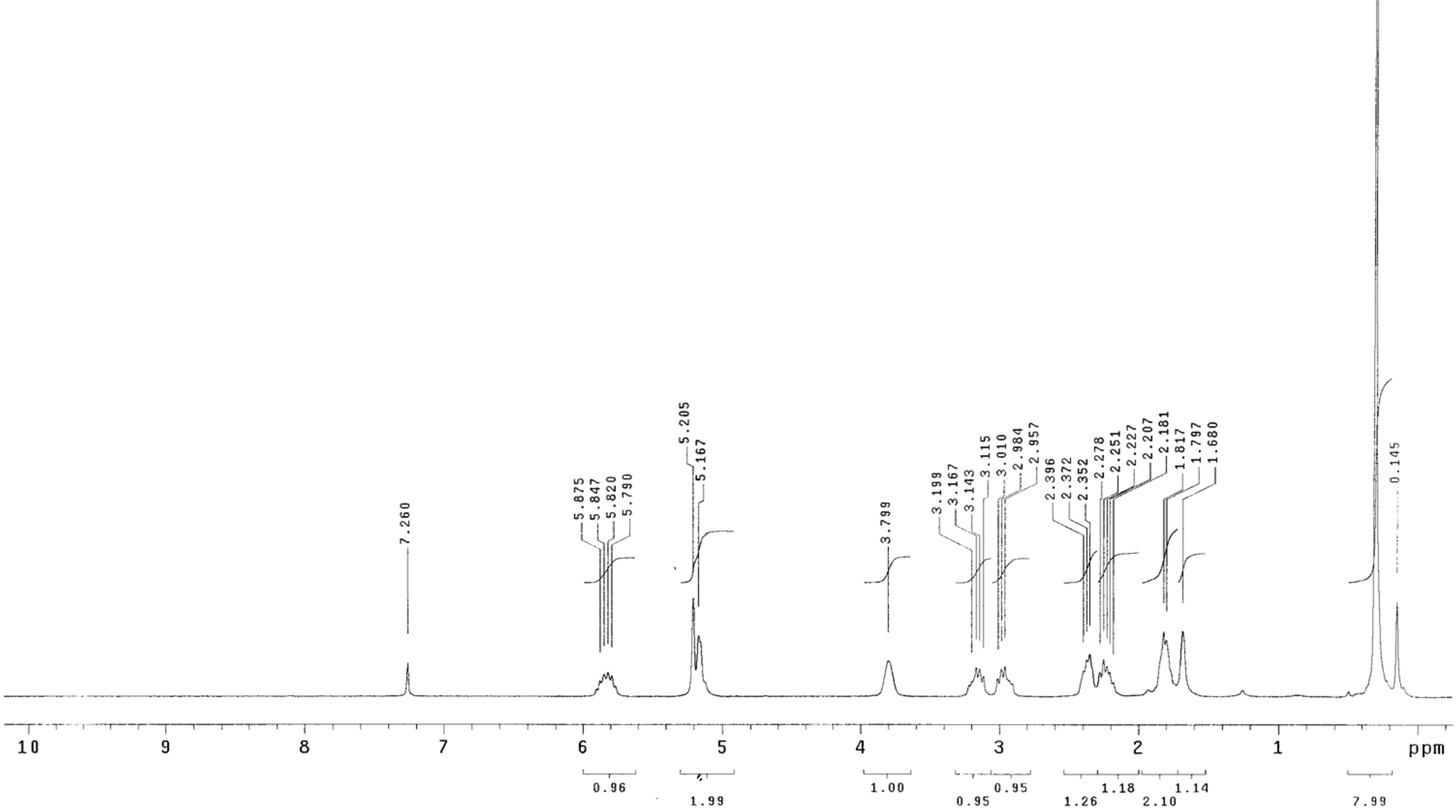

2h-CDCl3-300MHz

Archive directory: /export/home/vnmr1/vnmrsys/data
Sample directory:

Pulse Sequence: s2pul

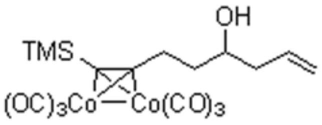

2h

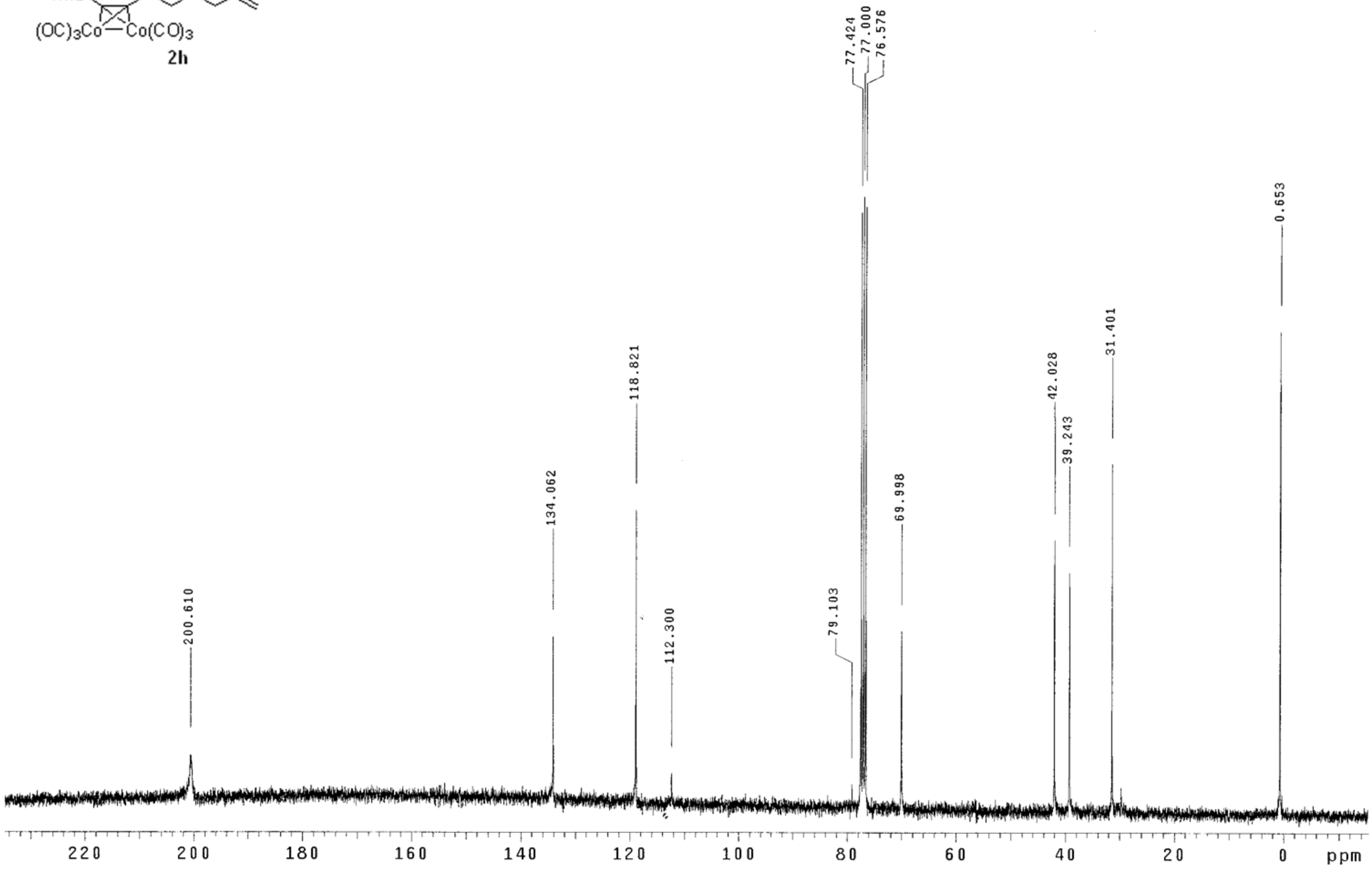

Method Name: C:\CLASS-VP\untitled.met
Data Name: D:\CLASS-VP6.14\Data\Xu\2h-0.3iPrOH-1mL.dat
User: System
Acquired: 2016-12-20 8:18:50
Printed: 2017-1-18 15:33:21

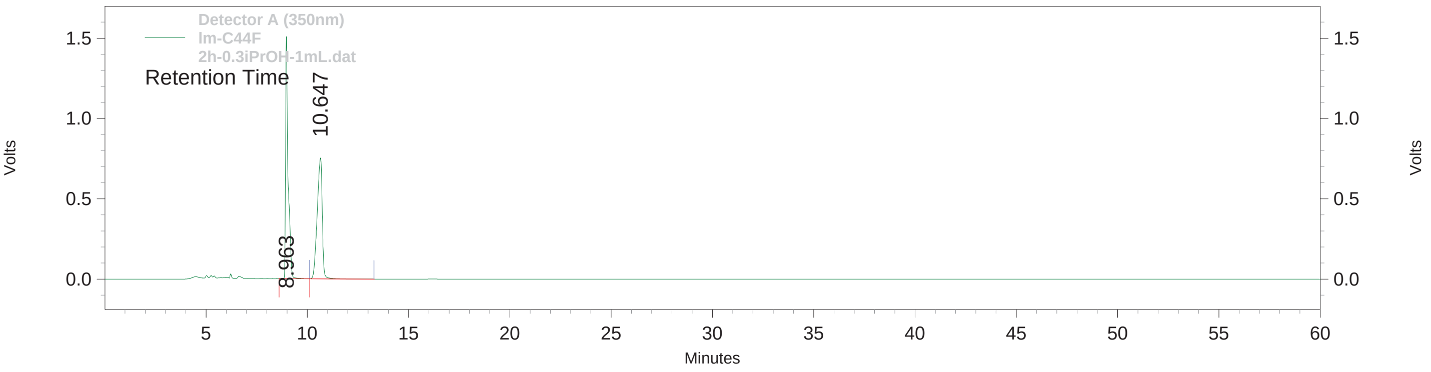

Detector A (350nm)

Pk #	Retention Time	Area	Area %	Height	Height %
1	8.963	12932161	49.699	1507716	66.686
2	10.647	13088602	50.301	753217	33.314
Totals		26020763	100.000	2260934	100.000

HPLC analysis of 2h
.
Chiralpak-IB column
eluting solvent: 2-PrOH/n-hexane = 0.3:99.7
flow rate: 1 mL/min
column temperature: 25°C
detection wavelength: 350 nm
.
retention factors: 1.801, 2.327
selective factor: 1.292

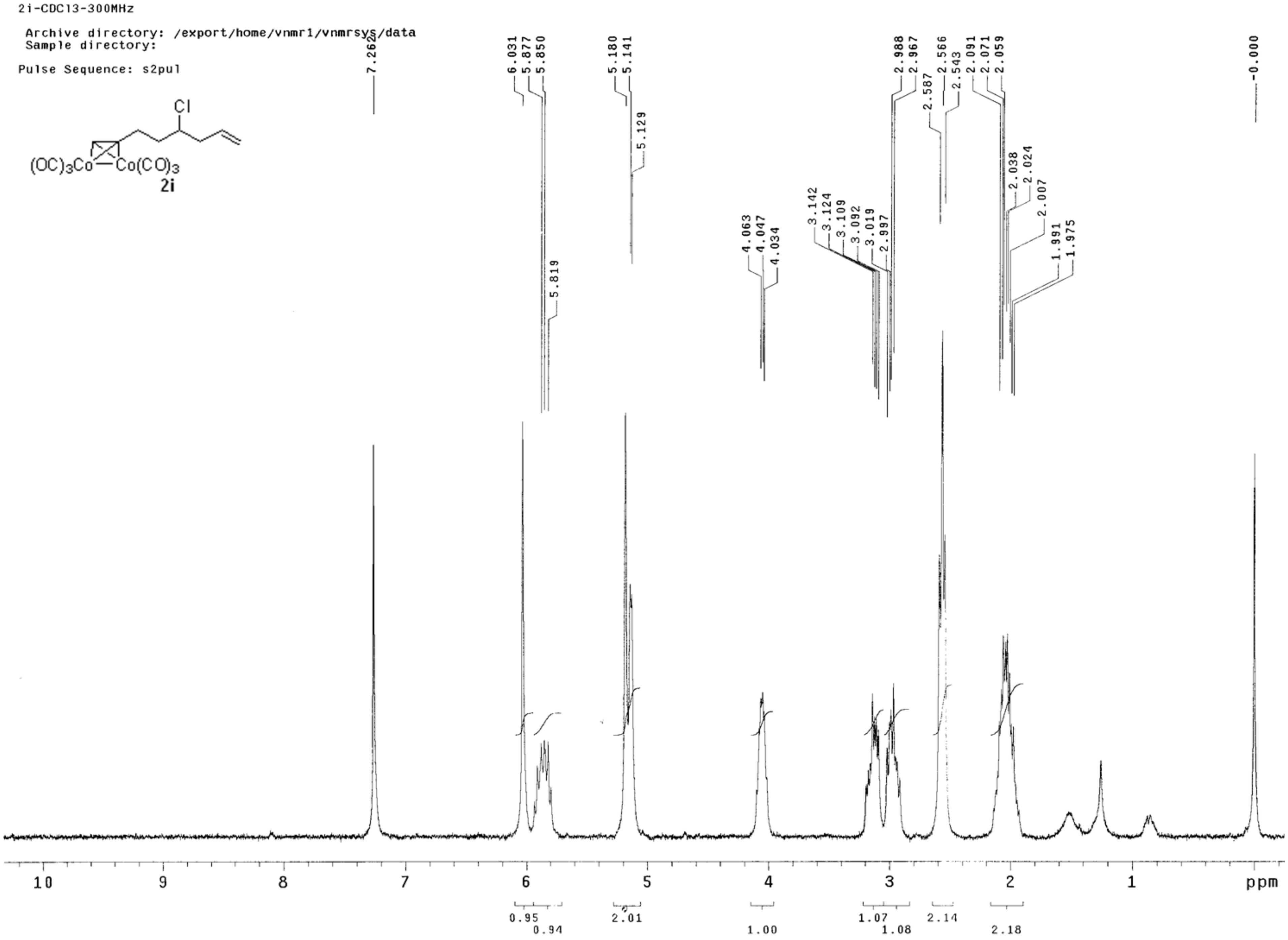
2i-CDCl3-300MHz
Archive directory: /export/home/vnmr1/vnmrsys/data
Sample directory:
Pulse Sequence: s2pul
Cl
(OC)3Co
Co(CO)3
2i
7.262
6.031
5.877
5.850
5.819
5.180
5.141
5.129
4.063
4.047
4.034
3.142
3.124
3.109
3.092
3.019
2.997
2.988
2.967
2.587
2.566
2.543
2.091
2.071
2.059
2.038
2.024
2.007
1.991
1.975
-0.000
10
9
8
7
6
5
4
3
2
1
ppm
0.95
0.94
2.01
1.00
1.07
1.08
2.14
2.18

2i-CDCl3-300MHz

Archive directory: /export/home/vnmr1/vnmrsys/data
Sample directory:

Pulse Sequence: s2pul

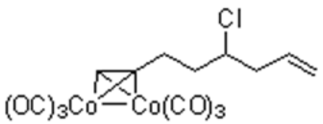

2i

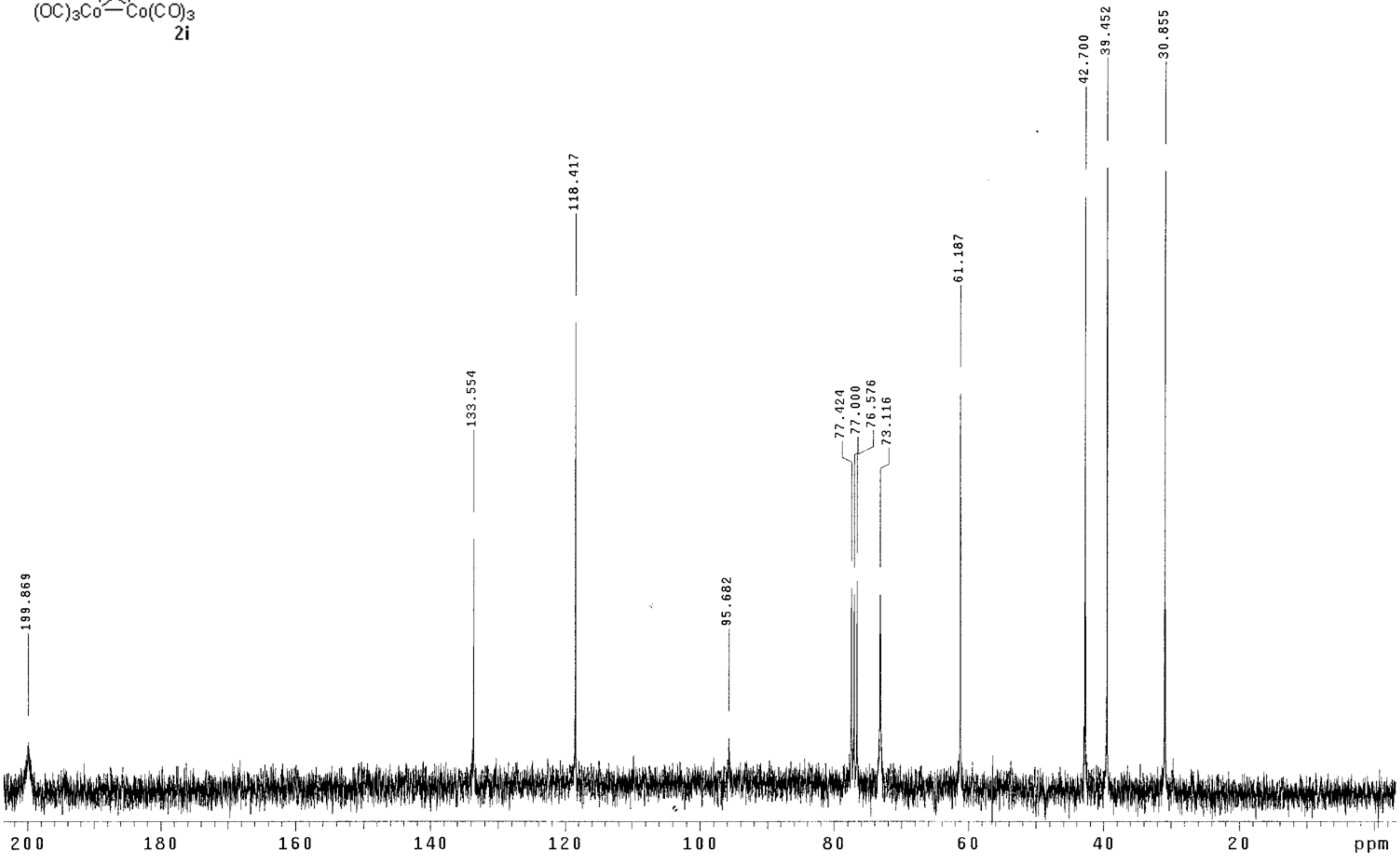

Method Name: **C:\CLASS-VP\untitled.met**
Data Name: **D:\CLASS-VP6.14\Data\Xu\2i-0.05EtOAc-0.3mL-0degree-IB.dat**
User: **System**
Acquired: **2017-1-5 12:35:45**
Printed: **2017-1-18 15:34:27**

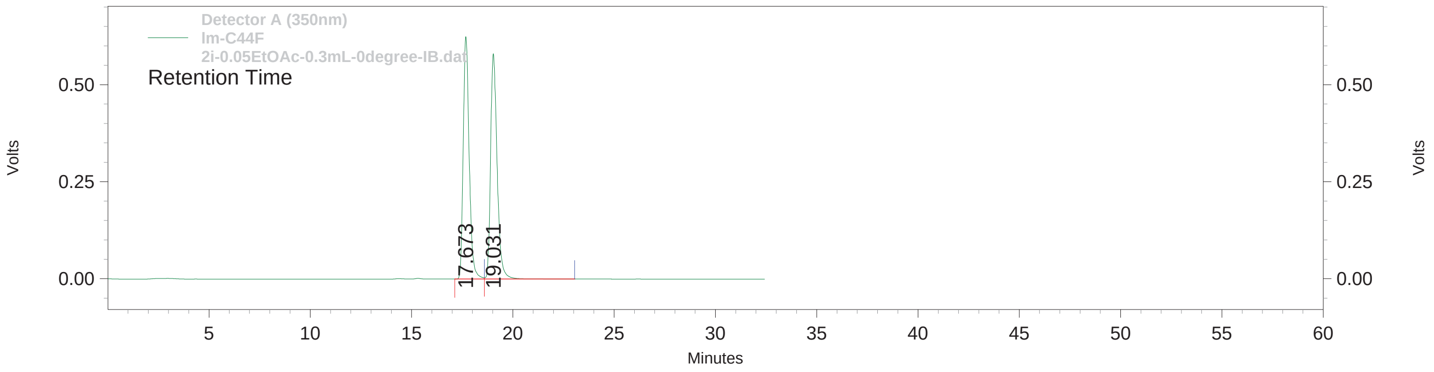

Detector A (350nm)

Pk #	Retention Time	Area	Area %	Height	Height %
1	17.673	12770343	49.463	624814	51.834
2	19.031	13047813	50.537	580604	48.166

Totals					
		25818156	100.000	1205419	100.000

HPLC analysis of 2i
.
Chiralpak-IB column
eluting solvent: EtOAc/n-hexane = 0.05:99.95
flow rate: 0.3 mL/min
column temperature: 0°C
detection wavelength: 350 nm
.
retention factors: 0.592, 0.715
selective factor: 1.208

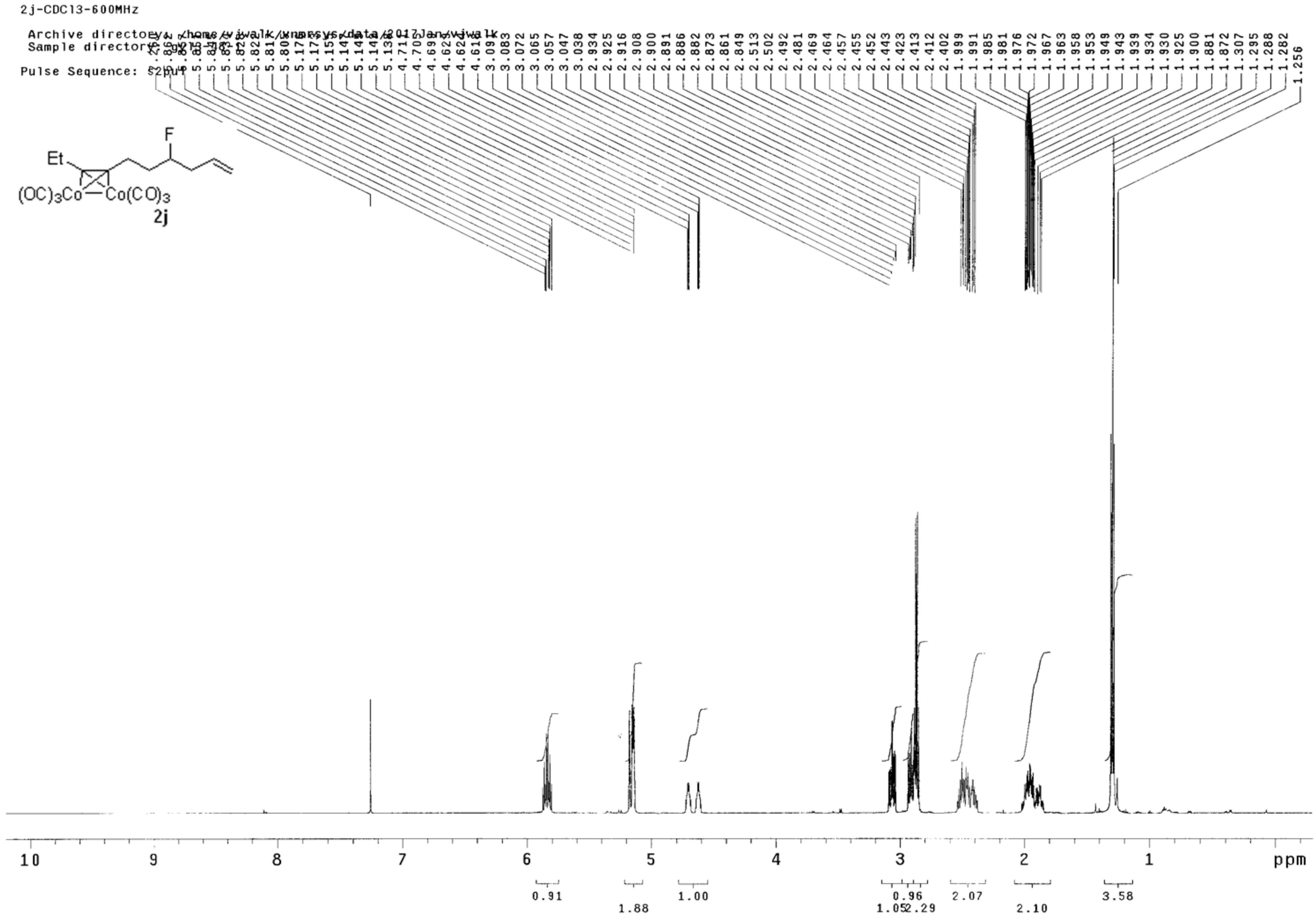

2j-CDCl3-600MHz
Archive directory: /home/vjwalk/vnmrsys/data/2017Jan/vjwalk
Pulse Sequence: s2pul
3.083
3.072
3.065
3.057
3.047
3.038
2.934
2.925
2.916
2.908
2.900
2.891
2.886
2.882
2.873
2.861
2.849
2.513
2.502
2.492
2.481
2.469
2.464
2.457
2.455
2.452
2.443
2.423
2.413
2.412
2.402
1.999
1.991
1.985
1.981
1.976
1.972
1.967
1.963
1.958
1.953
1.949
1.943
1.939
1.934
1.930
1.925
1.900
1.881
1.872
1.307
1.295
1.288
1.282
1.256
Et
F
(OC)3Co
Co(CO)3
2j
10
9
8
7
6
5
4
3
2
1
ppm
0.91
1.88
1.00
0.96
1.05
2.29
2.07
2.10
3.58

2j-CDCl3-600MHz

Archive directory: /home/vjwalk/vnmrsys/data/2017Jan/vjwalk
Sample directory: gyl3-48-1

Pulse Sequence: s2pul

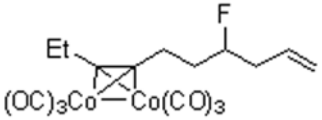

2j

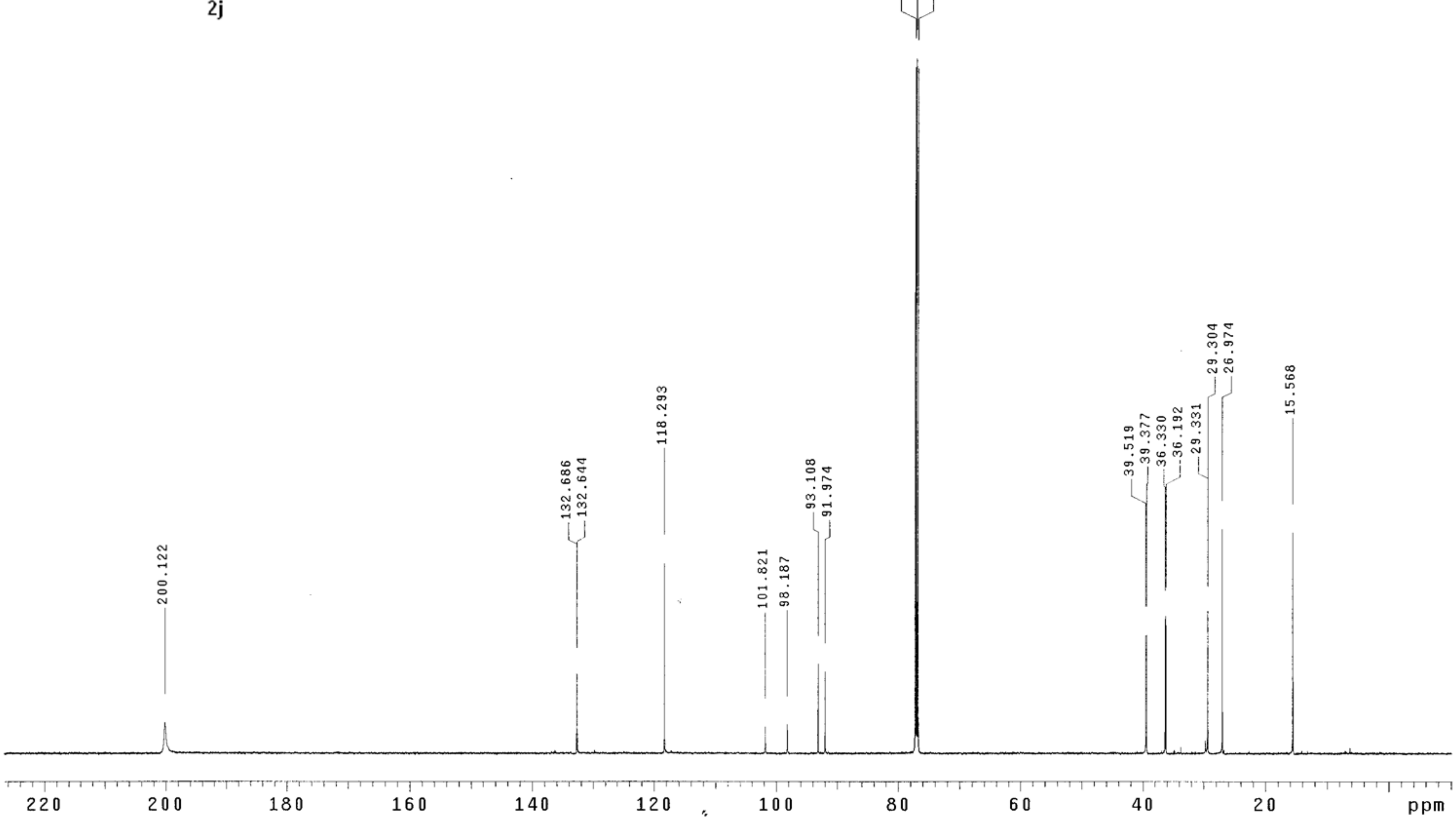

Method Name: {Method Name}
Data Name: D:\CLASS-VP6.14\Data\Xu\2j-0.05DCM-0.4mL-10degree-IB.dat
User: System
Acquired: 2017-1-9 11:45:14
Printed: 2017-3-6 14:40:08

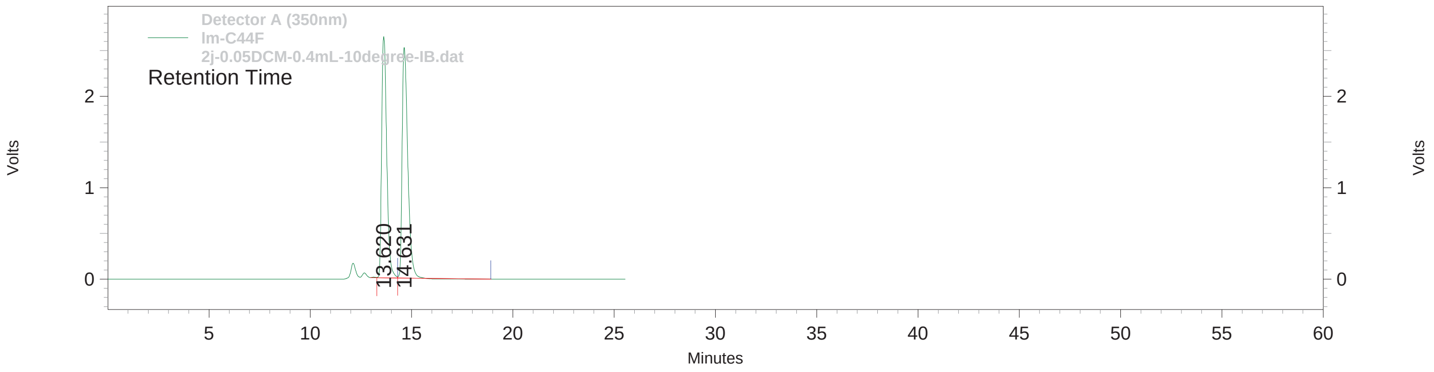

Detector A (350nm)

Pk #	Retention Time	Area	Area %	Height	Height %
1	13.620	45829535	48.746	2639148	51.178
2	14.631	48186864	51.254	2517634	48.822
Totals		94016399	100.000	5156782	100.000

HPLC analysis of 2j
.
Chiralpak-IB column
eluting solvent: CH_2Cl_2/n-hexane = 0.05:99.95
flow rate: 0.4 mL/min
column temperature: 10°C
detection wavelength: 350 nm
.
retention factors: 0.584, 0.701
selective factor: 1.200

2k-CDCl3-500MHz

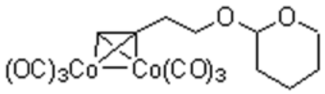

2k

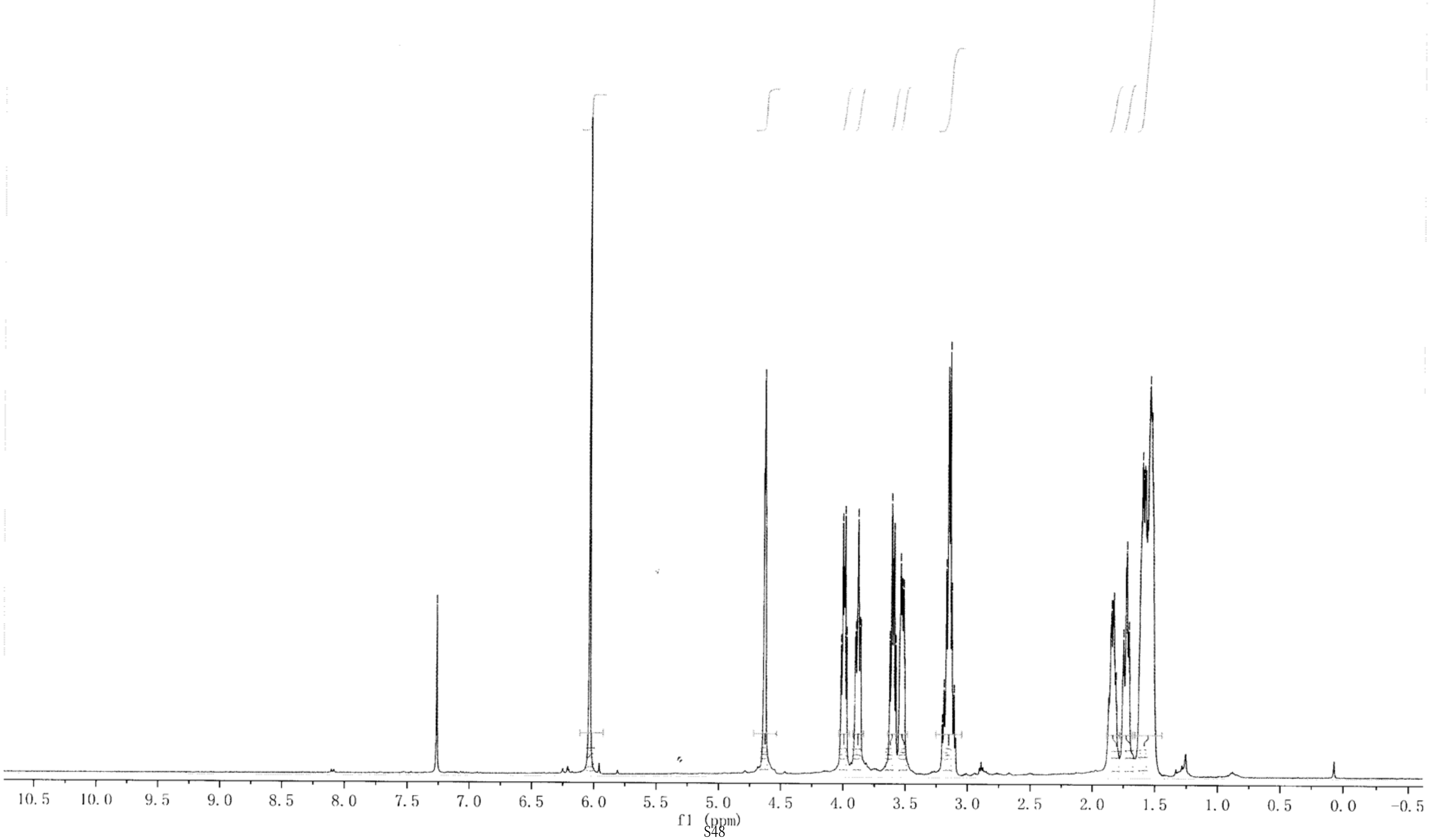

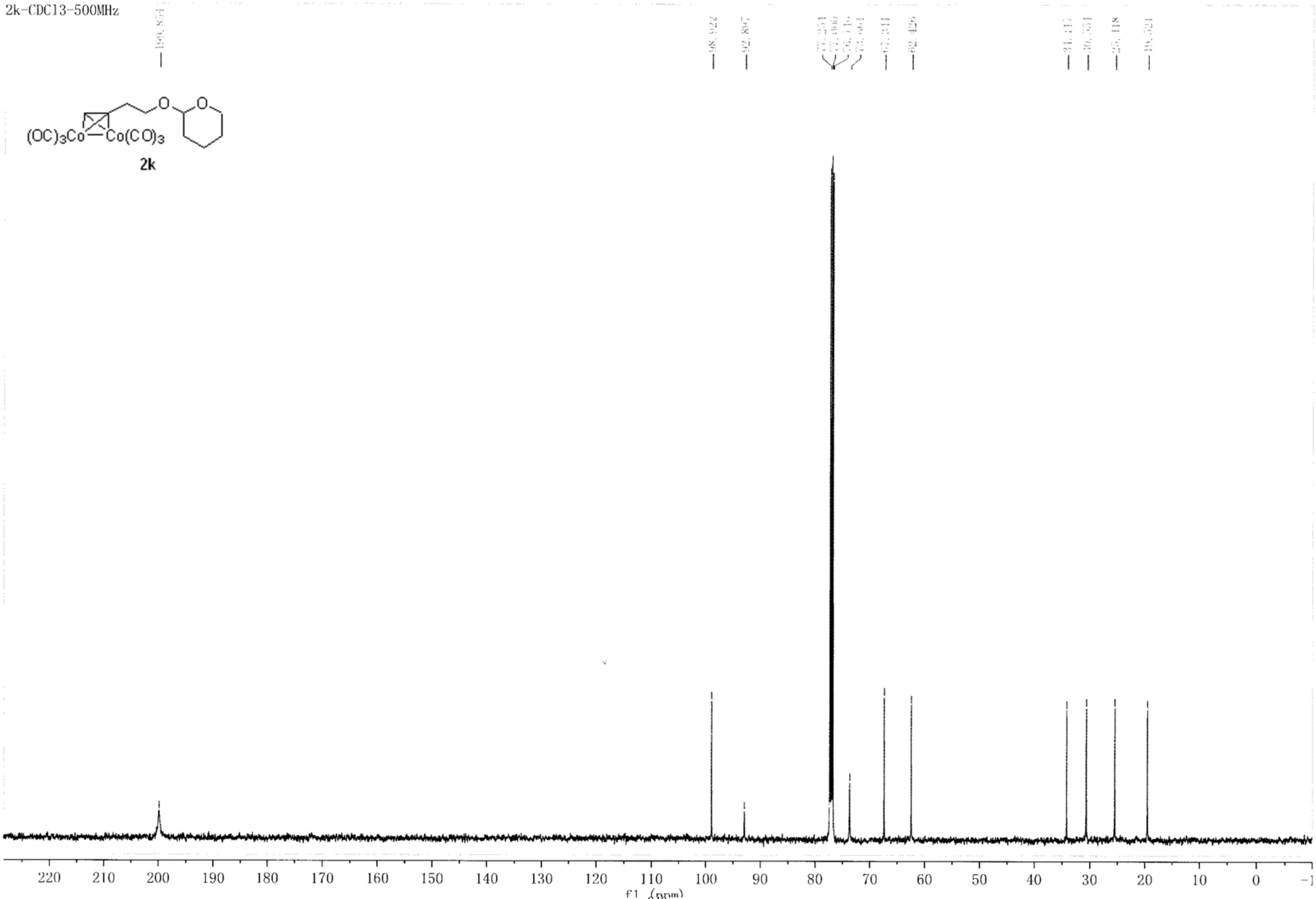
2k-CDCl3-500MHz
98.922
92.847
62.426
(OC)3Co
Co(CO)3
2k
220 210 200 190 180 170 160 150 140 130 120 110 100 90 80 70 60 50 40 30 20 10 0
f1 (ppm)

Method Name: C:\CLASS-VP\untitled.met
Data Name: D:\CLASS-VP6.14\Data\Xu\2k-0.1iPrOH-1mL.dat
User: System
Acquired: 2016-12-15 11:19:56
Printed: 2017-1-18 15:36:14

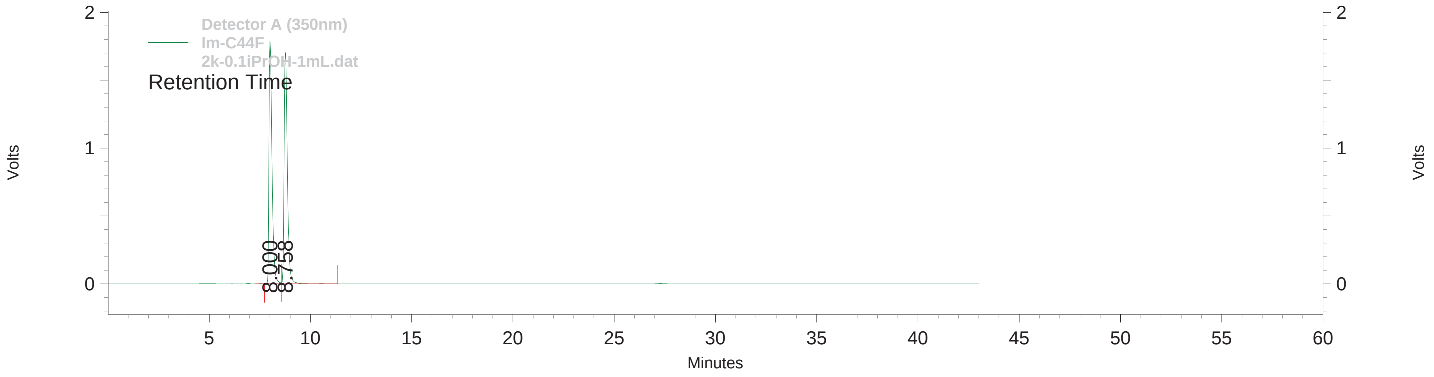

Detector A (350nm)

Pk #	Retention Time	Area	Area %	Height	Height %
1	8.000	18675391	49.498	1782030	51.208
2	8.758	19053910	50.502	1697976	48.792
Totals		37729302	100.000	3480005	100.000

HPLC analysis of 2k
.
Chiralpak-IB column
eluting solvent: 2-PrOH/n-hexane = 0.1:99.9
flow rate: 1 mL/min
column temperature: 25°C
detection wavelength: 350 nm
.
retention factors: 1.500, 1.737
selective factor: 1.158

6. HPLC monitor of the reaction of **1a** and $Co_2(CO)_8$

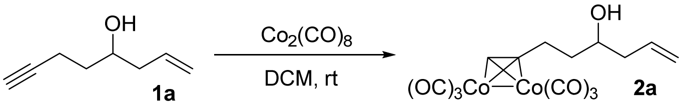

The next 3 pages are HPLC charts monitoring the reaction of **1a**, and $Co_2(CO)_8$ in CH_2Cl_2,

HPLC conditions: CHIRALPAK-IB column, 0.4:99.6 iPrOH/*n*-hexane, 1 mL·min^{-1} flow rate, and 350 nm detection wavelength at 25°C.

Sample preparation: 20 μL of the reaction mixture was diluted with 0.5 mL *n*-hexane, passed through a disposable syringe filter (Nylon 66, 0.22 μm, 13 mm). 5 μL of the filtration was injected.

S52: $Co_2(CO)_8$ in CH_2Cl_2 before addition of **1a**;
S53: 5 min after addition of **1a**;
S54: 1 h after addition of **1a**.

Shimadzu CLASS-VP V6.14 SP1 ***Area % Report***

Method Name: C:\CLASS-VP\untitled.met
Data Name: D:\CLASS-VP6.14\Data\Xu\wj4-8-Co-0.4iPrOH-1mL-1.dat
User: System
Acquired: 2017-1-10 10:48:40
Printed: 2017-1-18 17:15:35

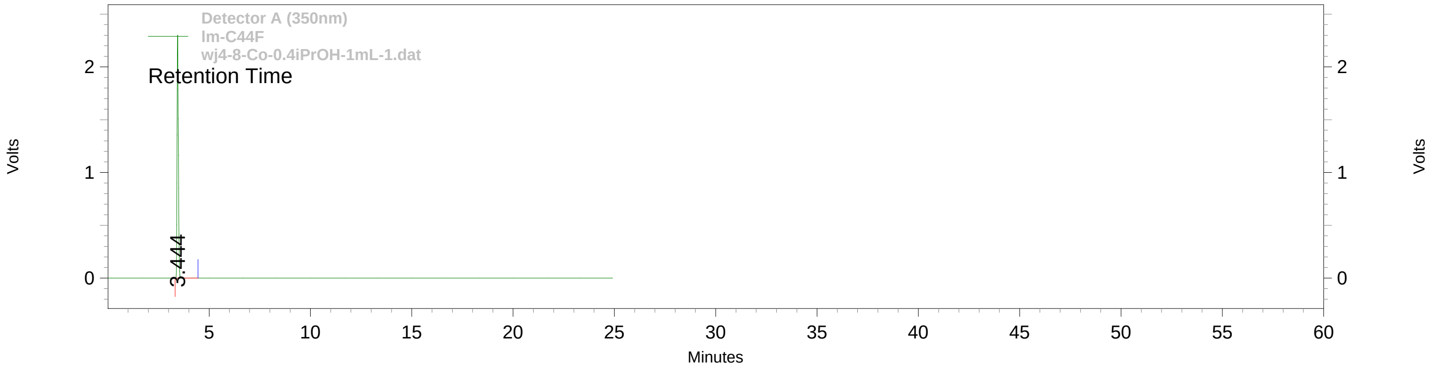

Detector A (350nm)

Pk #	Retention Time	Area	Area %	Height	Height %
1	3.444	10474869	100.000	2301258	100.000

Totals					
		10474869	100.000	2301258	100.000

Method Name: C:\CLASS-VP\untitled.met
Data Name: D:\CLASS-VP6.14\Data\Xu\wj4-8-5min-0.4iPrOH-1mL.dat
User: System
Acquired: 2017-1-10 11:14:58
Printed: 2017-1-18 17:10:47

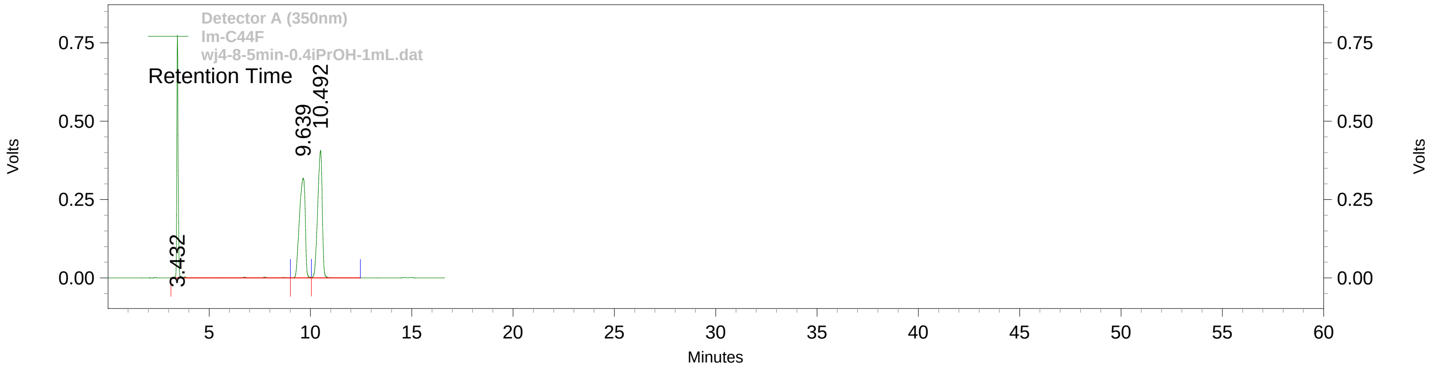

Detector A (350nm)

Pk #	Retention Time	Area	Area %	Height	Height %
1	3.432	3540718	21.784	774668	51.659
2	9.639	6341408	39.016	318152	21.216
3	10.492	6371285	39.200	406760	27.125
Totals		16253411	100.000	1499580	100.000

Method Name: C:\CLASS-VP\untitled.met
Data Name: D:\CLASS-VP6.14\Data\Xu\wj4-8-1h-0.4iPrOH-1mL.dat
User: System
Acquired: 2017-1-10 12:19:05
Printed: 2017-1-18 17:14:35

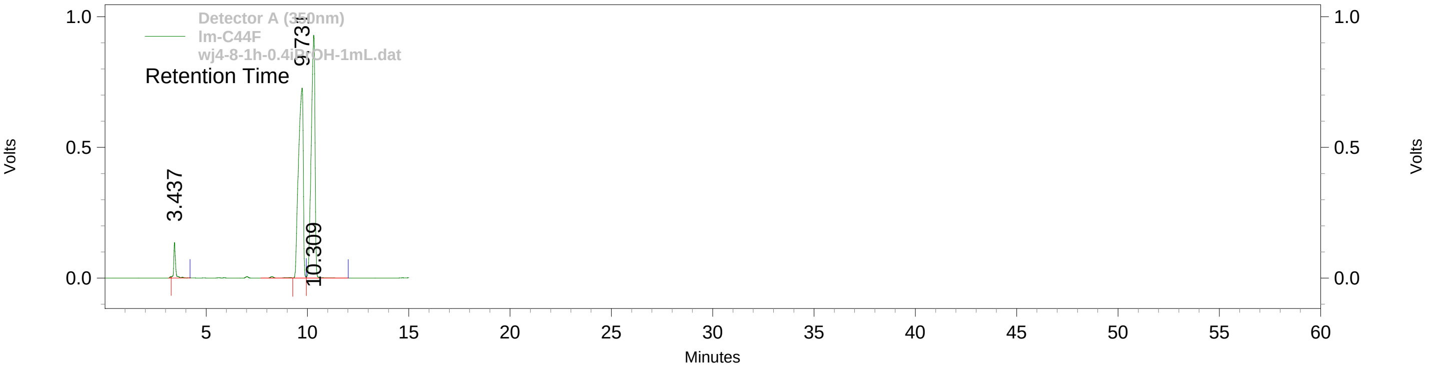

Detector A (350nm)

Pk #	Retention Time	Area	Area %	Height	Height %
1	3.437	796748	3.230	136158	7.596
2	9.731	11919039	48.317	726707	40.543
3	10.309	11952799	48.454	929573	51.861

Totals					
		24668586	100.000	1792438	100.000
